# Advancing the next generation of high-performance metal matrix composites through metal particle reinforcement

**DOI:** 10.1007/s42114-024-01057-4

**Published:** 2024-11-27

**Authors:** Sayed Ali Ahmad Alem, Mohammad Hossein Sabzvand, Parnian Govahi, Pooria Poormehrabi, Mahdi Hasanzadeh Azar, Sara Salehi Siouki, Reza Rashidi, Shayan Angizi, Sara Bagherifard

**Affiliations:** 1 Department of Chemistry, Materials and Chemical Engineering “Giulio Natta” (DCMC), Politecnico Di Milano, Milan, Italy; 2 Department of Polymer Engineering and Science, Montanuniversitaet Leoben, Leoben, Austria; 3 Department of Engineering Physics, McMaster University, Hamilton, Canada; 4 Department of Mechanical and Mechatronics Engineering, University of Waterloo, Waterloo, ON Canada; 5 Federal Institute for Materials Research and Testing (BAM), Berlin, Germany; 6 Department of Chemistry and Chemical Biology, McMaster University, Hamilton, Canada; 7 Department of Chemical Engineering, McMaster University, 1280 Main Street West, Hamilton, ON L8S 4L8 Canada; 8 Department of Mechanical Engineering, Politecnico Di Milano, Milan, Italy

**Keywords:** Composite, Metal matrix composite, Interface, High entropy alloy, Bulk metallic glass, Intermetallic, Shape memory alloys, Microstructure, Mechanical properties, Reinforcement

## Abstract

Metal matrix composites (MMCs) offer asignificant boost to achieve a wide range of advanced mechanical properties and improved performance for a variety of demanding applications. The addition of metal particles as reinforcement in MMCs is an exciting alternative to conventional ceramic reinforcements, which suffer from numerous shortcomings. Over the last two decades, various categories of metal particles, i.e., intermetallics, bulk metallic glasses, high-entropy alloys, and shape memory alloys, have become popular as reinforcement choices for MMCs. These groups of metal particles offer a combination of outstanding physico-mechanical properties leading to unprecedented performances; moreover, they are significantly more compatible with the metal matrices compared to traditional ceramic reinforcements. In this review paper, the recent developments in MMCs are investigated. The importance of understanding the active mechanisms at the interface of the matrix and the reinforcement is highlighted. Moreover, the processing techniques required to manufacture high-performance MMCs are explored identifying the potential structural and functional applications. Finally, the potential advantages and current challenges associated with the use of each reinforcement category and the future developments are critically discussed. Based on the reported results, the use of metal particles as reinforcement in MMCs offers a promising avenue for the development of advanced materials with novel mechanical properties. Further progress requires more in-depth fundamental research to realize the active reinforcing mechanisms at the atomic level to precisely identify, understand, and tailor the properties of the integrated composite materials.

## Introduction

Metal matrix composites (MMCs) consist of a metal matrix reinforced by one or more secondary phases, such as particles, fibers, or whiskers. They can render significantly higher mechanical and functional properties in comparison to their monolithic metallic counterparts, making MMCs promising materials for a wide range of applications [1–5]. The increasing demand for high performance compounds in various industries, e.g., aerospace, transportation, electronic packaging and thermal management, recreational products and sporting goods, energy, and biomedical devices has motivated the rapid development of novel MMCs [6–11]. Despite the high potential, further advancement in the field of MMCs requires solutions to address the major technical challenges, including lack of efficient material design approaches, limited compositional control especially at the interfaces, and the need for cost-effective fabrication methods [12–15].

Conventional reinforcements of MMCs are ceramic particles (i.e., oxides, carbides, nitrides, borides, etc.), which are ionic and/or covalent compounds, as opposed to the metallic matrices characterized with the metallic bond between their atoms; the covalent/ionic bonds lead to high strength, high Young’s modulus, high hardness, low deformability, low electrical thermal conductivity, and relatively low density [16–19]. However, there are several drawbacks associated with the use of ceramic particles. Typically, the surface energy of ceramic reinforcing particles, especially at nano-size, is higher than that of metals, making wetting and adherence between the particles and the metal matrices difficult. Partial bonding might cause premature failure as a consequence of interfacial decohesion upon loading. In addition, the high surface energy of ceramic particles can promote agglomeration due to van der Waals forces, thus significantly deteriorating the composite strength. Moreover, undesired reactions can occur at the ceramic/metal interface, resulting in the formation of unwanted phases, such as Al_4_C_3_, which are produced by the reaction between SiC and molten aluminum in an Al/SiC system [20]. The rough nature of the interface can lead to stress localization, which is detrimental to mechanical performance. In most cases, a significant difference in the coefficient of thermal expansion (CTE) between metals and ceramics can cause dimensional instability and cracking during thermal cycles. The intrinsic brittleness and limited toughness of ceramic particles further increase the risk of cracking under stress, affecting the strength, durability, and machinability of MMCs [21–24].

To mitigate the challenges associated with ceramic particles, several strategies have been proposed. One commonly used route is surface modification of ceramic particles through preheating, chemical etching, plasma treatment, or coating with a more compatible compound with the matrix to enhance the wettability and adhesion. Modifying the metal matrix by introducing various alloying elements such as Mg, Ca, Li, Zr, Ti, and P is another way to improve the wettability. Techniques like compo-casting, temperature- and pressure-induced powder metallurgy, and severe plastic deformation (SPD) offer benefits as well. Despite these efforts, controlling the size and distribution of ceramic particles can be also quite challenging. Nonetheless, considering the complications and limited efficiency of most of the current solutions, researchers have been actively looking for alternative remedies [25–31].

Within the last decade, the use of metallic particles as the reinforcement phase in metal matrices have garnered significant attention [32–36]. The primary driving force behind employing metallic particles lies in achieving a modulable particle–matrix interface. This effort aims at establishing a perfect balance between the physical and chemical properties of the matrix and the reinforcement. Such compatibility enhances performance [37, 38], leading to improved strength, ductility, and toughness, helping to prevent cracking and improving the formability [39]. Moreover, metallic particles can be processed at lower temperatures compared to ceramics, thereby reducing the fabrication cost and energy requirements for MMCs.

Furthermore, a broader range of metallic compounds is available for use as the reinforcement phase, including intermetallics, bulk metallic glasses (BMGs), high entropy alloys (HEAs), shape memory alloys (SMAs), and other metals, which can offer a broad spectrum of performance characteristics. It is worth mentioning that BMGs and HEAs, as the most eye-catching candidates of these groups, exist in hundreds of various compositions. This indicates a vast and uncharted path ahead for researchers interested in MMCs. While there are some invaluable reviews on the development of BMG–matrix and HEA–matrix composites [40–43], to the best of our knowledge, no review paper has specifically investigated the advancements in using metallic particles as the reinforcement of MMCs.

**Fig. 1:**
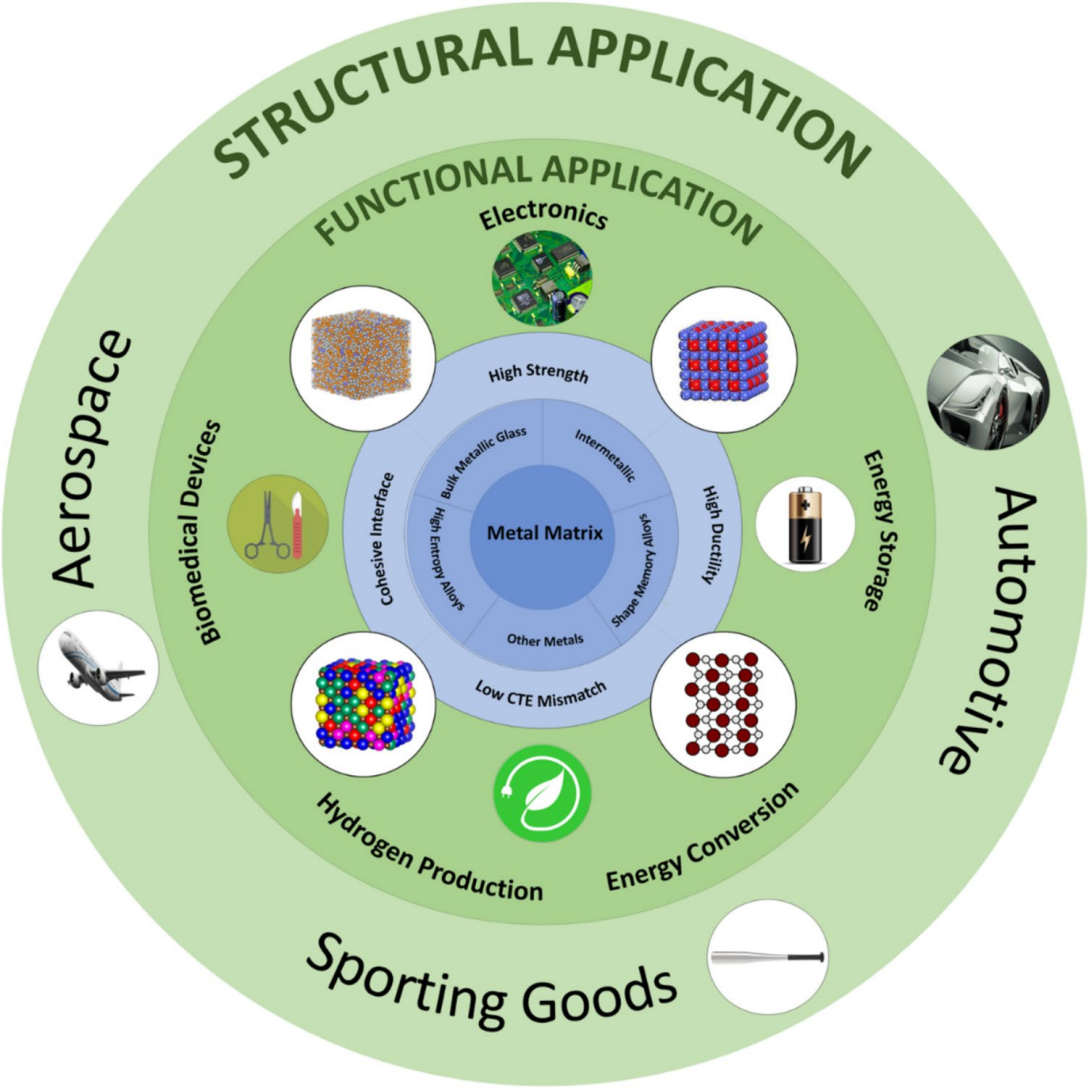
Schematic illustration of various categories of MMCs reinforced with metal particles, their main features and applications

In this review paper, the development of various MMCs reinforced by the mentioned novel categories of metallic materials is overviewed. Thereafter, the most popular fabrication techniques of metal particle reinforced MMCs are introduced. Finally, the potential applications, challenges, and prospects are discussed, trying to provide a holistic overview on the progress and advancements in this exciting field of research. Figure 1 represents the schematics illustrations of various categories of MMCs reinforced with metal particles, highlighting their main features and applications.

## Materials development

### Intermetallics

Intermetallic compounds are solid phases composed of two or more metallic or semi-metallic elements in an ordered structure, differing from alloys which generally retain the distinct structure of their constituent metallic elements [44–46]. Unlike alloys, intermetallic compounds may form bonds not solely metallic, but a combination of metallic, ionic, and covalent bonding, creating a complex bonding structure. This mixed bonding imparts exceptional properties to intermetallic compounds, including enhanced brightness, stiffness, strength, and corrosion resistance, particularly at elevated temperatures [47–51]. The distinct crystal lattice and long periodicity of intermetallic compounds, combined with the large Burgers vectors of their dislocations, contribute to relatively high plastic strength at high temperatures [49, 50, 52]. These special characteristics make intermetallic compounds highly attractive candidates for various applications, for instance, as reinforcing additives in MMCs. This is mostly due to the ability of intermetallic phases to form a good interface and bonding with the matrix [53, 54]. However, the efficiency of this reinforcement strongly depends on factors such as the load transfer from matrix to intermetallic reinforcements, the type of additive, heat treatment, and the density of reinforcements.

**Fig. 2:**
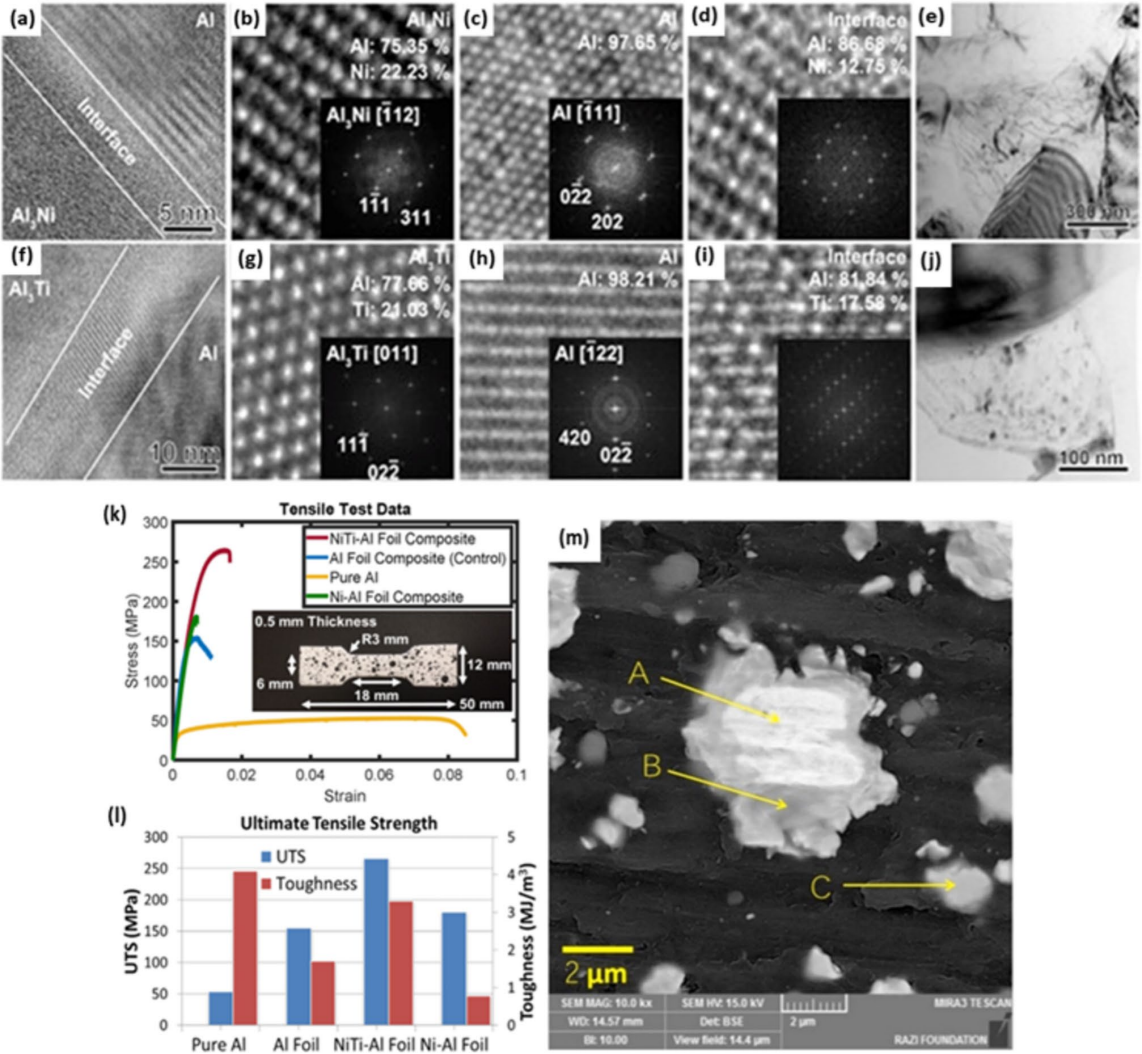
HRTEM/TEM images and diffraction patterns of the matrix (Al) and the two different reinforcing compounds (Al_3_Ni and Al_3_Ti). Atomic elemental percentages detected by energy-dispersive X-ray spectroscopy (EDS) are displayed: (**a**–**d**) the interface between the matrix and the Al_3_Ni particle, with the corresponding HRTEM images and FFT patterns of the Al_3_Ni particle, Al matrix, and the interface, respectively; (**e**) dislocations pile up near the boundary of the intermetallic particles; (**f**–**i**) the interface between the Al matrix and the Al_3_Ti particle, with the corresponding HRTEM images and FFT patterns of the Al_3_Ti particle, Al matrix, and interface, respectively; (**j**) low-energy dislocation structures are formed around the edges of the intermetallic particles [55] (reprinted with permission from WILEY–VCH, Copyright © 2018); (**k**) tensile test results and sample dimensions (strain rate of 1 mm/min) for three materials (NiTi-Al Foil, Al Foil, and Ni–Al Foil composites) in a strain hardened state (85% reduced by cold work); (**l**) comparison of UTS and toughness between pure Al, Al Foil, NiTi-Al Foil, and Ni–Al Foil [55] (reprinted with permission from WILEY–VCH, Copyright © 2018); (**m**) the SEM micrograph of in situ formed Al_3_Nb intermetallics and Nb/Al_3_Nb core-shells [57] (reprinted with permission from Elsevier B.V., Copyright © 2017)

The load-carrying capacity in intermetallics-reinforced MMCs hinges on the bond strength between the reinforcing intermetallic particles and the matrix, significantly influenced by the fabrication method. Heim et al. [55] employed the Rolling of Randomly Oriented Layer-wise Materials (RROLM) as the manufacturing methodology, achieving a layer-wise aligned distribution of micro-scale Al_3_Ni and Al_3_Ti intermetallics formed within a ductile Al matrix. Both strengthening and toughening mechanisms were observed in this specially designed particle reinforced composite due to the intrinsic crack deflection mechanisms achieved by creating layer-wise rectangular-like intermetallic particle reinforcements. Moreover, high resolution transmission electron microscopy (HRTEM) images and fast Fourier transform (FFT) patterns of Al_3_Ni, Al_3_Ti, and Al matrix indicated a clear distinct transition zone between the Al matrix and both Al_3_Ni (Fig. 2a–d) and Al_3_Ti (Fig. 2f–i) intermetallics. These transition zones can be responsible for the excellent cohesion and bonding strength between the intermetallic particles and the tough Al matrix, thereby improving load carrying capacity and preventing decohesion during cracking. Moreover, scanning electron microscope (SEM) images detected some thermally formed nano-sized Ni or Ti-based precipitates surrounding intermetallic particles in the Al matrix. These nano-sized particles not only pin dislocations (Fig. 2e), but also form low-energy dislocation structures with the size of several hundred nanometers along the particle boundary (Fig. 2j), improving the hardness around the intermetallic particles. In addition to hardness, ultimate tensile strength (UTS) of NiTi-Al foil (includes both Al_3_Ni and Al_3_Ti particles) enhanced to roughly 5.3 and 1.5 times higher than that of pure Al and Ni–Al foil samples, respectively (Fig. 2k, l). Although the toughness of NiTi-Al (3.3 MJ m^−3^) decreased with respect to the pure Al, it was much higher than that of Al foil (1.7 MJ m^−3^) and Ni–Al foil (0.78 MJ m^−3^) specimens. Therefore, by utilizing the RROLM process, it was possible to increase the UTS and toughness simultaneously [56].

Friction stir processing (FSP) is another fascinating solid-state process that leverages plastic deformation to fabricate in situ intermetallic/MMCs. During the FSP process, samples are exposed to highly localized low temperatures for a few seconds, which is highly favorable. By taking advantage of the exothermic reaction between Al and Nb particles during FSP, Al_3_Nb intermetallic particles could be formed in Al1050 matrix, as confirmed by the SEM micrograph in Fig. 2m [57]. The smaller size of Nb particles enhances the chance of atomic diffusion through the core, resulting in the complete transformation of Nb to Al/Nb particles (arrow C). Conversely, larger Nb particles prohibit complete transformation and instead contribute to core (Nb, arrow A)/shell (Nb/Al arrow B) particle formation. In addition, the number of FSP passes can have a positive impact on the mechanical properties of MMCs reinforced by intermetallic particles. More pass numbers result in a higher density of Al_3_Nb particles and their more uniform distribution, thereby enhancing composite’s hardness and strength. Specifically, four FSP passes were required to achieve a surface with evenly distributed Nb-Al_3_Nb particles and significantly improved mechanical properties, with hardness and UTS increasing by approximately 2 and 1.85 times, respectively) [58, 59].

Instead of its role in the in situ formation of particles, FSP also serves as a complementary post-fabrication method. FSP has been reported to be capable of modifying the structure, achieving a more uniform redistribution of Al_3_Ti and Al_3_Zr intermetallic particles, that were externally embedded in the AA6061 matrix during casting [60]. The segregation of needle-shaped Al_3_Zr particles was eliminated after FSP, transforming into a homogeneous distribution of spherical-shaped Al_3_Zr particles through fragmentation. Moreover, due to induced severe plastic strains by FSP, the Al_3_Ti clusters were crushed, facilitating a complete rearrangement of particles. This led to an enhanced microhardness, approximately 1.4 times that of the as-cast composite, due to the induced grain refinement, and reduced the wear rate of the composite by factors of 1.7 and 1.4 for FSPed AA6061/Al_3_Ti and AA6061/Al_3_Zr, respectively (see Table 1).

**Table 1:** Summary of the preparation methods and the obtained properties of MMCs reinforced with intermetallic particles

Matrix and additives	Fabrication method	Physico-mechanical properties	Remarks	Ref
Al/ (10% wt.%) NiTi nanoparticles	RROLM (160 MPa, 4 h, 630 °C)	UTS: 265 MPa Toughness: 3.3 MJ m^−3^	Formation of Al_3_Ni and Al_3_Ti intermetallics Possible to increase tensile strength and obtain an appropriate toughness simultaneously UTS of the sample reinforced with both Al_3_Ni and Al_3_Ti was around 5.3 and 1.5 times that of pure Al and Ni–Al foil samples	[55]
Al1050/ Nb powders	FSP (tool’s shoulder and pin diameter of 24 and 6 mm, tool penetration depth of 0.2 mm into the surface)	Hardness (4 pass number): 45 HV Tensile strength (4 pass number): 130 MPa	Formation of Al_3_Nb intermetallics Higher particle formation and a more uniform distribution of Al_3_Nb intermetallic particles by increasing FSP pass number Higher hardness (from around 20 HV for the as-received sample to more than 40 HV for the sample with 4 passes) and tensile strength (from around 70 MPa for the as-received sample to more than 120 MPa for the sample with 4 passes)	[57]
Al alloy (Al–5.6Zn–2.5 Mg–1.6Cu–0.23Cr (wt.%))/ (12 wt.%) Ti powder	SPS (580 °C, 30 MPa, 10 min, vacuum) followed by T6 process (466 °C/2 h + water-quenching + 120 °C/24 h) and hot rolling process (400 °C, room temperature, 20 rpm)	Tensile strength: Alloy–(SPS + hot rolling): 454 MPa Thickness of intermetallic shell after T6 process: 3.87 µm	Formation of core–shell structured (CSS) Ti–Al particles Further improvement of tensile strength caused by hot rolling and T6 heat treatment on SPSed specimens Enhancement of ductility and preventing microcrack nucleation resulted by thickening of intermetallic shell during T6 treatment	[61]
AA6061/Al_3_Ti AA6061/Al_3_Zr	In situ casting (addition of measured quantity of K_2_TiF_6_ or K_2_ZrF_6_ into the molten Al maintaining the temperature at 850 °C to form Al_3_Ti and Al_3_Zr, then stirring intermittently for 30 min)	AA6061/Al_3_Ti and AA6061/Al_3_Zr, respectively: Grain size: 5 µm, 3 µm Microhardness: 90 HV, 84 HV Wear rate: 250 × 10^−5^ mm^3^/m, 280 × 10^−5^ mm^3^/m	Refinement of the grain structure Improving microhardness Reducing wear rate Homogeneous redistribution of Al_3_Ti and Al_3_Zr intermetallic particles	[60]
Ti6242S (Ti–6Al–2Sn–4Zr–2Mo-0.1Si (m.%))/ (10 m.%) TNM powder (Ti–28.6Al–9Nb–2.3Mo–0.03B (m.%)	SPS (1100 and 1150 °C, 50 K/min, 50 MPa, then cooling via natural convection with 90 K/min)	Yield strength at different temperatures (for both 1100 and 1150 °C sintering temperature): 300 °C: 710 MPa 450 °C: 550 MPa 600 °C: 450 MPa Minimum creep rate: Ti6242S + TNM 1100 °C after 115 h: 2.0 × 10^−8^ Ti6242S + TNM 1150 °C after 170 h: 1.4 × 10	Formation of α_2_-Ti_3_Al and (Ti,Zr)_5_Si_3_ intermetallics Enhanced mechanical properties at elevated temperatures due to the presence of Si followed by the formation (Ti,Zr)_5_Si_3_ precipitates at the α/β lamellae boundaries and grain boundaries When sintered at 1100 °C, TNM particles still appear in the microstructure, while they were completely dissolved when sintered at 1150 °C generating a new alloy (Ti–8.3Al–1.8Sn–3.7Zr–2.0Mo–0.9Nb–0.08Si (m.%)) Prevention of dislocation glide and climb by ordered hexagonal α2-Ti_3_Al Increase in yield strength at all investigated temperatures due to the addition of TNM particles to the matrix Improvement of the creep resistance caused by the addition of TNM powder particles into the matrix (Ti6242S + TNM 1150 °C had the highest creep resistance)	[62]
Al/ (3, 5, 8, 10 wt.%) (Ni,Cu)_3_Al intermetallic particles	Low-energy mechanical milling (4 h, 930 MPa), sintering (2 h, 500 °C)	Yield stress (8%): 28 ± 3.5 MPa Young’s modulus (8%): 14.8 ± 0.38 GPa Percentage of elongation (8%): 5.1 ± 0.078 Microhardness (8%): 250 HV	Higher microhardness when increasing the particles’ wt.% from 3 to 8 (almost 2.5 times with respect to 3 wt.%) Decrease in the microhardness by further particle addition (10 wt.%) due to increase in material porosity	[63]
Al/ (20, 40, 60, and 80 vol.%) β-Al_3_Mg_2_ (complex intermetallic particles)	Hot pressing followed by hot extrusion (673 K, 500 MPa)	40 and 20 vol.% of β-Al3Mg2, respectively: Yield strength: 342 ± 15, 227 ± 10 MPa Fracture strain: 15.0 ± 1.1, 46.0 ± 3.5% Specific strength: 200, 180 kNm/kg	Superior yield and compressive strength compared to pure Al by a factor of 2–3, while preserving considerable plastic deformation (15 to 45%) for composites containing 20 and 40 vol.% of reinforcement particles Improved strength but negligible plastic deformation by further increasing to 60 and 80 vol.% of β-Al_3_Mg_2_ Decrease in the density of the material (compared to pure Al) and higher specific strength due to the low density of β-Al3Mg2 particles	[64]
Al/ (1, 3, 5 vol.%) Fe powder	CP (room temperature, 400 MPa) followed by further compression (200 MPa, 5 min) then sintering (5 h, 560, 570, 580 °C)	Density (3 vol.%): 2.50 g/cm^3^ (sintered at 580 °C) 2.56 g/cm^3^ (sintered at 570 °C) Compressive strength: 237.5 MPa (sintered at 570 and 580 °C)	Formation of Al_x_-Fe_y_ intermetallics Decrease in ultimate compressive stress (regardless of the sintering temperature) and ductility of the composite resulted by increasing Fe content Maximum density for composite sintered at 570 and 580 °C and Fe content of 3 vol.% Formation of a novel core–shell structure (which favors the ductility of the composite) for composites sintered at 560 °C Higher intermetallic fraction and lower core–shell structure as a result of increased sintering temperature	[65]
Mg/ combined addition of 5.6 wt.% Ti and 3 wt.% Cu (5.6Ti + 3Cu)	Rapid microwave sintering assisted powder metallurgy followed by hot extrusion (1 h, 200 rpm), CP (50 tons, 14 min)	(5.6Ti + 3Cu): Average grain size: 7 µm Microhardness: 69 ± 1 HV (50% higher with respect to pure Mg) Tensile strength: 253 ± 4 MPa (50% higher compared to pure Mg, and 12% higher compared to Mg–5.6Ti–3Cu) Ductility: 4.2 ± 0.6% 0.2YS: 223 ± 4 MPa (60% higher compared to pure Mg)	Formation of Ti_3_Cu and Mg_2_Cu intermetallics Grain refinement caused by the addition of micron-sized Ti and nano-sized Cu particles (5.6Ti + 3Cu) (combined addition of 5.6 wt.% Ti and 3 wt.% nano-Cu after pre-processing by ball-milling) had the lowest grain size, compared to pure Mg, Mg–5.6Ti, Mg–3Cu, Mg–5.6Ti–3Cu Obtaining the best mechanical properties for Mg-(5.6Ti + 3Cu) due to the effect of ball milling process that improved the morphology of Ti particles and promoted the formation of Ti_3_Cu	[66]
Al/Cu Al/Ti	FSP (no additional explanations were mentioned in the paper)	For Al-15Ti, and Al-15Cu respectively: Hardness: 226 ± 9, 160 ± 14 HV Yield strength: 654 ± 92, 441 ± 53 MPa	Formation of Al_2_Cu and Al_3_Ti intermetallics A dense microstructure with high strength properties due to the formation of intermetallic phases Increasing in strength with increasing reinforcement content	[67]

Despite the effectiveness of fabrication methods in enhancing mechanical properties of MMCs with intermetallics, post-treatment procedures also offer notable benefits. For instance, a new type of aluminum matrix composite (AMC), reinforced with Ti–Al intermetallic particles and subjected to spark plasma sintering (SPS), underwent hot rolling and T6 heat treatment (solution treating and artificial aging) [61]. The AMC processed with SPS followed by hot rolling exhibited the highest tensile strength (~ 454 MPa), which was 19% higher than that of the reference alloy tested under similar conditions. During T6 heat treatment, the intermetallic shell thickened from 2.30 to 3.87 µm. This thickening, in conjunction with the soft Ti-core, hindered micro-crack nucleation and thus improved the ductility of the composite.

**Fig. 3:**
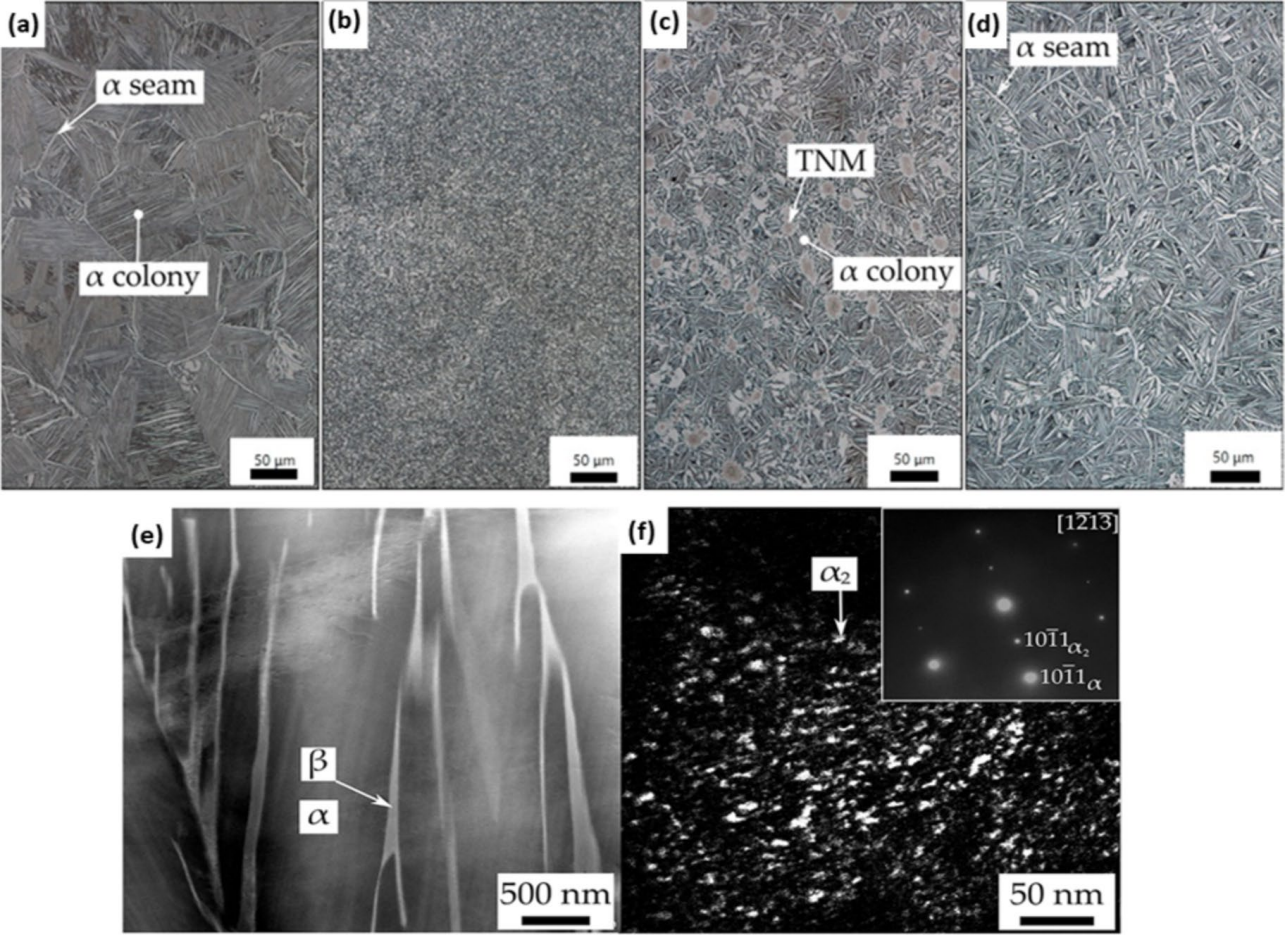
Light optical microscopy images of the Kroll etched specimens in as-SPS condition: (**a**) Ti6242S; (**b**) Ti6242 + 1 m.% B; (**c**) Ti6242S + TNM, dwell temperature 1100 °C and (**d**) Ti6242S + TNM, dwell temperature 1150 °C; (**e**) TEM investigation of the Ti6242S + TNM 1150 °C specimen in as-SPS condition. Dark-field scanning transmission electron microscopy image of α/β colonies, where the dark α lamellae are separated by bright retained β phase; (**f**) dark-field image of an α lamella. The insert shows the corresponding [1213] diffraction pattern of the selected area, which reveals the hexagonal structure as well as superlattice reflections. The 1011α2 superlattice reflection was used for the DF image. This 1011α2 DF image illuminates nanometer-sized ordered α2 domains separated by disordered α phase or antiphase boundaries, which appears in dark contrast [62] (reprinted with permission from Elsevier B.V., Copyright © 2020)

In an attempt to develop MMCs that retain their properties at high temperatures, the TNM alloy—a γ-TiAl-based intermetallic alloy widely utilized in the aerospace and automotive industries—was explored as a reinforcing additive. At high temperature, the microstructure is composed by three ordered phases: γ-TiAl (L1_0_-structure), α_2_-Ti_3_Al (D0_19_-structure), and β_o_-TiAl (B2 structure) [68]. To investigate the application of TNM alloy and formation of ordered α_2_-Ti_3_Al phases, Ti6242 alloy (Ti–6Al–2Sn–4Zr–2Mo, in wt.%) was reinforced with boron (B), and Ti6242S alloy (Ti–6Al–2Sn–4Zr–2Mo–0.1Si, in wt.%) was strengthened using particles of TNM alloy (Ti–28.6Al–9Nb–2.3Mo–0.03B, in wt.%) [62]. The β-eutectic element Si played an important role in Ti6242S composition since it caused the formation of stable intermetallic (Ti,Zr)_5_Si_3_ precipitates at the α/β lamellae boundaries and grain boundaries, which in turn enhanced the mechanical properties at elevated temperatures [69]. The addition of Sn increased the volume fraction of the hexagonal α_2_-Ti_3_Al intermetallic phase, serving as an effective barrier to dislocation glide and climb. Near-α Ti alloys exhibited a fully lamellar microstructure, with α lamellas interspersed with retained β phase, achieving the highest service temperatures [70, 71]. In this case, the final microstructure featured α colonies delineated by a continuous α layer (α seam) at the boundaries (Fig. 3a). A more refined microstructure was obtained by addition of B and TNM particles (see Fig. 3b and c). Due to the tendency of forming TiB intermetallics when adding B, a finer microstructure is observed in Fig. 3b compared to Fig. 3c. Based on Fig. 3d, by increasing the temperature up to 1150 °C, the TNM particles get dissolved leading to a larger grain size compared to Fig. 3c. Furthermore, the formation of ordered α2-Ti_3_Al intermetallic phase in the microstructure of Ti6242S and Ti6242S + 10 m.% TNM 1150 °C samples were analyzed using TEM. According to the diffraction patterns, addition of 10 wt.% TNM particles into the Ti6242S matrix transformed the disordered structure of α phase into an ordered α2 phase [72, 73]. Figure 3e presents a high angle annular dark field from scanning transmission electron microscopy image of an α/β colony in Ti6242S + 10 wt.% TNM 1150 °C sample. The insert in Fig. 3f shows the diffraction patterns of a selected area taken in [1213] zone axis, where the illuminated regions are the ordered domains of α2 phase. The whole grain is almost fully occupied by these ordered domains having a size of less than 10 nm.

The addition of TNM powder particles to the Ti6242S matrix has been reported to increase the yield strength compared to pure Ti6242S across all tested temperatures. Samples with B variant exhibited higher yield strength compared to those with TNM. However, a sharp drop in yield strength was observed above 550 °C due to boundary sliding. This sudden decline was not observed in the samples containing TNM powders. In addition, 20% yield strength improvement was reported in Ti6242S samples due to the addition of γ-TiAl-based alloy particles [62].

Recently, there has been a growing interest in core–shell structured (CSS) intermetallic particles, primarily due to their potential to enhance both strength and ductility simultaneously. This unique benefit stems from their composition: a stiff intermetallic shell surrounding a softer core. Studies have explored reinforcing AMCs with CSS intermetallic particles [61, 74–76]. These CSS reinforcements typically feature a hard, in situ–formed intermetallic shell with a soft Ti-core in the center [61, 74]. Compared to the large needle-like shaped Al_3_Ti particles fabricated by conventional techniques, these CSSs have the benefits of reducing the chance of cracking during plastic deformation and improving the ductility of the integrated composites [77]. A novel AMC reinforced by CSS Ti–Al intermetallic particles was developed using SPS at a relatively low temperature [78]. This composite, consisting of an 88% gas-atomized Al-alloy (Al–5.6Zn–2.5 Mg–1.6Cu–0.23Cr) and 12% Ti powder, showed significant improvements in both high tensile strength and elongation (up to 27%) compared to AMCs reinforced by single Al_3_Ti particles. The interfacial strains were also calculated to be minimal, suggesting that a coherent boundary was formed between the CSS reinforcements and the matrix. No cracks were formed along the interface or in the shell, indicating strong bonding at the interface. Tensile tests showed that under all conditions, the tensile strength of AMC was greatly improved through the formation of CSS particles. This can be attributed to Orowan–Bowing strengthening effect caused by intermetallic-shell with high specific-stiffness and grain-refining strengthening effect [79].

Finally, discovering the optimal quantity of intermetallic additive particles is crucial to achieving the desired mechanical properties. It was reported that addition of (Ni,Cu)_3_Al intermetallic particles into an AMC led to adverse effects when particles concentration exceeded 8 wt.% [63]. Microhardness enhanced from 26 to 254 HV as the intermetallic concentration rose from 3 wt.% up to 8 wt.%. However, the microhardness of the sample with 10-wt.% concentration experienced a drop to 230 HV, which could be attributed to the increase in material porosity [80, 81]. β-Al_3_Mg_2_ intermetallic particles were added into an Al-based matrix with different volume percentages of 20, 40, 60, and 80 vol.% [64]. The addition of low-density b-Al3Mg2 particles significantly improved the specific strength by lowering the overall density below that of pure Al. Moreover, composites reinforced with 20 and 40 vol.% of intermetallic particles exhibited yield and compressive strengths 2–3 times higher than those of pure Al, while maintaining a notable plastic deformation ranging from 45 to 15%. While increasing the β-Al3Mg2 phase to 60 and 80 vol.% could further enhance strength, the capacity for plastic deformation would be severely limited. Thus, selecting the optimal volume fraction of the additive demands careful consideration, tailored to the specific requirements of the target application.

### Bulk metallic glasses

Metallic glasses (MGs), recognized as an important member of advanced materials, were first identified in 1960 with the discovery of highly disordered atomic arrangements in solid state (Au_75_Si_25_) [40, 82]. Initially, the creation of metallic glass compounds required rapid cooling to prevent crystallization kinetically, necessitating a cooling rate between 10^5^ and 10^6^ K/s. This meant that the materials had to be extremely thin in at least one dimension to facilitate such rapid heat extraction, resulting in metallic glasses typically being formed into ribbons and wires [40, 41]. Thereafter, scientists looked for a BMG composition that could be obtained with bulk dimensions (at millimeter scale). The first bulk MG (BMG) was developed in the Pd–Cu–Si system in 1974, requiring critical cooling rate of 10^3^ K/s. A general rule to design BMG alloys involves selecting elements of varying sizes to form a complex structure that resist crystallization. Another guiding principle is to find alloy compositions with deep eutectics, to stabilize liquid state at low temperatures. Nonetheless, the knowledge behind the formation mechanism and the main effective factors on glass forming ability of alloys are yet not fully understood.

One specific characteristic of BMGs is the absence of dislocations, which considerably boosts their ultrahigh strength (up to 5 GPa), hardness, and wear resistance [83]. Moreover, BMGs exhibit excellent dimensional tolerance in casting process, enabling the production of complex shapes. Since crystallization is accompanied with volumetric shrinkage, there would be a low dimensional variation upon cooling of the BMG melts due to lack of crystallization [84]. BMG components can be constructed into complex shapes by reheating the as-cast product into the supercooled liquid region, allowing net-shape forming. The other advantage of BMGs is their considerable viscosity drop in the supercooled liquid region, contributing to achieve higher densities [38, 85, 86]. Moreover, BMGs possess exceptional elastic limit (2%), alongside superior corrosion and wear resistance, biocompatibility, and soft magnetism properties. These properties have recently attracted remarkable attention especially as the reinforcement phase of the MMCs [82].

**Fig. 4:**
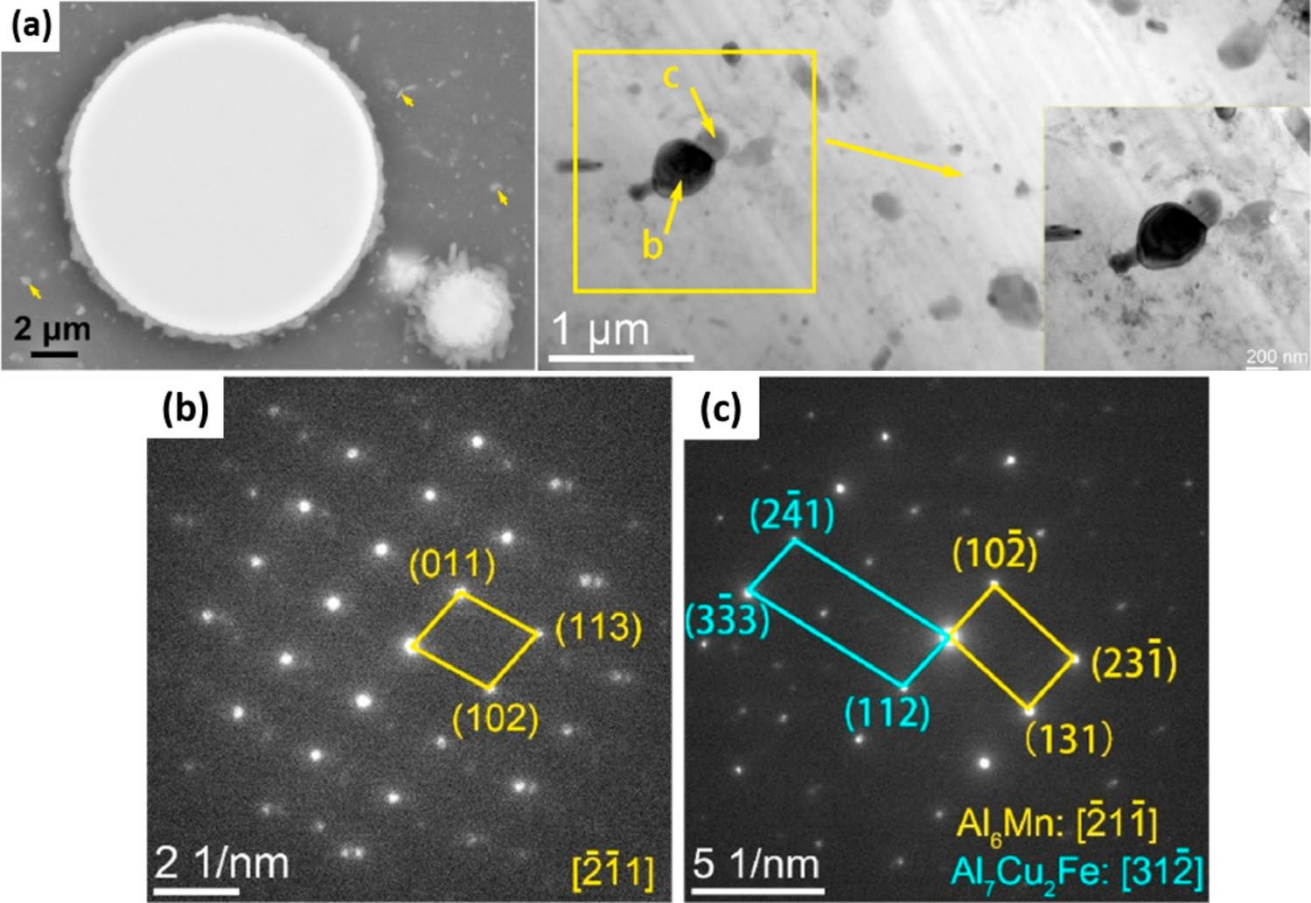
(**a**) Dispersoid (insoluble) particles in the Al2024 matrix; the inset shows the magnified micrograph of the rectangular area; (**b**, **c**) SAED patterns of the particles labeled b and c in (a), confirming the existence of Al_7_Cu_2_Fe and Al6Mn type phases, respectively [91] (reprinted with permission from Elsevier B.V., Copyright © 2020)

The performance of a composite is highly influenced by the interaction at interface between the matrix and the reinforcement. Numerous studies have focused on identifying the key parameters that affect these interactions, especially in composites reinforced with BMGs. However, the crystallization potential of MGs adds complexity to the reactions that occur at these interfaces. Therefore, it is critical to recognize the optimal conditions for material processing in these cases. For instance, Al7075 alloy reinforced with 30vol.% of Fe_50_Cr_25_Mo_9_B_13_C_3_ produced by the SPS technique and sintered at a temperature of 450℃ and a pressure of 30 MPa exhibited a 34% increase in microhardness (from 119 to 160 HV) attributed to the excellent interfacial bonding without the formation of any new phases or layered structures [87]. In contrast, a composite with 20 vol.% of Fe_66_Cr_10_Nb_5_B_19_, sintered at 570℃, reached an optimized hardness of 280 HV, indicating the importance of precise control over each process step. In this case, sintering at 540 ℃ was insufficient as the hardness of Al matrix only reached 75 HV. Moreover, the sample sintered at 570 ℃ displayed an interfacial layer that was 10–15-µm thick around the Fe-based cores. The formation of reaction layers can be considered a positive structural feature, as it facilitates load transfer from the soft and weak matrix to the hard shell and core. Excitingly, when the sample was prepared by sintering at 500 ℃ followed by annealing at 570 ℃, a thinner interface layer was observed [88]. The thicker interfacial layer observed in the sample sintered at a higher temperature without annealing can be attributed to the occurrence of higher local overheating at the interface during SPS. Therefore, it is important to consider interfacial resistance as a significant factor in the phase formation at the interface of MMCs, as it can lead to overheating at the interface [89, 90]. In a detailed research, He and colleagues realized Al/Fe_43.2_Co_28.8_B_19.2_Si_4.8_Nb_4_ composite and investigated the role of the effective parameters on the interfacial product [91]. It was shown that the interface characteristics can be tailored by manipulating the heat treatment temperature, soaking time, and particle size. For instance, by increasing the soaking temperature and time from 480 ℃ and 10 min to 490 ℃ and 25 min, the thickness of interface layer increased from 224 ± 50 nm to 353 ± 45 nm when the median particle size (D_50_) was 16 µm. By using particles with *D*_50_ = 72 µm in the previous heat treatment, the thickness of the interface enhanced from 227 ± 36 nm to 316 ± 56 nm, i.e., the interfacial layer became thinner. The EDX and XRD results demonstrated that the interfacial product is an intermetallic compound of Al_7_Cu_2_Fe. The XRD patterns revealed the presence of minor peaks that were attributed to an insoluble compound of Al_6_Mn, which was also observed in the SEM images (indicated by arrows). Furthermore, the existence of this compound was confirmed through selected-area electron diffraction (SAED) analysis (Fig. 4a–c).

**Fig. 5:**
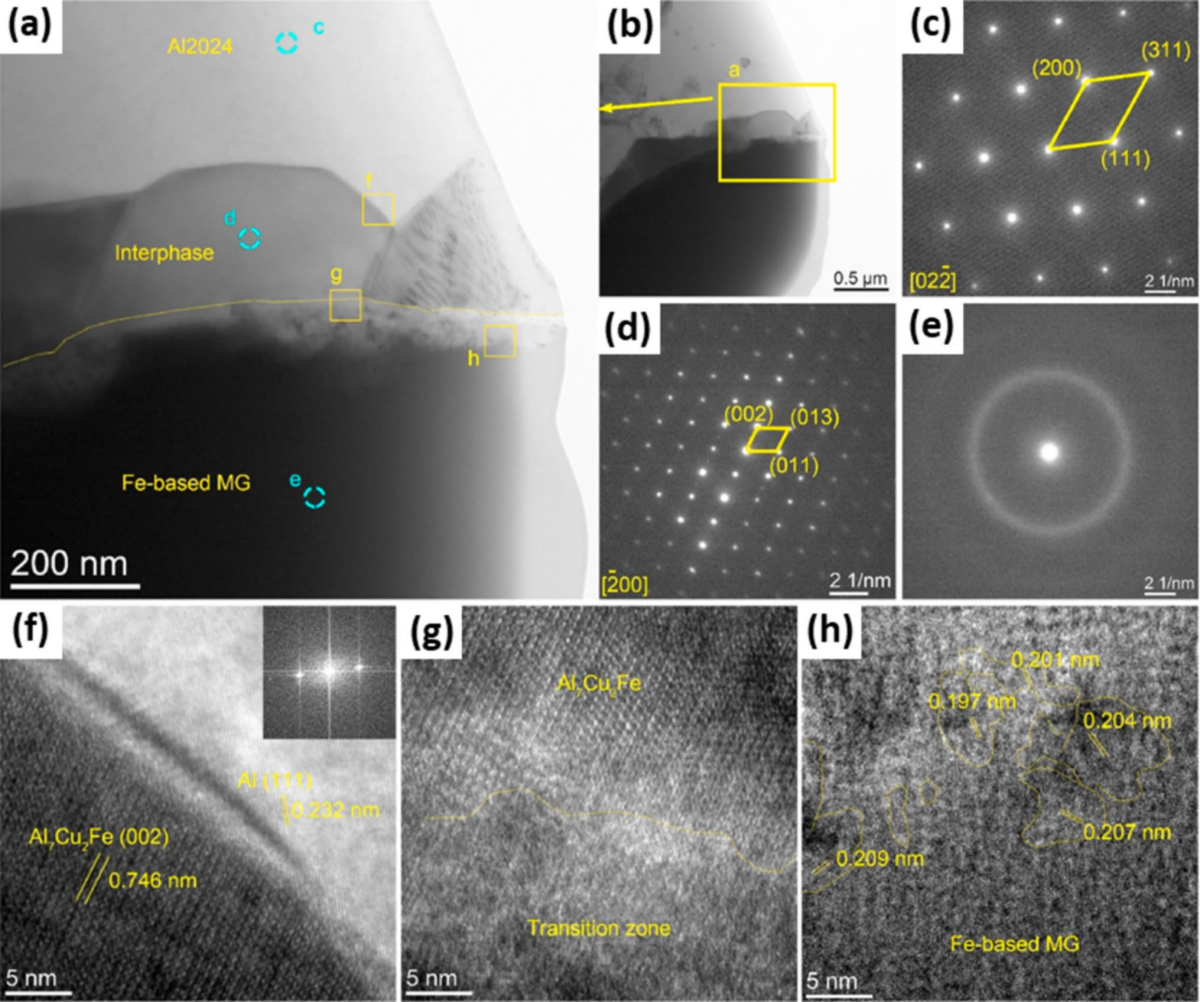
TEM images of Al2024/Fe-based MG with *D*_50_ = 16 μm after 763 K/25 min heat treatment. (**a**, **b**) Morphology of the interface between Al2024 matrix and Fe-based MG, where (a) is the magnified image of the rectangular area in (b); (**c**–**e**) SAED patterns of Al2024 matrix, interphase and glassy particle, confirming the FCC-Al structure, formation of Al_7_Cu_2_Fe phase and amorphous structure of the MG, respectively [91] (reprinted with permission from Elsevier B.V., Copyright © 2020). (**f**) HRTEM image of the Al/Al 7 Cu 2 Fe interface, corresponding to the zone f in (a); the inset shows the FFT of the FCC-Al; (**g**, **h**) HRTEM observation of the zone between Al 7 Cu 2 Fe phase and metallic glass, corresponding to the rectangular areas g and h in (a), respectively, showing the existence of a transition layer between these two phases and the formation of nanocrystals in the transition zone [ 91 ] (reprinted with permission from Elsevier B.V., Copyright © 2020)© 2020).

FCC α-Al, Al_7_Cu_2_Fe intermetallic compound, and BMG reinforcement can be recognized in the HRTEM images of interface of the composite and SAED pattern (Fig. 5a–e). Moreover, a well chemical bonding can be observed between the matrix and intermetallic compound, while a transition region that bonded the intermetallic phase to MG (shown by yellow dotted line in Fig. 5a, f, and g) can be identified [91]. One important observation in these images was the maze-like nanostructure (Fig. 5h), characteristic of the amorphous phase, in the lower region of the transition zone with interplanar distance of about 2 nm and size of 3–5 nm. Thermodynamically, Cu–Fe and Al–Fe are both immiscible; however, there is a high chance of forming intermetallic compounds such as Al_3_Fe and AlFe_3_ in the Al–Fe system due to the high affinity between Al and Fe. On the other hand, during the solid solution treatment of Al2024, Al_2_Cu and Al_2_CuMg soluble phases might be dissolved into the α-Al lattice. When Fe-based MG is present, there would be a chance of diffusion of the atoms through the interface at high enough temperatures. As the diffusion coefficient of Al is higher than that of Fe, it is expected that more Al atoms diffuse toward the amorphous phase, resulting in the formation of Al–Fe intermetallic compounds. In this way, the presence of Al_13_Fe_4_ nanocrystals in zone h of the composite (Fig. 5h) can be described. As the interface is a swift diffusion pathway, Cu atoms might react with Al_13_Fe_4_ to form the Al_7_Cu_2_Fe intermetallic compound [91]. Moreover, decreasing the interface thickness by increasing the particle size would be described by gradually increasing the diffusion path after the formation of intermetallic layer. Indeed, before the existence of the intermetallic product, there was no obstacle against the diffusion of atoms across the interface [91].

**Fig. 6:**
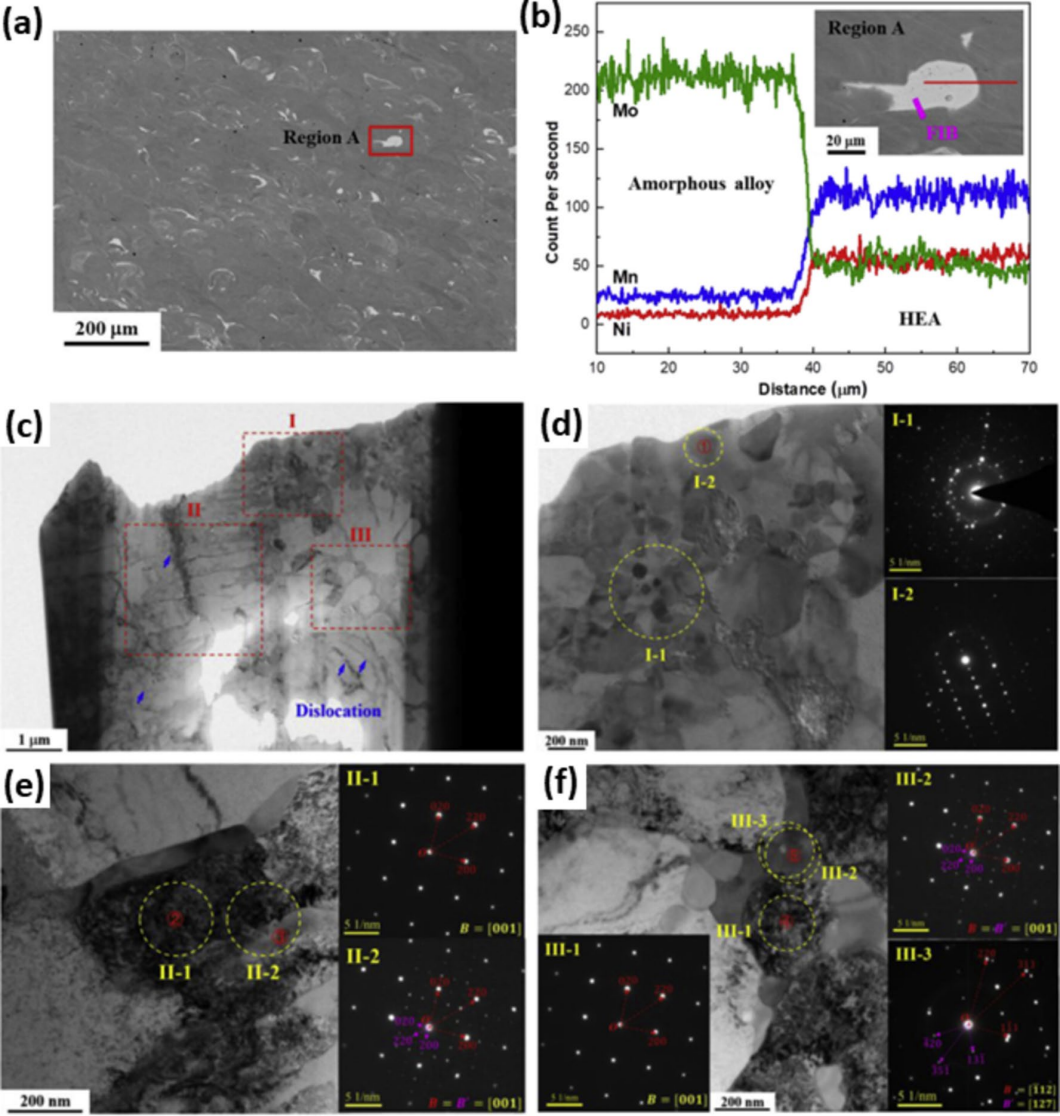
(**a**) SEM micrographs represent random distribution of MG (20 wt.%) in HEA. (**b**) The line scanning as shown in the inset reveals possible interfacial transition layer. (**c**) The TEM bright-field micrograph of the interfacial zone as marked in the inset of (b), which is divided into three regions (I, II, III); (**d**) bright-field micrograph of region I, zone I-1 reveals multi-crystal feature, zone I-2 shows nanocrystals FeMo_2_ phase; (**e**) bright-field micrograph of region II, zone II-1 shows FCC crystal, while the diffraction pattern in zone II-2 reveals two different crystals; (**f**) bright-field micrograph of region III, which further demonstrates the two different crystals [97] (reprinted with permission from Elsevier B.V., Copyright © 2020)

In addition to optimizing fabrication parameters, it is also essential to investigate the optimal amount of reinforcement phase. For instance, when Al7056 was reinforced by Zr–Al–Ni–Cu, it was found that increasing the Al alloy content in BMG from 10 to 30 vol.% reduced the mechanical properties, particularly strength [92]. Moreover, the selection of the appropriate reinforcement composition for the desired application is of utmost importance. HEAs as newly found metallic materials have captivated researchers due to their outstanding properties, which will be discussed in detail in the following sections. Face-centered cubic structures in HEAs (e.g., FeCoCrNiMn alloy) are attractive owing to their supreme plasticity and fracture toughness in cryogenic conditions [93, 94]. However, their low yield strengths are a main drawback. Ceramic nanoparticles manifested great power in enhancing the yield strength of FeCoCrNiMn significantly, but their plasticity reduced [95, 96]. Li et al. [97] showed that employing Fe-based MG of Fe_43.7_Co_7.3_Cr_14.7_Mo_12.6_C_15.5_B_4.3_Y_1.9_ (at.%) as the reinforcement phase can address the limitations of FeCoCrNiMn through such complex interactions in the atomic scale. Accordingly, the composite fabricated by an additive manufacturing technique named laser powder bed fusion (L-PBF) resulted in a crack-free and homogenous microstructure with a uniform distribution of the reinforcement, as illustrated in Fig. 6c and d. In TEM images of the interfaces, three main regions were recognized, namely, I (polycrystalline region), II (strip region), and III (carpet-like region) (Fig. 6a–d). Region I constituted FeMo_2_, which indicates that a part of MG was crystalized since Mo was only present in the MG composition. The Fe-based utilized MG has a high glass-forming ability; however, the element diffusion between the HEA matrix and MG caused a composition deviation. Evaluation of the strip structure also confirmed the composition deviation, and thus, zone II-1 was identified as the HEA matrix while zone II-2 represented the original amorphous phase which was crystallized into a new HEA (Fig. 6e). Importantly, the inset II-1 demonstrates that the FCC structure of the matrix was preserved, and as a result of diffusion, the amorphous phase evolved into another FCC structure, as shown in spot 5 of region III. The carpet-like area also constituted HEA in the center and an amorphous phase in the surroundings [97]. This excellent continuous interface led to a high mechanical performance.

**Fig. 7:**
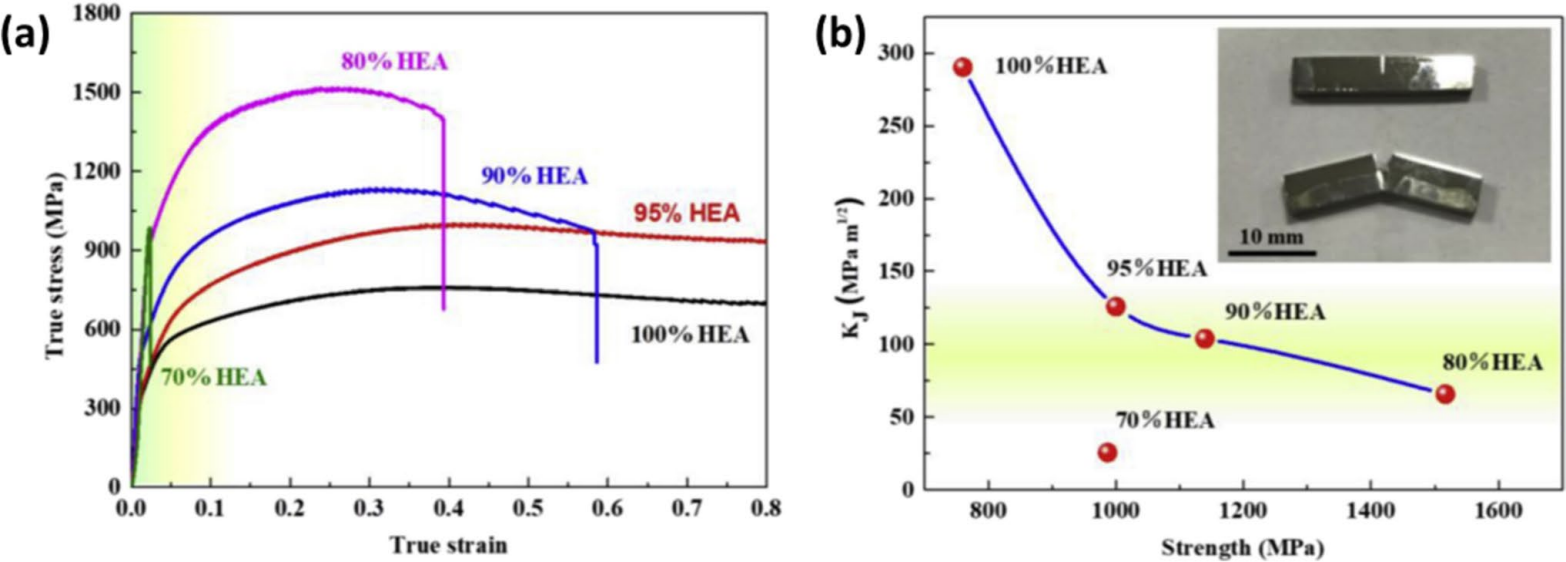
(**a**) True stress–strain curves of HEA composed of gradient fraction of the Fe-based MG (0–30%). (**b**) Fracture toughness vs. strength curve of the HEA and MG composite, the insert image depicts a fractural sample (HEA-20%MG) compared with the original one [97] (reprinted with permission from Elsevier B.V., Copyright © 2020)

As depicted in Fig. 7a, by adding 0–20 wt.% Fe-based MG to HEA matrix, the yield strength increased from 315 to 916 MPa (more than 190% increment); however, the plastic strain reduced from more than 0.8 to 0.39 [97]. Further amount of MG caused brittle fracture since additional MG particles led to microcrack formation during the L-PBF process, which is also reported elsewhere [98]. In addition, according to Fig. 7b, incorporating more MG particles into the HEA matrix enhanced the strength while fracture toughness dramatically decreased. Nonetheless, for HEA/20 wt.% MG, the strength and fracture toughness of 1517 MPa and 65.67 MPa.m^1/2^ were obtained, respectively, which is in the acceptable range for many engineering applications [99].

Another parameter that requires to be taken into account to select an appropriate BMG reinforcement is the crystallization temperature of the reinforcement. Indeed, this is a limiting parameter in the MG family since they are in a non-equilibrium thermodynamic state and tend to be crystallized by heat treatment. Ni-based MGs suggest high crystallization temperatures that turn them into ideal candidates as the reinforcement of the heat-treatable metals [14, 100], especially aluminum matrix in which crystallization can be achieved after heat treating for a long time at temperatures higher than 580 ℃ [33, 35]. Ertugrul and coworkers examined how the heat treatment will affect the microstructure and mechanical behavior of Al2024/Ni_60_Nb_40_ [101]. The well-mixed powders were consolidated by hot pressing (HP) at 400 ℃ at a pressure of 640 MPa with a holding time of 10 min. Thereafter, the heat treatment process was carried out with solutionizing at 500 ℃ for 1 h, followed by rapid cooling in water, and finally an aging step at 150 ℃ for 18 h. The XRD patterns showed the presence of α-Al as the main phase, CuAl_2_ and Al_2_CuMg intermetallic phases, and a broad peak attributed to the MG phase. After the heat treatment, Al_2_CuMg almost vanished and new phases of CuNiAl and NbNiAl emerged as a result of interfacial reactions. The SEM images clearly illustrate that the interface was intact before heat treatment, while in the heat-treated composites, a thin surrounding layer (2–3 µm for 20 vol.% and 2–10 µm for 40 vol.% Ni_60_Nb_40_ samples) was observed around the reinforcements. Stress–strain diagrams obtained from compression tests indicated the notable role of heat treatment in enhancing the yield strength of all samples (e.g., about 41% enhancement from 229 to 323 MPa for Al2024/20vol.%Ni_60_Nb_40_). Importantly, the addition of 20 vol.% Ni_60_Nb_40_ did not place any adverse implications on its plasticity up to 20% strain. In a similar work, Li et al. [102] employed Ni-based MG of Ni_59_Zr_20_Ti_16_Si_2_Sn_3_ (at.%) (with crystallization temperature of 602 ℃) to strengthen the heat-treatable Al–Zn–Mg–Cu alloy. The addition of 20 vol.% of Ni_59_Zr_20_Ti_16_Si_2_Sn_3_ without heat-treating process was sufficient to enhance the yield strength from 263 to 401 MPa. By heat treatment, the yield strength improved significantly from 494 to 728 MPa, which was much higher than the reported value for heat-treated Al2024/20 vol.% Ni_60_Nb_40_ [96]; this observation indicated the exceptional response of the Ni-based MGs to heat treatment that can be attributed to the excellent interface bonding along with the formation of various phases including Al_3_NiCu and complex phases of Al–Zn–Mg–Cu. Despite adding 20 vol.% of Ni_59_Zr_20_Ti_16_Si_2_Sn_3_, the plasticity of the composite was found acceptable with a fracture strain of 17% [102]. Thus, exploiting Ni-based MG particles as the reinforcement for Al matrices would reward high mechanical strength and plasticity simultaneously, which might be considered an evolution in advanced materials industries.

**Fig. 8:**
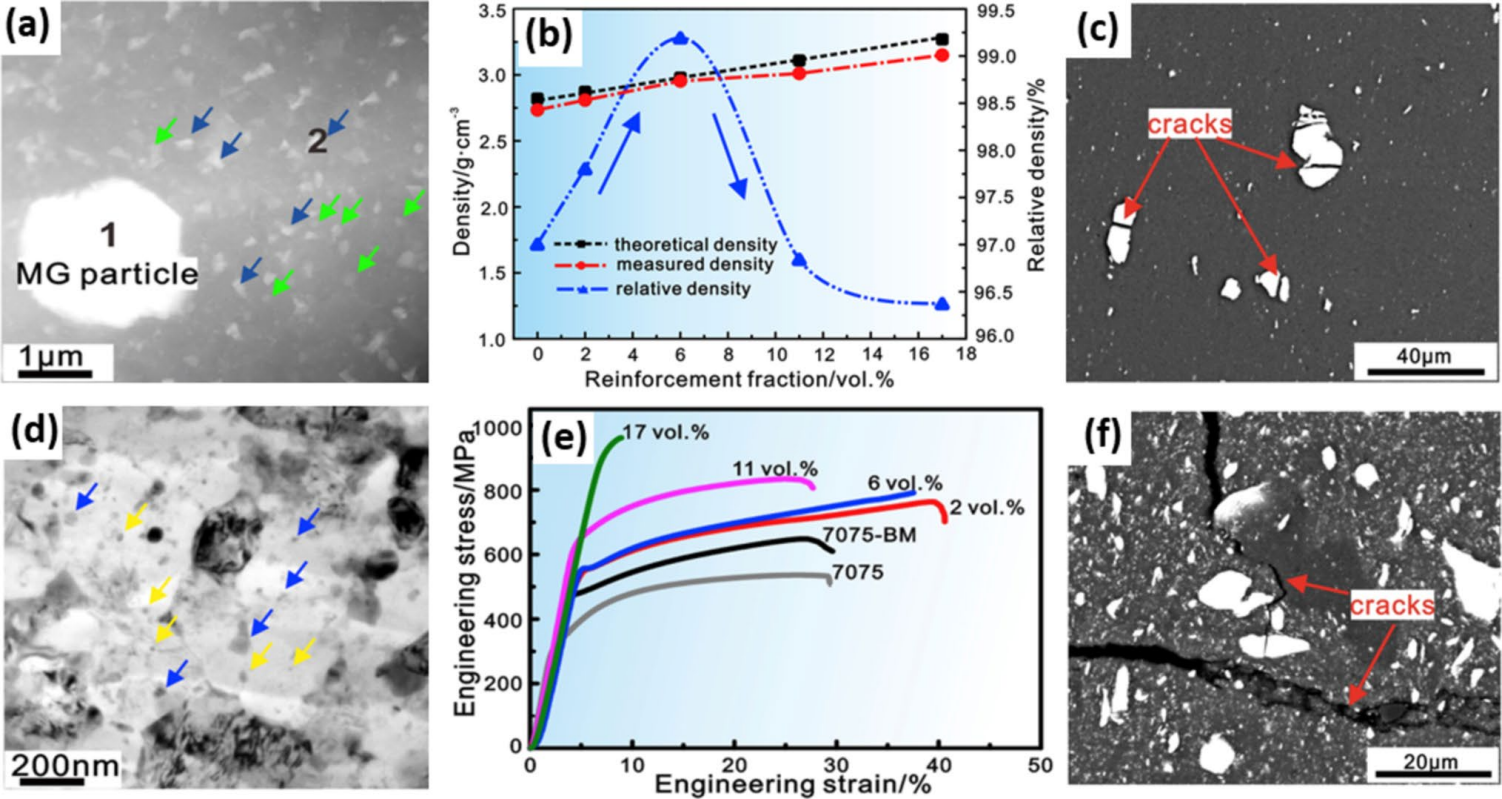
(**a**) Dark-field and (d) bright-field TEM micrographs of Al7075 reinforced with 6 vol.% Ti_55.5_Cu_18.5_Ni_17.5_Al_8.5_ particles. (**b**) Density and relative density of the composites. (e) Room-temperature compressive stress–strain curves of the Al7075 reinforced with different volume fractions of Ti_55.5_Cu_18.5_Ni_17.5_Al_8.5_ particles. (**c**, **d**) The SEM micrographs of the lateral surface for the composite with 2 vol.% and 17 vol.% reinforcements after compressive test, respectively [105] (reprinted with permission from Elsevier B.V., Copyright © 2020)

Tailoring the physical characteristics of reinforcement is another effective way to control the performance of the reinforced MMC. MGs are mostly utilized in micron size, which can benefit the MMC through direct strengthening effect owing to load bearing capacity and indirect strengthening effect from the matrix. However, MMCs with micron-size reinforcement are highly prone to crack initiation and swift propagation, assisting also by a dramatic deterioration of the ductility. One promising solution is to take advantage of the bimodal size particle reinforcement since the incorporation of nanoparticles contributes to Orowan strengthening mechanism by providing barriers against dislocations, and consequently promoting Orowan loops around the reinforcements [103, 104]. Recently, bimodal Ti-based (Ti_55.5_Cu_18.5_Ni_17.5_Al_8.5_) MG particles were exploited to reinforce Al7075 matrix [105]. By taking advantage of a well-designed procedure composed of ball milling, cold pressing, and hot extrusion, the initial micron-sized (< 38 µm) MG particles blended with Al7075 powder were broken into nano-sized particles as demonstrated by blue arrows in Fig. 8a. For instance, in Al7075/6 vol.% Ti_55.5_Cu_18.5_Ni_17.5_Al_8.5_ as a composition with the best performance, 1.7 vol.% of the reinforcements was in the range of nanometer. Nanosized MgZn_2_ precipitates were also formed in the matrix as shown by green arrows in Fig. 8d. In addition, ultrafine reinforcements assisted in reducing the average grain size from 3 µm to 250 nm by pinning the grains, hindering or decelerating the grain growth.

A low amount of MG particulates of Ti_55.5_Cu_18.5_Ni_17.5_Al_8.5_ up to 6 vol.% were reported to promote the Al-based composite densifications, as shown in Fig. 8b, while using ceramic particles usually causes a lowering the densities. Indeed, during the hot extrusion process, MG particles are in the supercooled liquid state; thus, they can readily fill the pores and cracks by the applied shear deformation. By optimizing the sample preparation procedure, the reinforcement particles were well dispersed in the matrix and no agglomeration and voids were observed. However, utilizing higher amounts of the reinforcement up to 17 vol.% led to MG particle agglomeration and pore formation. During the hot extrusion phase, shear stresses are applied, which are capable of moving large particles, sticking them to the neighboring ones, and thus resulting in clustering. In addition, fracture and interfacial debonding can be a consequence of the large stress concentration around the coarse particles.

It is interesting to note that introducing up to 6 vol.% bimodal reinforcement to the matrix enhanced the fracture strain in comparison with pure Al7075, as depicted in Fig. 8e, highlighting a better densification. Utilizing higher contents of the Ti_55.5_Cu_18.5_Ni_17.5_Al_8.5_ was found effective in increasing the yield strength and ultimate compressive strength at the expense of diminishing the ductility. Fractography analysis indicated that the microparticles were more prone to be fractured than the nano particles (Fig. 8c), due to the higher local stress concentration around the larger particles paired with the higher probability of intrinsic imperfections due to their larger size. Interestingly, the micrographs showed that the generated cracks in the microparticles failed to propagate along the interface, attributed to the excellent chemical bonding at the interface (Fig. 8f).

In addition to incorporating bimodal-sized Ti-based MG reinforcement, nanoscale Ti-based MG particles (8 vol.% Ti_52_Cu_20_Ni_17_Al_11_) were utilized to reinforce an Al-7075 matrix [106]. The composite powder was prepared by ball milling micron-sized (20–50 μm) MG particles with Al alloy powder for an extended duration of 10–50 h. The harsh milling conditions resulted in the majority of the MG particles being crushed into nanoscale particles, especially after 30 h of milling. The composite powder was then compacted through the cold pressing (CP) method, followed by hot extrusion at 400 ℃ under a pressure of 590 MPa. Microstructural analysis of the samples after compressive deformation indicated the formation of a 4–5-nm interdiffusion layer at the interface, with no signs of debonding. The 30-h milled sample exhibited a uniform dispersion of ultra-refined nano-MG particles, which resisted active dislocation movement due to the large stress field around them. The yield strength of the composites remarkably increased with increasing the milling time, from 297 MPa for the Al-7075 matrix to 530, 880, and 1014 MPa for the composites milled for 10, 30, and 50 h, respectively. However, the plasticity of the samples decreases significantly with an increase in milling time, from 27% for the monolithic Al alloy to almost 0% for the sample milled for 50 h. The high density of dislocations accumulated during milling and severe plastic deformation during short-time hot extrusion may have contributed to this trend [106]. Further research is necessary to overcome this bottleneck toward leveraging the benefits of nanoscale MG particles while ensuring that plasticity is not adversely affected. In Table 2, additional information is provided regarding the preparation methods and the physico-mechanical properties of MMCs reinforced with BMG and SMA particles.

**Table 2:** Summary of the preparation methods and the obtained properties of MMCs reinforced with BMG and shape memory alloy particles

Composite	Fabrication method and condition	Physico-mechanical properties	Remarks	Ref
Al/ Ni_50_Ti_50_ (20 wt.%)	CP (10 MPa) Microwave sintering (550 °C, 30 min)	Microhardness: 116 HV Compressive yield strength: 134 MPa Compressive fracture strength: 589 MPa	Good interfacial bonding Homogeneous distribution of amorphous particles with small porosity at some locations	[24]
Al2024/ Fe_73_Nb_5_Ge_2_P_10_C_6_B_4_ (15 wt.%)	HP (550 °C, 400 MPa, 30 min) Hot extrusion (extrusion ratio of 10:1)	Compressive yield strength: 403 MPa Compressive fracture strength: 660 MPa Fracture strain: 12%	Nanostructured Al-2024 matrix with a grain size of about 30 nm was obtained after 24 h of milling together with a good distribution of the Fe-based metallic glass particles in the Al matrix. Good dispersion of reinforcement particles	[29]
Al/ Mg_65_Cu_20_Zn_5_Y_10_ (10%)	HP (180 °C, 700 MPa, under vacuum)	Yield strength: 203 MPa compressive strength: 247 MPa Plastic strain: 25%	The uniform distribution of the glassy particles and the good reinforcement–matrix interface led to significant improvement of the room-temperature compressive behavior. Increasing the yield strength more than 3 times combined by an appreciable plastic deformation 25%	[36]
Al7075/ Fe_50_Cr_25_Mo_9_B_13_C_3_ (30%)	SPS (450 °C, 10 min, 30 MPa) Heat treatment (450 °C, 1 h) Hot extruded (extrusion ratio of 22:1)	Hardness: 160.63 HV Ultimate compression strength: 749 MPa Fracture strain: > 20%	Due to low-temperature rapid SPS consolidation, there were no new phases or layer structure at the interface because of the limited diffusion between the two phases. Excellent interface without any defects	[87]
Al/ Fe_66_Cr_10_Nb_5_B_19_	SPS (540–570 °C, 3 min, 40 MPa)	Relative density: 97% (540 °C) and 99% (570 °C) Hardness: 75 HV (540 °C) and 280 HV (570 °C)	Increasing T caused more interaction and better element immigration from particle to the outside and higher hardness	[88]
Al2024/ Fe_43.2_Co_28.8_B_19.2_Si_4.8_Nb_4_	HP (450 °C, 10 min, 640 MPa, under argon)	Yield strength: 349 MPa Fracture strength: 482 MPa Fracture strain: 5.3% reducing is higher for 16 µm (yield strength 25 MPa)	By increasing both the particle reinforcement size (from 16 to 72 µm) and sintering time (from 10 to 25 min), the mechanical performance diminished owing to the formation of Al_7_Cu_2_Fe intermetallic phase. This reduced the availability of Cu for precipitation hardening, leading to decreased composite strength by weakening the particle–matrix interface	[91]
Fe_20_Co_20_Cr_20_Ni_20_Mn_20_/ Fe_43.7_Co_7.3_Cr_14.7_Mo_12.6_C_15.5_B_4.3_Y_1.9_	SLM (laser power *P* = 185 W, laser scan speed *V* = 600 mm/s, powder layer thickness h = 40 mm, scan line hatch spacing t = 0.1 mm, scanning direction of 90° alternately among layers)	Yield strength: 916 MPa (5 wt.% reinforcement) and 1517 (20 wt.% reinforcement) Toughness: 65.67 MPa.m^1/2^ (5wt.% reinforcement) and 126 MPa m^1/2^ (20wt.% reinforcement)	Reservation of original HEA matrix during the high-energy laser processing due to its excellent thermal stability. The addition of the MG to the HEA enhanced the ductility and fracture toughness of the material, while maintaining its high strength	[97]
Al2024/ Ni_60_Nb_40_	HP (400 °C, 10 min, 640 MPa, under argon) Heat treatment (T6) (solutionizing at 500 °C for 1 h, water quenching and an aging step at 150 °C for 18 h)	Yield strength: 323 MPa (20 vol.% reinforcement) and 389 MPa (40 vol.% reinforcement) True strain: > 20% (20 vol.% reinforcement) and 7.6% (40 vol.% reinforcement)	The addition of 20 vol.% metallic glass had no adverse effect on the plastic deformability in the strain range studied. T6 heat treatment provided an interface layer between the matrix and the reinforcement and thus increased the mechanical strength	[101]
Al_10.78_Zn_2.45_Mg_1.70_Cu_0.17_Zr (wt.%)/ Ni_59_Zr_20_Ti_16_Si_2_Sn_3_ (20 vol.%)	HP Hot extrusion (400 °C, 500 MPa, extrusion ratio of 25:4) T6 heat treatment (solution treatment at 475 °C for 1 h and 485 °C for 1 h, water quenching, artificial aging at 120 °C for 18 h)	Yield strength: 728 MPa Ultimate strength: 890 MPa Fracture strain: 17.1%	High crystallization temperature (602 °C), which allows heat treating the composite while avoiding crystallization of the reinforcement At the matrix/particle interface, a layer was formed after heat treatment of the extruded materials	[102]
Al7075/ Ti_55.5_ Cu_18.5_Ni_17.5_Al_8.5_ (6 vol.%)	CP Hot extrusion (400 °C, 590 MPa)	Relative density: 99.2% Yield strength: > 500 MPa Fracture strength: < 800 MPa Fracture strain: 37%	The relative density, ductility and strength increased using up to 6 vol.% reinforcement. The micro-reinforcement was beneficial to the load transfer effect, the nano-reinforcement was mainly favorable to the grain refinement and Orowan strengthening	[105]
Al7075/ Ti_52_Cu_20_Ni_17_Al_11_ (8 vol.%)	CP Hot extrusion (400 °C, extrusion ration of 11:4, 590 MPa, 1 min)	Relative density: 97.3% Yield strength: 1014 MPa	Particles that remained in µm size broke down into submicron or nanoparticles as the time for ball milling increased Yield strength increased 3.4 times with increasing milling time up to 1014 MPa for 50 h	[106]
Al/ Al_84_Gd_6_Ni_7_Co_3_ (20%)	HP (200 °C) Hot extrusion (450 °C, extrusion ratio of 6:1)	Yield strength: 93 MPa Tensile strength: 157 MPa Fracture strain: 5%	No particle–matrix debonding	[107]
Mg/ Ni_50_Ti_50_ (10 vol.%)	CP (500 MPa) followed by microwave sintering (power of 900 W, duration of 11 min). The sintered billets were coated with colloidal graphite and soaked at 400 °C for 1 h and hot extruded at 400 °C	Microhardness: 66 HV Compressive yield strength: 102 MPa Compressive failure strain: 14.9% Tensile yield strength: 148 MPa Tensile failure strain: 2%	Significant grain refinement by about 60% was achieved in case of 6 vol.% Ni_50_Ti_50_ addition. Under compressive loads, the incorporation of Ni_50_Ti_50_ amorphous particles significantly enhanced the strength without notable adverse effects on ductility	[108]
Al/ Al_65_Cu_20_Ti_15_ (10 vol.%)	ECAP (preheated at 250 °C)	Yield strength: 251 MPa (after three passes) Fracture strain: 51%	A homogenous ultrafine grained structure with average grain size of 610 nm was developed after four passes of ECAP	[109]
Al6061/NiTip (10 vol.%)	FSP Water quenching after treatment at 515 °C for 40 min Aging at 163 °C for 18 h	UTS: 350 MPa YS: 304 MPa EL: 9.1%	Lowering the time of exposure to high temperatures, so no intermediate phases were found at the interface	[110]
Mg_59.5_Cu_22.9_Gd_11_Ag_6.6_/NiTi (20 vol.%)	Melting at 1273 K for 3 min Copper mold casting	Plastic strain: 10.6% Fracture strain: 12.1% Fracture strength: 1173 MPa	Improvement of tensile ductility of BMG composites	[111]
Mg67Zn29Ca4/porous NiTi (3 vol.%)	Ingot preparation: introducing the NiTi particles into the Mg–Zn–Ca melt at 1073 K under mechanical stirring Final samples: remelting the ingots followed by injection into a copper mold	Fracture stress: ~ 592 MPa	Enhancement of corrosion resistance in Hank’s solution	[112]
Cu51.5Zr48.5/CuZr (6 vol.%)	Cylindrical rod preparation by suction casting arc-melter of the molten pure Zr and Cu Cooling rate (rod surface): 5000 K/s Cooling rate (rod axis): 220 K/s	Yield strength: 1424 MPa Maximum strength: 1935 MPa Fracture strain: 9.5%	The highest ductility was obtained from the sample which embedded the reinforcements with a volume fraction of ~ 30–40%	[113]

### Shape memory alloys

SMAs have gained considerable attention owing to the ability to remember their original shape and return to it after deformation, known as the shape memory effect (SME) [114–116]. This class of advanced materials encompasses various alloys, such as Cu-based, Fe-based, Co-based, Au-Cd-based, and Nitinol (nickel–titanium) [117]. SME refers to the remarkable ability of SMAs to retain their original shape after experiencing thermal or stress-based deformation [118]. This effect is mainly attributed to the two distinct phases that SMAs exhibit at different temperatures: austenite at higher temperatures and martensite at lower temperatures. Cooling at a high rate or applying external forces can drive the transformation of austenite to martensite, resulting in deformation in the structure. This deformation is reversible by heating the material above a specific temperature, which induces the martensite to transform back to austenite [114]. In addition to their SME, SMAs exhibit also other unique properties, such as superelasticity, significant damping capacity, and excellent corrosion resistance. These properties make them attractive candidates for use in a wide range of applications, including biomedical devices, aerospace, robotics, actuators, sensors, and energy harvesting devices.

**Fig. 9:**
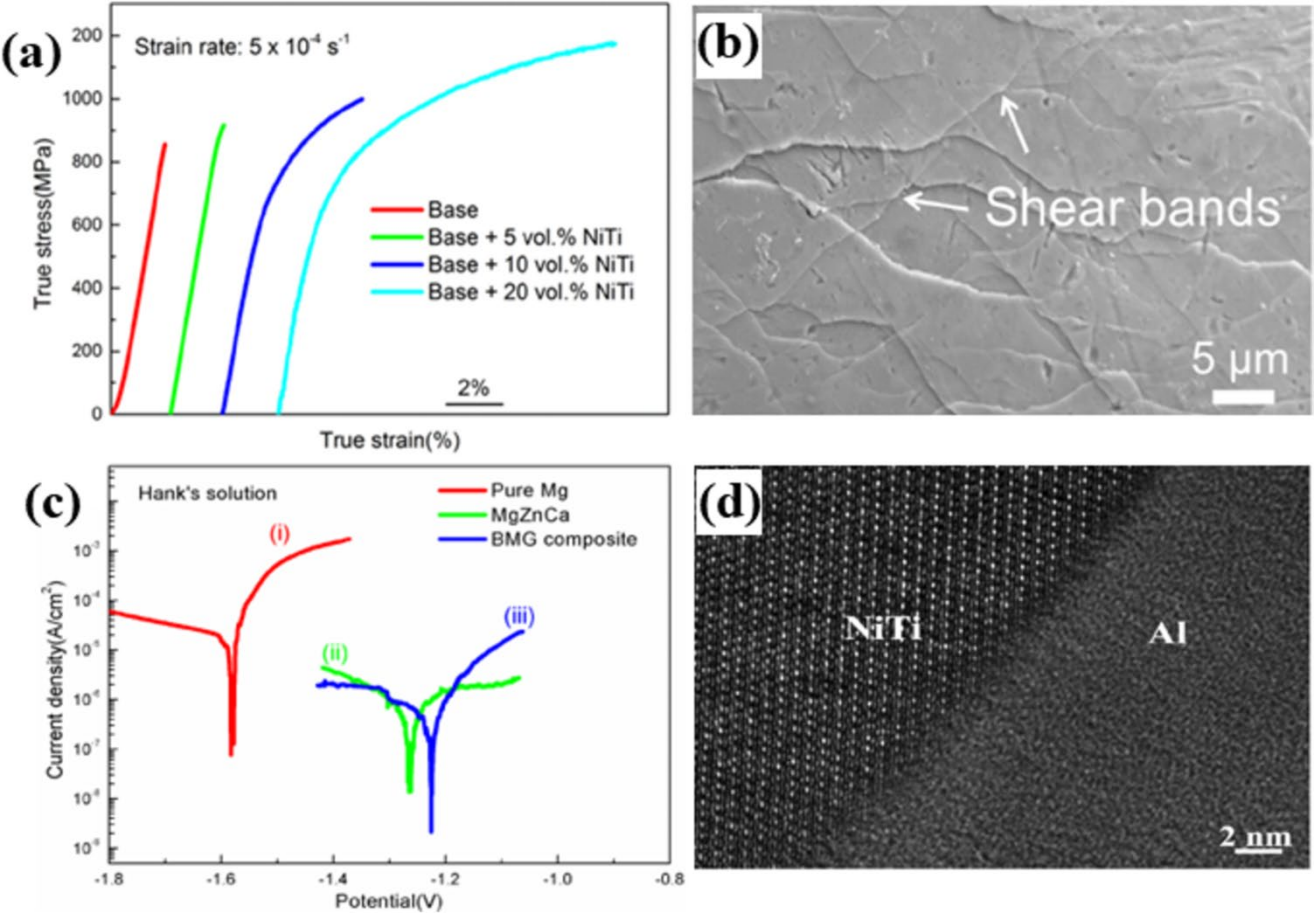
(**a**) Compressive stress–strain curves of unmodified and modified Mg_59.5_Cu_22.9_Gd_11_Ag_6.6_ matrix by porous NiTi particles [111] (reprinted with permission from Elsevier B.V., Copyright © 2016). (**b**) SEM image from the surface of the Mg_59.5_Cu_22.9_Gd_11_Ag_6.6_ matrix with 20 vol.% porous NiTi [111] (reprinted with permission from Elsevier B.V., Copyright © 2016). (**c**) Potentiodynamic polarization results of pure Mg, Mg_67_Zn_29_Ca_4_ alloy, and Mg_67_Zn_29_Ca_4_/NiTi BMG composite [112] (reprinted with permission from MDPI, Copyright © 2018). (**d**) HRTEM of heat-treated 6061Al/NiTi composite fabricated by FSP process [110] (reprinted with permission from Elsevier B.V., Copyright © 2014)

These unique properties have led to the development of a new type of high-performance MMCs that incorporates embedded SMA particles within metal matrices. One of the main purposes of adding SMA particles to a metal matrix is to enhance the compressive and room temperature tensile plasticity of BMGs, which are prone to the formation of fatal cracks due to the rapid propagation of localized single shear bands. By incorporating SMAs into the BMG, the stress-induced phase transformation of the embedded SMA particles can help to alleviate stress concentration, promote the formation of multiple small shear bands, and induce work hardening. For instance, by incorporating 20 vol.% of porous NiTi into a Mg_59.5_Cu_22.9_Gd_11_Ag_6.6_ (at.%) matrix, not only SME was attributed to the composite but also its plastic strain and fracture strength increased by up to 10.6% and 1173 MPa, respectively (Fig. 9a). This is because the porous NiTi undergoes a stress-induced phase transformation that contributes to the release of stress concentration, forming multiple small SBs that induce work hardening (Fig. 9b) [111]. The phenomenon of work hardening has been also observed in other types of composites and verified through three-dimensional finite element simulations. The addition of Ni–Ti alloys into Zr-based BMG-Vitreloy-1 composites was reported to enhance the work hardening effect under uniaxial loading. This observation was correlated to the phase transformation between austenite to martensite in Ni–Ti alloys as well as the rise of carried stress by the embedded Ni–Ti particles [119].

In addition to its ability to improve the mechanical properties of metal matrix composites, porous NiTi has also been shown to enhance the corrosion resistance of Mg–Zn–Ca BMGs in Hank's solutions. Incorporating NiTi particles into the Mg_67_Zn_29_Ca_4_ composite resulted in a significant reduction in current density and increased positive potential compared to pure magnesium and the base alloy. This was demonstrated through typical potentiodynamic polarization curves (Fig. 9c) [112]. In addition to NiTi particles, CuZr SMA particles have also been shown to be effective in improving the mechanical properties of BMGs. Incorporating CuZr SMA particles with a volume fraction of 30% increased the compressive ductility of Cu_51.5_Zr_48.5_ BMG [113]. This enhancement was attributed to three distinct energy-absorbing mechanisms: martensitic transformation of the CuZr particles, microcracking, and crack deflection. The martensitic transformation of the CuZr particles contributed to the energy absorption by inducing local plastic deformation and promoting the formation of SBs in the BMG matrix. Microcracking, which occurred at the interface between the martensite and parent phase, reduced the localized stress concentration and further promoted plastic deformation. The crack deflection protects the tip of the crack and induces localized plasticity as the result of interactions between shear bands and microcracks [67].

To achieve optimal results, it is important to recognize and optimize the effect of different parameters on the properties of the composite. The volume fraction and size of SMA particles are among the most important parameters affecting tensile strain enhancement. A numerical study using a free volume model revealed that the SMA volume fraction is directly associated with the tensile ductility of the composites. Specifically, a composite with 32 vol.% SMA formed multiple SBs by inducing the intersection of the SBs and blocking their propagation, whereas a composite with 5 vol.% SMA exhibited localized plastic deformation in one SB without any blocking effect [120]. The size of NiTi particles also affects the tensile strength of the composite, with smaller particles resulting in higher strength and larger particles leading to lower strength. This may be due to the high surface area to volume ratio of small particles, which allows for a greater degree of interaction with the matrix [110].

Employing an appropriate fabrication method is another important factor in order to prepare proper samples. Powder metallurgy and hot extrusion are commonly used processes that expose the samples to elevated temperatures for a prolonged duration, resulting in intermediate compound formation. For instance, due to the reactions between NiTi reinforcements and Al2124 alloy matrix, numerous intermediate phases (such as Al_3_Ni and Al_3_Ti) were formed, which had a negative impact on the SME and mechanical properties of the obtained composite [117]. FSP is an effective method for fabricating SMA composites with good SME and mechanical properties. The use of FSP has resulted in significant improvements in the yield and UTS of Al1100/NiTi particle composites, with an increase of approximately 70% [121]. This is a testament to the unique capabilities of the FSP process. There are two ways to make the FSP process more efficient: first, using a well-designed multi-hole particle mode for trapping secondary particles has been found to be more effective than using a groove or a few holes. This process distributes Ni_49.5_Ti_50.5_ (at.%) particles homogeneously in the Al6061–T651 matrix without interfacial reactions, resulting in higher ductility [110]. Second, aging at low temperatures after heat treatment for a short duration can further strengthen the composite. This results in high UTS and ductility without forming intermediate phases or negatively impacting SME (Fig. 9d) [110]. Apart from the FSP process, pretreatment strain also influences the final mechanical properties of composites reinforced by SMAs. Simulation results have revealed that increasing pretreatment strain (preloading process) to about 6.5% significantly enhances composite yield strength and reduces tensile plasticity, lowering the work-hardening ability of the composite. This can be attributed to the weakening of martensite phase transformation [120].

Overall, the unique properties of SMA particles have shown great potential in enhancing the mechanical properties of MMCs, and with further research on the interface between matrix and reinforcement and optimization of key parameters such as particle size, particles’ volume fraction, and fabrication methods, SMAs can become a promising option for a wide range of engineering applications.

### High entropy alloys

First introduced by Yeh et al. in 2004, high-entropy alloys (HEAs) are a novel family of structural materials, which are typically composed of five or more principal elements in equiatomic proportions [122]. The exceptional properties of HEAs take root from the following key factors: (1) high-entropy effect, which makes HEA elements reluctant to participate in reactions, resulting in considerable chemical stability even at elevated temperatures; (2) severe lattice distortion effect, which stems from different atomic radius of HEA constituent elements; (3) sluggish diffusion effect due to the distortion effect since severe distortions limit the solid diffusion process; and finally (4) cocktail effect, regarding HEAs being consisted of different elements, it can be considered as an atomic scale composite. These features offer the possibility to tune the properties of HEAs by altering their specific atomic compositions (for instance, increasing Al% would promote the formation of the BCC phase in HEA structure) [122, 123].

Recently, the use of HEA particles as reinforcements in MMCs has been expanded due to their superior characteristics such as ultra-high strength and ductility, good thermal stability, high-temperature mechanical properties, impressive wear, and corrosion resistance [124–127]. The metallic property of HEA is advantageous for boosting the interfacial strength of MMCs when compared to the ceramic reinforcing phase. MMCs reinforced by HEAs can be considered in the form of bulk structural materials and functional coatings or films [128–130]. Although introducing HEAs into the MMCs has solved the wettability problems of reinforcement up to considerable extents, controlling the interfacial reactions between the matrix and the reinforcement remains challenging for tunning the composite properties.

**Fig. 10:**
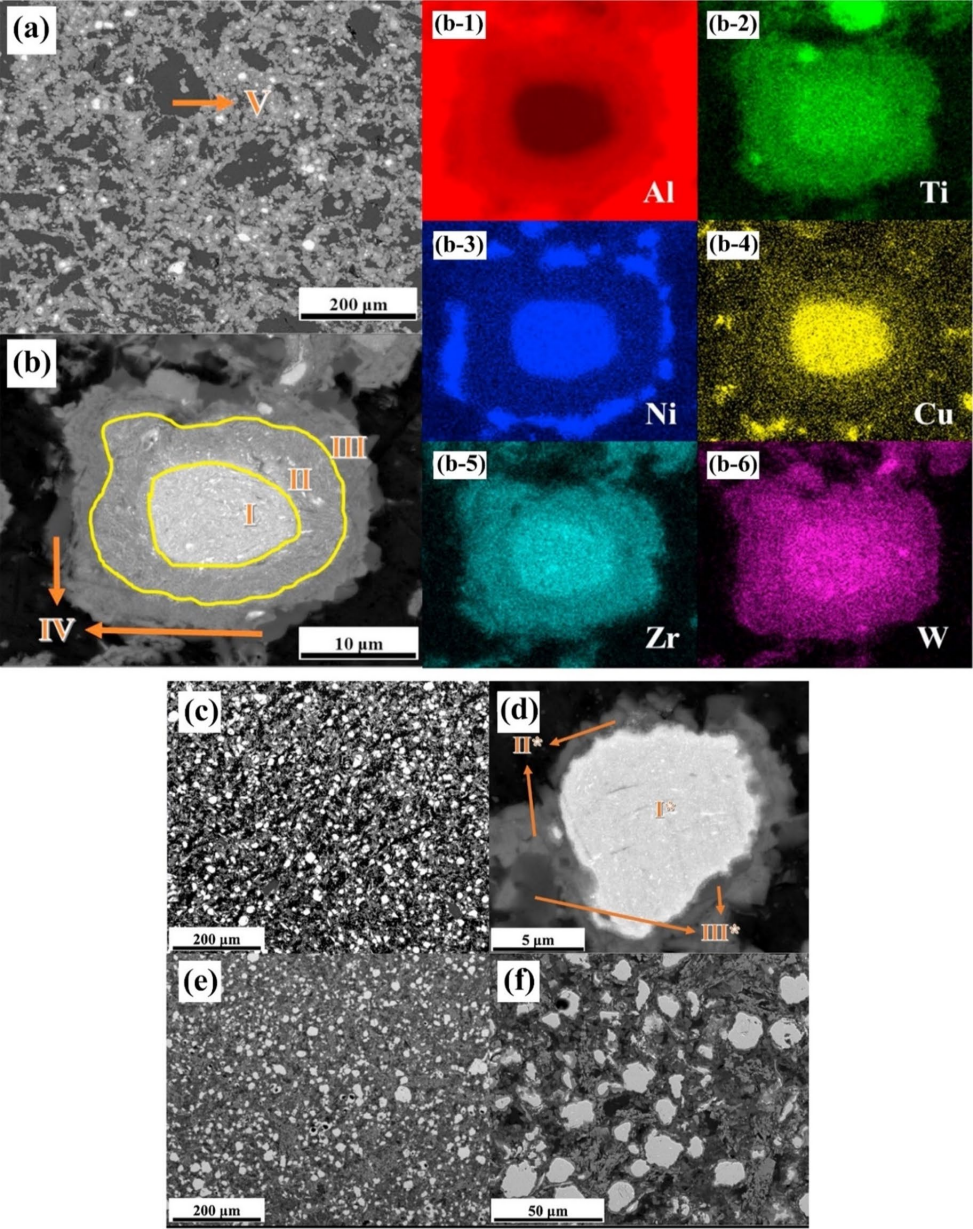
(**a**, **b**) FESEM patterns of Al/10%vol%CuZrAlTiNiW, (b-1–b-6) the elemental mappings corresponding to image (b), (**c**, **d**) FESEM patterns of Al/20%vol%CuZrAlTiNiW and (**e**, **f**) Al/30%vol%CuZrAlTiNiW composites [131] (reprinted with permission from Elsevier B.V., Copyright © 2020)

**Fig. 11:**
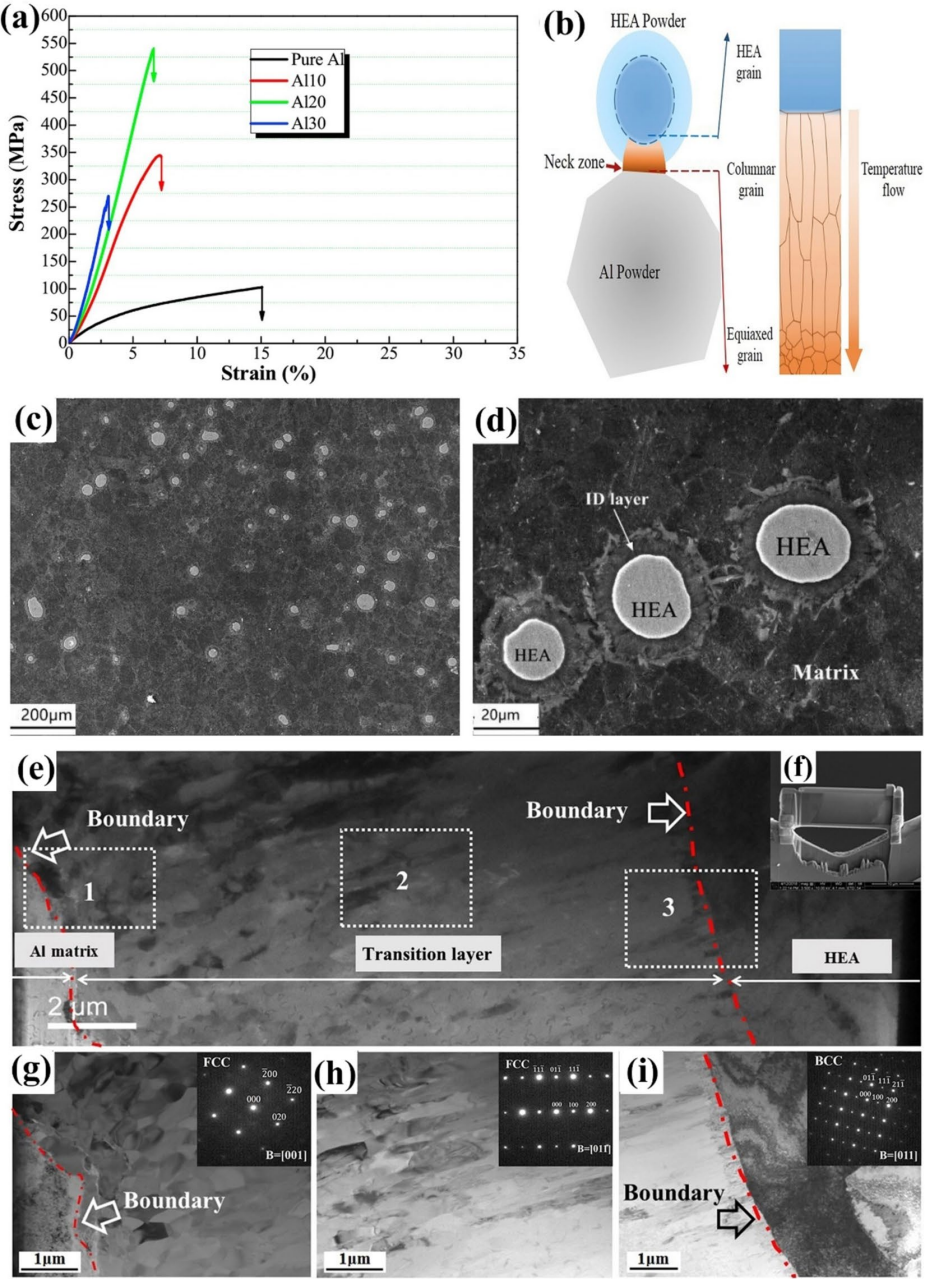
(**a**) Room-temperature compression stress–strain curves of the SPS-ed pure Al, and Al/10%vol%CuZrAlTiNiW, Al/20%vol%CuZrAlTiNiW, and Al/30%vol%CuZrAlTiNiW composites [131] (reprinted with permission from Elsevier B.V., Copyright © 2020). (**b**) Schematic diagram of the transition layer formation mechanism [135] (reprinted with permission from Elsevier B.V., Copyright © 2019). SEM images of the Al2024/CoCrFeMnNi): (**c**) low magnification, (**d**) high magnification [89] (reprinted with permission from Elsevier B.V., Copyright © 2019). (**e**) TEM images of the 5 vol.% AlCoCrFeNi reinforced Al matrix composite sintered at 600 °C, (**f**) the sample for TEM analysis prepared by FIB technique; (**g**–**i**) presents the microstructure of regions denoted by “1,” “2,” and “3” in (e) [135] (reprinted with permission from Elsevier B.V., Copyright © 2019)

Varying microstructures with different properties have been obtained based on different production procedures. Most recent surveys have focused on limiting the production of thick intermetallic compound formation at the interface to obtain hard composites with sufficient toughness to prevent the formation and propagation of brittle cracks. A common structure seen in many recent studies is known as core–shell structure. Wang et al. used SPS to prepare various samples of MMCs reinforced with different concentrations (10, 20, and 30 vol.%) of CuZrAlTiNiW HEA [131]. According to FESEM images of Al10 SPS-treated samples (Fig. 10a, b), there is a core–shell structure consisting of an Al-, Ti-, Ni-, Cu-, Zr-, and W-enriched core surrounded by an Al- and Ni–Cu-depleted egg-white-like shell. The core structure is BCC-HEA, whereas the shell structure is (Ti, Zr, W) Al_3_ phase with a cubic structure, according to the EDS data. Moving from the core toward the Al matrix, different zones, namely, the main shell structure (zone II), transition layer (zone III), and dark gray phase (zone IV) (Fig. 10b), can be observed. The FESEM images shown in Fig. 10c–f for the Al20 and Al30 composites reveal the presence of more precipitates in these samples. Moreover, a lack of fried egg–like structure is observed. In Al20 and Al30 samples moving from the core toward the shell, WAl12 phase (denoted by zone II) can be observed, followed by the dark gray phase (denoted by zone III corresponding to zone IV of the Al10 sample) and finally Al matrix (denoted by zone IV). The larger concentration gradient of Al in Al10 sample explains the difference in the structure between Al10 and Al20/Al30 samples. Al diffusion can promote the formation of egg-white structure, which was more obvious in Al10 sample. It is accepted that HEA particles with high lattice distortions exhibit less thermal conductivity than Al matrix [132], thus heat can be quickly transferred by the Al matrix from the area of contact with HEA particles. This facilitates the diffusion of Al into the HEA particles, leading to the in situ formation of WAl12 and Ni–Al rich B2 phases around the reinforcing particles. The higher the density of the HEA particles promoted a higher microhardness resulting in the highest obtained value of about 331 HV for the Al30 sample which is ten times higher than that of pure Al. This hardness enhancement was attributed to the HEA BCC phase and B2 and WAl12 phases, which hinder the dislocations’ movement and enhance the plastic deformation resistance. According to the compression stress–strain curve in Fig. 11a, in Al10 sample, the presence of core–shell structure effectively coordinated the deformation between HEA particles and the matrix [131]. This coordination helped in passivating the crack tips when cracks reached the core–shell structure, which in turn preserved the ductility of the material. On the other hand, the Al20 composite showed the most remarkable increase in UTS and σ_0.2_ (yield stress), but also experienced the most severe ductility loss among all the samples. These strength and hardness improvements are provided by precipitation strengthening of a phase that was formed in situ paired by dispersion strengthening from BCC reinforcing particles. However, in the Al30 composite, there was a large reduction in UTS. Despite having an average particle size similar to that of the Al20 composite, the precipitations in the Al30 composite were not well distributed. According to Lu et al. [133], insufficient precipitation content or nonuniform distribution can prevent further improvement of the mechanical properties of the composite. Moreover, the higher thickness of the transition layer in Al30 sample was found to be harmful to σ_f_ and σ_0.2_ values as was proved in the previous studies [134].

Using the same production procedure, Yuan et al. [89] found structures similar to the previously described core–shell structure using CoCrFeMnNi HEA in an Al matrix. Due to the different diffusion rates of HEA elements in solid and liquid aluminum, the structure of the interdiffusion layer was laminated and serrated, as seen in Fig. 11c and d. According to the microhardness tests, an increase in creep depth was found as the distance from the HEA particles through the matrix increased. However, there was no significant increase in the creep depth when the indentation tip was pointed toward the interior of the particles. When the tip was pointed near the edge of the particles, the creep depth varied from 1.3 to 2.8 nm, also indicating an increase in Al concentration. The average hardness of the reinforcing particles was much higher than that of the composite, with average hardness values of 277 HV_0.05_ for the HEAs, 80 HV_0.05_ for the matrix and 131 HV_0.05_ for composite samples [89].

Utilizing sintering at different temperatures may be an efficient remedy to avoid the transition layer formation in MMCs [135]. The absence of a transition layer in the sintered AlCoCrFeNi HEA/Al samples at 540 °C was a consequence of this microstructure modification. However, optimizing the sintering temperature is a vital issue as higher temperatures induce the formation of thicker transition layers. At higher temperatures, transition layers appeared in the samples. Detailed TEM analysis in Fig. 11e–i shows how the transition layer phase changes from BCC near the border of HEA particles with BCC phase to FCC phase near the border of Al matrix with FCC phase. Compared to the grain size of reinforcements and the matrix, which are 20 and 4 µm, respectively, transition layer grains are much finer (about 600 nm). The reason for such a structure in the transition layer is the inhomogeneous distribution of temperature in the composite during the sintering. According to experimental findings and also numerical simulations, the border area between the particles and the matrix has the highest temperature during sintering, which is above the melting temperature of the amorphous matrix [132, 136, 137]. Besides, it is generally agreed that HEAs with higher lattice distortions have lower thermal conductivity [138]. Combining this temperature effect with higher thermal conductivity of the matrix compared to the HEA particles (about 20 times higher), moving from particles interior toward the matrix, temperature drops significantly near the matrix as heat is transmitted through the matrix. This is the reason behind the formation of a columnar grain structure near the HEA border followed by fine equiaxed grains near the matrix, as shown in Fig. 11b.

**Fig. 12:**
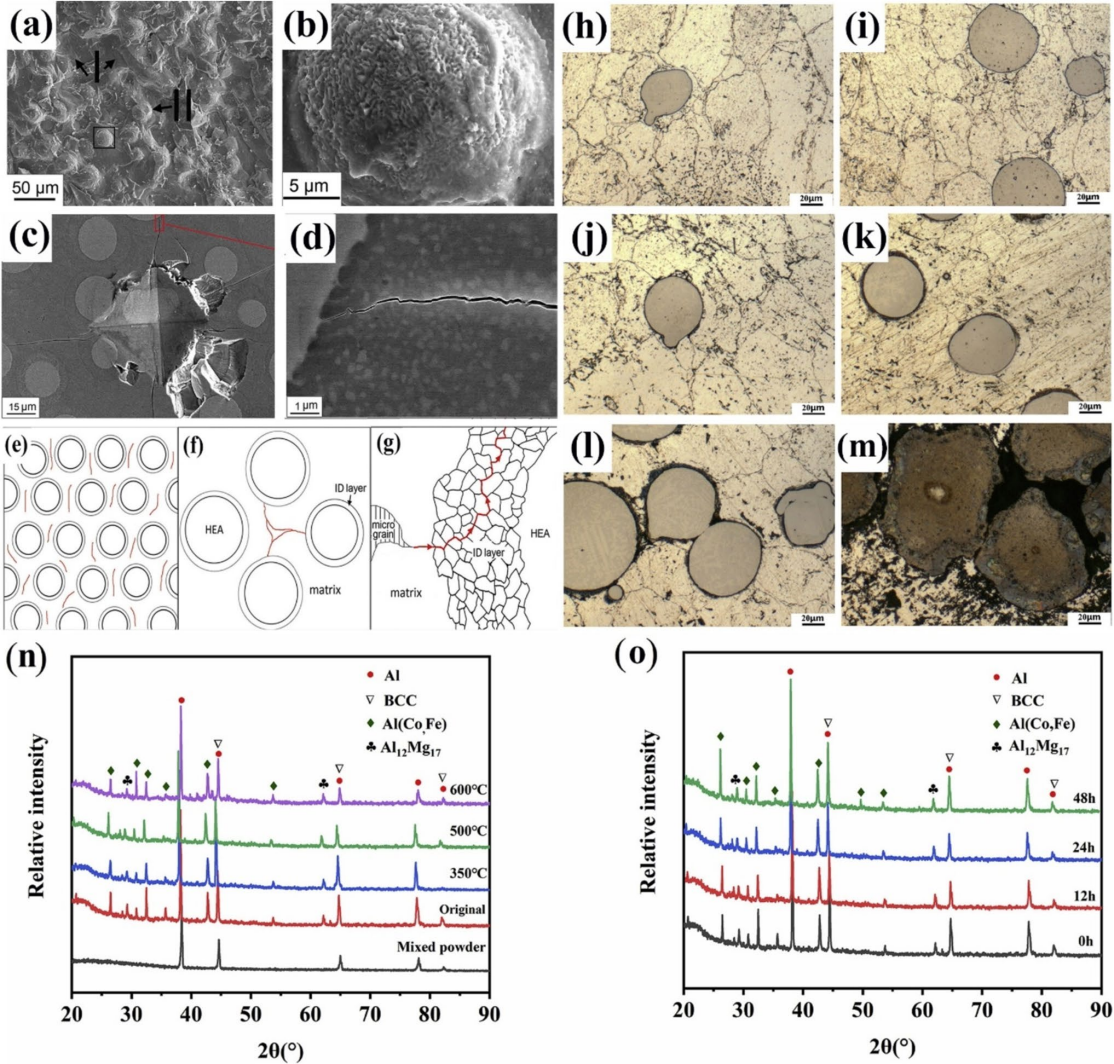
SEM images of the fracture surface morphology at (**a**) low magnification and (**b**) high magnification, (**c**–**d**) crack propagation paths in the composite (Al_65_Cu_16.5_Ti_18.5_/Al_0.6_CoCrFeNi) and (**e**–**g**) the sketch of the crack propagation paths in different regions of the composite [141] (reprinted with permission from Elsevier B.V., Copyright © 2016). (h–m) Metallographic images of composites (Al5058/Al_0.6_CoCrFeNi) under different heat treatment conditions; (**h**) original sample, (**i**) 350 °C + 24 h, (**j**) 500 °C + 12 h, (**k**) 500 °C + 24 h, (**l**) 500 °C + 48 h, (**m**) 600 °C + 24, and XRD patterns of the composites (Al5052/Al_0.6_CoCrFeNi) under different heat treatment conditions. (**n**) Heat treatment at different temperatures at 24 h; (**o**) heat treatment at 500 °C at different times [142] (reprinted with permission from Elsevier B.V., Copyright © 2020)

Analyzing fracture surfaces is highly valuable as it can reveal the reason behind, and the mechanism associated with damage initiation and evolution. Fractography analysis of a MMC with Al_65_Cu_16.5_Ti_18.5_ amorphous alloy as the matrix and Al_0.6_CoCrFeNi HEA particles as reinforcement indicated the amorphous nature of the matrix as the main cause of composite fracture, considering the intrinsic brittle characteristics of both the matrix and reinforcement. Besides, the presence of microcracks as sintering defects in the matrix suggested that these cracks could propagate in the matrix under loading. The notable difference between the microhardness of the matrix (~ 16 GPa) and the interdiffusion layer and HEA particles (~ 14 GPa and ~ 9 GPa, respectively) led to stress concentration at the interface area between interdiffusion layer and HEA particles under loading. Since HEA particles can plastically deform, they show better load-bearing ability; therefore, it is assumed that the matrix is mainly responsible for the failure of the MMC. SEM analysis of fracture surfaces in Fig. 12a and b exhibit two types of textures: mirror-like area and dome-like area. Numerous pits and tubers on the surface of HEA spheres resulted from the fracturing of submicron grains of the interdiffusion layer, as seen in a magnified view of the dome-like area. Microcracks propagated in linear paths in the matrix under loading conditions (Fig. 12c–g), until they reached interdiffusion areas, where they were deflected. Since interdiffusion layers were made up of submicron grains, cracks continued to propagate along grain boundaries. This intergranular propagation path increased the fracture surface, resulting in higher composite strength [139, 140]. Furthermore, increasing the volume percentage of HEA particles (thus increasing the interdiffusion layer volume fraction) reduced the amount of amorphous phase and the number of defects in the matrix, resulting in increased composite strength. However, the composite had poor flexibility since the hard interdiffusion layer was unable to efficiently coordinate deformation toward the HEA particles [141].

Yuan et al. [142] employed hot vacuum sintering to manufacture samples with different HEA contents, utilizing commercially available Al5052 as the matrix and Al_0.6_CoCrFeNi powder as the reinforcement. Microstructural analysis (Fig. 12h–m) revealed the presence of dark gray Al_0.6_CoCrFeNi particles, black second-phase particles, and the yellowish-gray background phase as the Al alloy matrix. According to the XRD results, the Al_0.6_CoCrFeNi particles had a single-phase BCC solid solution structure. However, after sintering, a new phase of Al (Co, Fe) and Al_12_Mg_17_ emerged in the composite. The increased diffraction peaks shift with the increase in the heat treatment temperature (Fig. 12n, o) could be attributed to the lattice distortion caused by the diffusion of HEA elements into the Al matrix [131]. Moreover, mechanical tests revealed that heat treatment time had a limited effect on Young’s modulus but a huge impact on hardness values. The thickness of the interfacial layer raised as the heat treatment duration increased, lowering the composite's Young's modulus and hardness. Hardness was increased from 1.43 GPa in the original sample to 1.81 and 1.60 GPa in the samples heat treated at 500 °C for 12 and 24 h, respectively. This can be attributed to the transformation of the discontinuous thin interfacial layer into a continuous layer, the increase in its thickness, and the release of accumulated stresses within this layer [142].

**Fig. 13:**
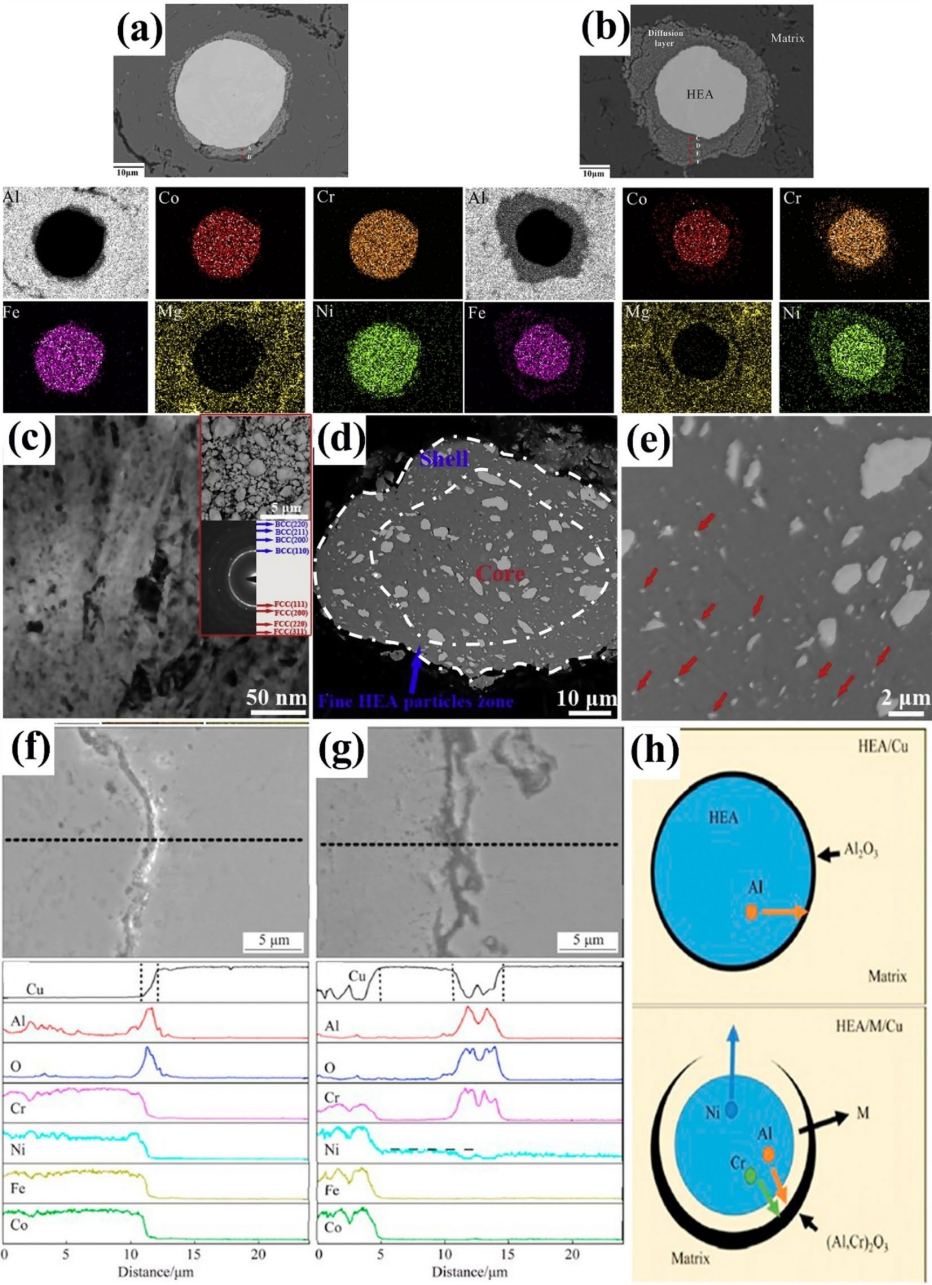
Elemental distribution of composite materials (Al5052/Al_0.6_CoCrFeNi) (**a**) before heat treatment; (**b**) heat treated at 500 °C for 24 h [142] (reprinted with permission from Elsevier B.V., Copyright © 2020); (**c**) bright-field TEM image of the 60 h milled CoNiFeCrAl_0.6_Ti_0.4_ HEA powder; (**d**, **e**) BSE images of the 15-h milled Al2024/7.5HEA composite powder [143] (reprinted with permission from Elsevier B.V., Copyright © 2019). EPMA line distributions of (**f**) HEA/Cu and (**g**) HEA/M/Cu composites at interface and (**h**) schematic diagrams of the effect of transition layer structure on elemental diffusion during sintering for HEA/Cu and HEA/M/Cu composites [144] (reprinted with permission from Elsevier B.V., Copyright © 2019)

The elemental distribution data at 500 °C compared to non-heat-treated samples (Fig. 13a, b) demonstrate a different distribution of alloying elements, which is due to the hysteresis characteristic of diffusion kinetics of HEA and the difference in the diffusion coefficient of each element. The larger diffusion of Fe and Ni in Al matrix hinders the diffusion of Cr, as we can see its lower distribution in the matrix.

Nanocrystalline CoNiFeCrAl_0.6_Ti_0.4_ HEAs were employed in another study by Lu et al. [143] to evaluate the effect of existing large grain boundaries as non-equilibrium states on grain size. A core–shell structure, consisting of a core of bigger HEA particles and a shell area with smaller particles, can be seen in Fig. 13d and e. The results of bright-field TEM confirm the nanocrystalline stability of HEA in the final sample (Fig. 13c), which is a consequence of the sluggish diffusion of HEA elements and their great thermal stability, as also reported in previous studies [145, 146]. HEA reinforcements could significantly promote the grain refinement, in the way that with the addition of a higher amount of HEA in the composite, average grain size reduced from 5.1 to 1.1 µm which was the result of the pinning effect on grain boundary movements in the matrix.

In order to boost the diffusion of HEA constituents at the interfacial layer, Yu et al. [144] utilized Al_0.3_CoCrFeNi HEAs in a Cu matrix. They prepared two sets of samples: the first group involved direct sintering of milled HEA and Cu particles (HEA/Cu), while the second group involved ball milling of HEA particles with M powder (M represents transition layer elements such as Cu) prior to adding them to the matrix (HEA/M/Cu). The formation of numerous defects and new free surfaces on HEA particles covered with Cu allows for diffusion to occur at lower temperatures compared to the typical diffusion temperatures, ultimately resulting in a homogeneous composition through simultaneous particle diffusion and dissolution. The formation of the transition Cu layer had a significant impact on the mechanical properties of the composite. The EPMA (electron probe microanalysis) (Fig. 13f, g) revealed that the additional ball milling process significantly altered the distribution of different elements in the HEA. The formation of a transition layer caused the accumulation of Cr and Al elements in the interface layer. Moreover, the presence of oxygen at the interface facilitated the formation of (Al,Cr)_2_O_3_. Differential thermogravimetric analysis results illustrated evidence for a chemical reaction in the HEA/M/Cu sample, which may have been caused by the reaction between Cr, Al, and oxygen contamination. The transition layer also had an impact on the diffusion pathway, facilitating the diffusion of elements such as Ni and resulting in the accumulation of Al and Cr in the interface layer to form complex oxides (Fig. 13h).

The overall hardness of the HEA/M/Cu composite (60 HV) was about 20% higher than that of the unreinforced matrix. However, the hardness of the HEA particles in the HEA/Cu sample was higher. This may be explained by the accelerated diffusion rate of different elements in the HEA/M/Cu sample, facilitated by the development of the transition layer. Additionally, tribological tests showed more severe grooves on the HEA/Cu sample, indicating a 30% higher wear resistance for the HEA/M/Cu sample, compared to the sample prepared without the milling step, due to the formation of the transition layer [144].

**Fig. 14:**
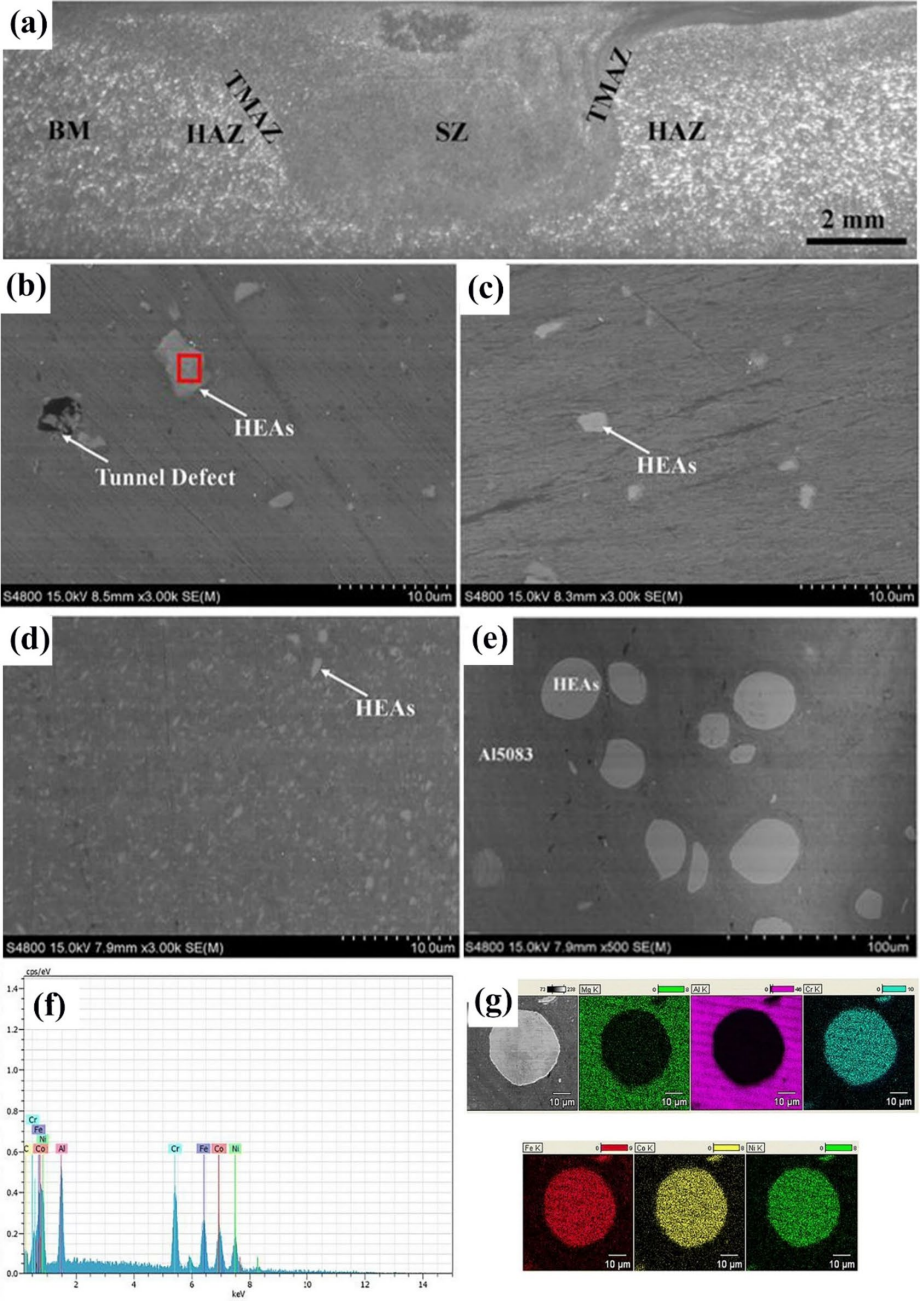
(**a**) Macroscopic overview of cross section of 5-pass FSPed composite (Al5083/FeCoNiCrAl), SEM images of composite fabricated with different processing passes: (**b**) 1 pass, (**c**) 3 passes, (**d**) 5 passes, and (**e**) SEM image of edge of FSP zone. (**f**) EDS spectrum of the red box in (**b**), and (**g**) EDS elemental mapping [147] (reprinted with permission from Springer Nature, Copyright © 2020)

**Fig. 15:**
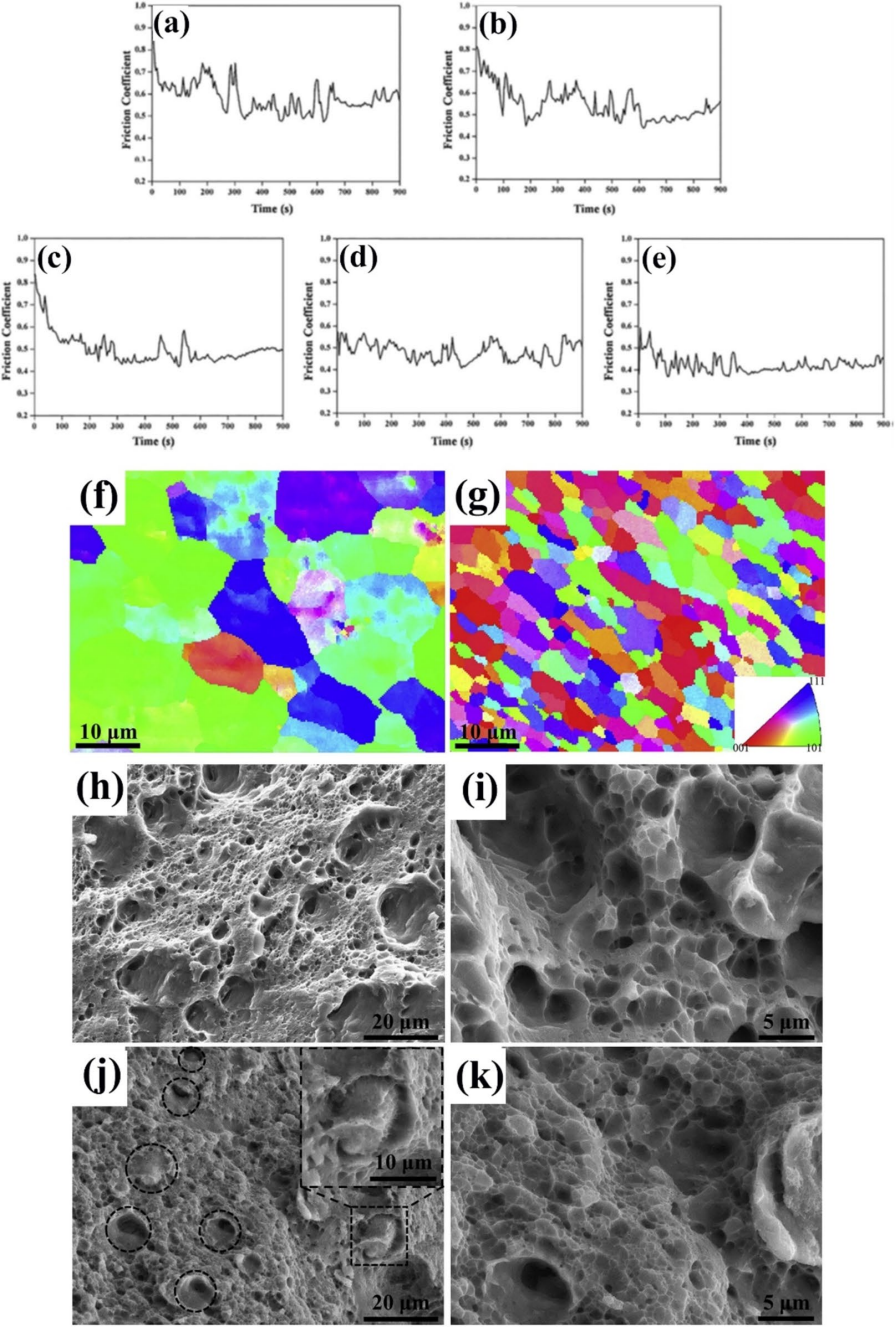
Variation of friction coefficient with time in composite (Al5083/FeCoNiCrAl) in (**a**) base material, (**b**) without particles 1 pass, (**c**) with particles 1 pass, (**d**) with particles 3 pass, and (**e**) with particles 5 pass [147] (reprinted with permission from Springer Nature, Copyright © 2020). IPF maps of composite (Al5083/Al_0.8_CoCrFeNi); (**f**) the FSPed Al alloys and (**g**) the FSPed MMCs, the legend shows the IPF coloring of Al, (h–k) fractographs for (**h**, **i**) the FSPed Al alloys and (**j**, **k**) the FSPed MMCs [148] (reprinted with permission from Elsevier B.V., Copyright © 2020)

Among various implemented manufacturing methods, FSP is believed to successfully limit the formation of serious interfacial reactions. In research conducted by Gao et al. [147], FSP was used to prepare Al5083 reinforced by FeCoNiCrAl HEA, focusing on the effect of the number of FSP passes. According to the SEM micrographs and EDS maps (Fig. 14f, g), the clear boundary observed between the particles and the matrix demonstrated that no interfacial reaction occurred throughout the procedure, which is in line with the findings of prior research [142, 148]. This is due to the relatively short time needed for FSP and lower process temperature compared to the melting point of HEA particles (1350 °C). Moreover, four distinct zones were detected, namely, stir zone (SZ), thermo-mechanically affected zone (TMAZ), heat affected zone (HAZ), and base material (BM). In SZ, which is the most affected zone by the heat and plastic flow, recrystallization occurred and equiaxed small grains were formed. HAZ and TMAZ are considered transition zones between BM and SZ, where deformation occurred but no recrystallization was observed (Fig. 14a). By increasing the number of passes, according to Fig. 14b–e, particle distribution became more uniform, particle breakage occurred more frequently, and tunneling defects were eliminated. It is worth mentioning that non-uniformity in particle distribution still exists at the edges of friction stir zones due to the lack of plastic flow. Regarding Fig. 15a–e, the general wear resistance of composite improved by increasing the number of passes and further grain refinement resulted from addition of HEAs, i.e., fluctuations in friction–time curves decreased, and the wear mechanism shifted from adhesive wear to moderate abrasive wear.

The addition of HEA particles and increasing the number of passes enhanced the average microhardness from 78 to 158 HV, which can be attributed to several factors. First, the hardness of the base material increased after one pass due to grain refinement, which is explained by the Hall–Petch relation. Second, the addition of reinforcing particles improved the hardness by obstructing the dislocations, according to the Orowan mechanism. Furthermore, the difference in thermal contractions between the HEA particles and the matrix also contributes to the increased hardness due to the quench hardening effect.

Li et al. [148] used FSP to fabricate commercially available Al (AA5083-H111) matrix reinforced with pre-alloyed AlCoCrFeNi particles. During the FSP treatment, samples experienced an interfacial reaction, which gave rise to an interdiffusion layer between the particles and the matrix. In comparison to samples produced through SPS, those produced through FSP demonstrated a much thinner diffusion layer, without the presence of any intermetallic compounds. This can be attributed to the lower working temperature of FSP, which enhances the bonding between the matrix and particles [148]. Particles’ dispersion during FSP is promoted through SPD, with fully overlapping passes minimizing any asymmetry of particle flow in the SZ. Following four FSP passes, HEA particles maintained their spherical shape, indicating their excellent mechanical stability. The inverse pole figure (IPF) map presented in Fig. 15f and g shows equiaxed grain morphologies for both Al alloys and MMC. As dynamic recrystallization occurs during FSP, the addition of HEA particles resulted in a reduction in grain size. The average grain size for the Al alloy sample ranged between 7 and 14 µm, whereas for the MMC sample, it ranged from 2.8 to 4.6 µm. The presence of reinforcing particles with sizes larger than 0.5 µm promoted recrystallization through the particle-stimulated nucleation mechanism [149]. The interfacial region between the HEA particles and matrix was found to be continuous and compact. In addition, an Al concentration gradient layer (with a thickness of less than 1.0 µm) was observed, which was attributed to the diffusion of Al into the HEA particles. This phenomenon has already been demonstrated in other studies [128, 135, 150]. The absence of intermetallic compounds at the interfacial region, along with miniature interfacial diffusion, resulted in a clear and compact interfacial layer, which exhibited excellent load-bearing abilities within the composite.

The factors that have been reported to influence the mechanical properties of samples produced through FSP include grain refinement, the formation of a thin interfacial layer without the intermetallic compounds, and the uniform distribution of reinforcing particles throughout the matrix [148]. The average hardness recorded in the SZ for FSPed MMCs was 125.7 HV_0.2_, whereas for FSPed Al it was about 80 HV_0.2_. The fractography analysis presented in Fig. 15 h–k depicts the ductile nature of the fracture for both samples, whereas the wider and deeper dimples in AMC samples indicate the composite's lack of ductility. The presence of HEAs at the bottom of dimples without breaking and detachment is evidence of good matrix–reinforcement interfacial bonding.

**Fig. 16:**
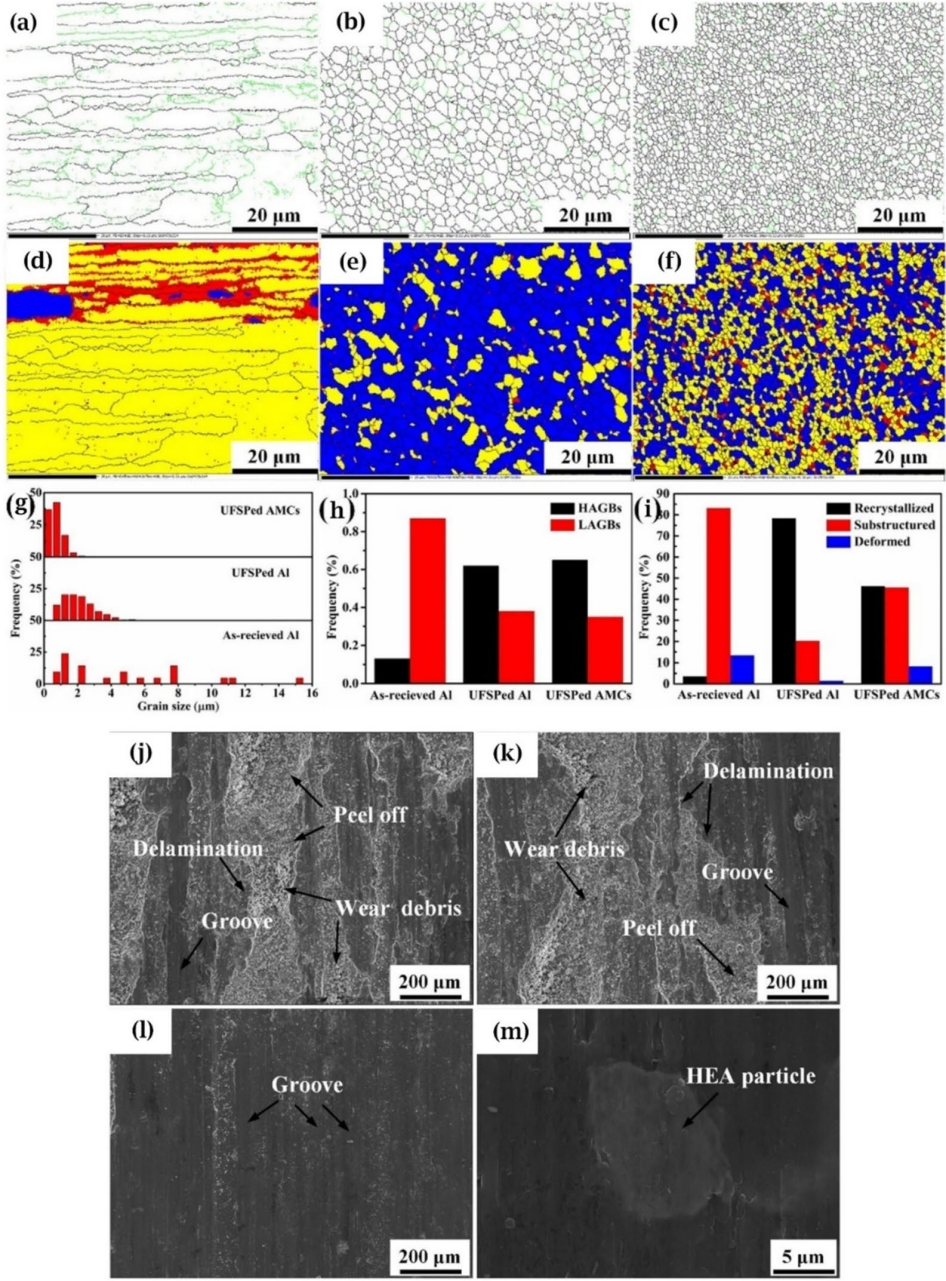
(**a**–**c**) EBSD results of the as-received Al, UFSPed Al, and UFSPed MMC (AA5083/CoCrFeNi), (**d**–**f**) the inverse pole figure (IPF), recrystallization maps, (**g**) grain size distribution, (**h**) summarized results of high- and low-angle grain boundaries, (**i**) summarized results of recrystallized, sub-structured, and deformed region and SEM morphologies of worn surfaces for (**j**) the as-received Al, (**k**) UFSPed Al, (**l**) UFSPed AMCs, and (**m**) HEA particles [158] (reprinted with permission from Elsevier B.V., Copyright © 2020)

**Fig. 17:**
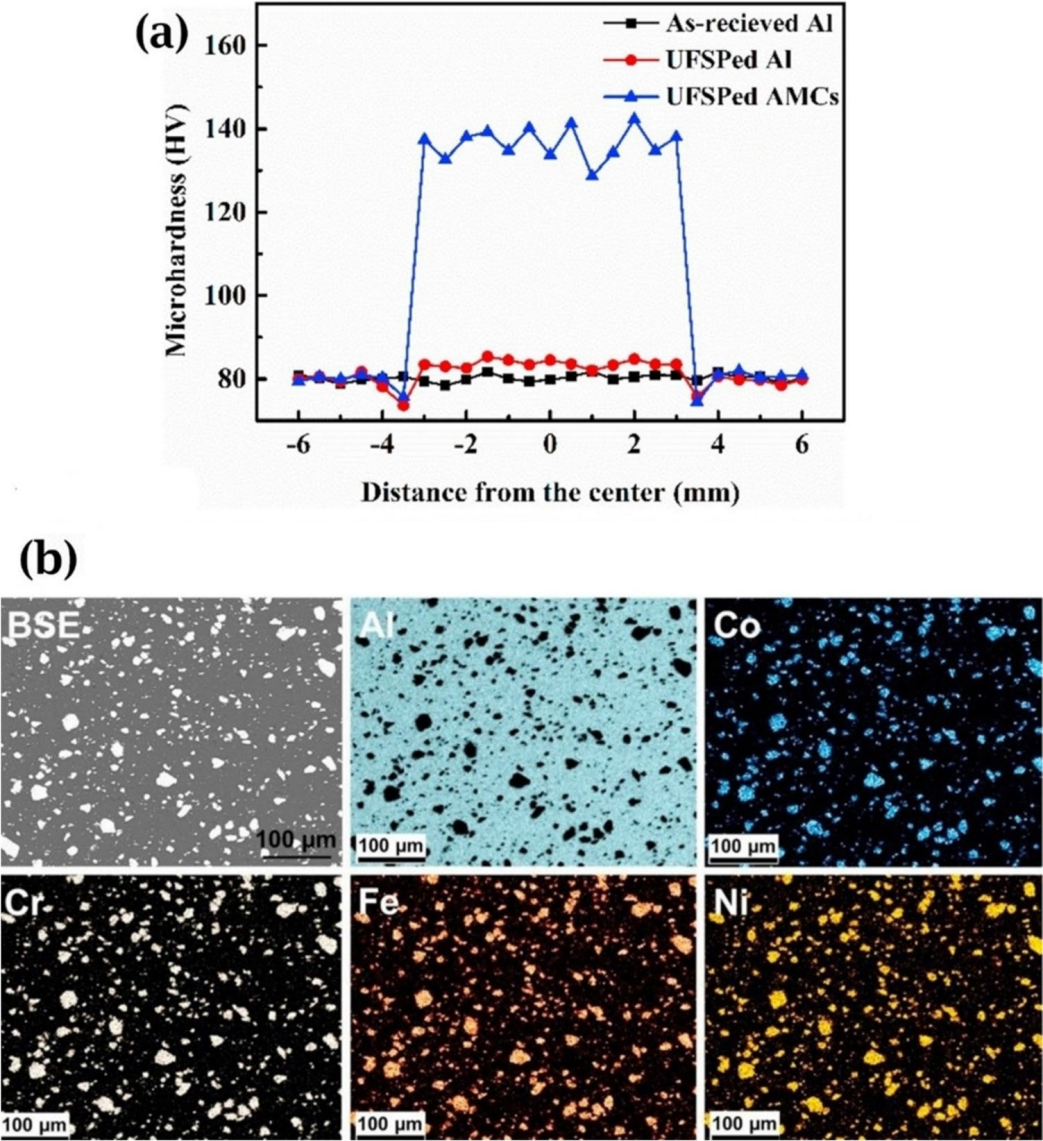
(**a**) The hardness profiles across the cross-section of the as-received Al, UFSPed Al, and UFSPed MMC (AA5083/CoCrFeNi), and (**b**) SEM–EDS elemental maps of composite (AA5083/CoCrFeNi) [161] (reprinted with permission from Elsevier B.V., Copyright © 2017)

An interesting alteration to FSP is underwater FSP (UFSP), which is exploited to prevent the development of intermetallic compounds at the interface. Through lowering the working temperature, UFSP can also minimize the risk of interfacial reactions between the HEA particles and the matrix. Another advantage is the prevention of density gradient formation, which is a common phenomenon at higher temperatures caused by matrix melting [150–154]. The thickness of the formed interface layer in UFSP is roughly 200 nm, which is comparatively thinner than the interfacial layer found in composites produced by other methods, such as laser melt injection, laser additive synthesis, and SPS [135, 155, 156]. Due to this thin layer, the development of microcracks at the interface may be avoided [141, 157] and load transfer from the matrix to the particles during sliding wear can also be improved. The UFSPed samples exhibit a greater number of high angle grain boundaries (black lines) than the as-received and the UFSPed Al samples (Fig. 16a–c). The addition of HEA particles increased the number of dislocations in the interfacial area, resulting from differences in CTEs and elastic modulus between the HEA particles and the matrix. Furthermore, broken HEA particles and already-formed small intermetallic compounds acted as nucleation sites, facilitating recrystallization through the particle stimulated nucleation (PSN) mechanism. In Fig. 16d–i, the amount of recrystallized (blue), sub-structured (yellow), and deformed (red) grains in different samples are shown. The UFSPed samples exhibit a higher fraction of recrystallized grains, while the UFSPed AMCs show a greater number of sub-structured grains, indicating higher matrix deformation due to the incorporation of HEA particles. SEM analysis of the wear surface of the as-received Al and UFSPed Al in Fig. 16j–m indicates that adhesive wear was the primary wear mechanism in these samples, as evidenced by the signs of delamination, wear debris, peeling off sites, and deep grooves. Also, the presence of tiny grooves and debris in UFSPed AMCs suggests that abrasive wear was the major wear mechanism at play [158]. The study found that FSP had a minimal effect on the hardness improvement in plain, unreinforced samples of Al. However, it dramatically increased hardness values in MMCs. Figure 17 depicts the limited effect of UFSP on the hardness of as-received Al samples. During UFSP, the grain refinement effect due to dynamic recrystallization and work-hardening increases the hardness, but this process also leads to the release of residual stresses and a decrease in dislocation density, which can offset the hardness improvement. The refinement of grains improves hardness through Hall–Petch strengthening, while HEA particles and intermetallic compounds enhance hardness through Orowan strengthening. Bao et al. [159, 160] derived an equation for calculating the plasticity index of UFSPed MMCs (88.0%), which is lower than that of both as-received Al (91.8%) and UFSPed Al (91.2%). This indicates that the HEA particles increased the stiffness of the matrix. Besides, this difference in plasticity index between samples indicate that strengthening was not only affected by the HEA particles but also by the matrix itself [158].

Friction deposition is established as a new method for additive manufacturing of advanced composite materials. Al–Mg alloy matrix (AA5083) reinforced with nanocrystalline HEA particles of CoCrFeNi (12 vol.%) were fabricated utilizing this method [161]. To compare the results, several multi-layer monolithic (without the addition of HEA) friction deposited samples and a few single-layer friction stir-welded samples were also prepared. CoCrFeNi was chosen due to its stability against reacting with Al, thus the formation of brittle intermetallic compounds was mainly prohibited. As confirmed by EBDS analysis in Fig. 17b, no intermetallic compounds were formed. The crystallinity of the HEAs after the friction deposition was also confirmed by diffraction analysis [161]. During the friction deposition process, dynamic recrystallization occurred, resulting in a much finer grain size. Moreover, the difference in CTE between the reinforcements and the matrix leads to a significant increase in dislocation density. Other evidence, such as the formation of sub-grains and a high concentration of dispersoid particles (resulting from alloy 5083), further confirm the occurrence of the PSN mechanism [161]. Another study investigated the effects of ball milling time on a 6061Al matrix composite reinforced with 7.5% nanocrystalline CoNiFeAl_0.4_Ti_0.6_Cr_0.5_ [162]. Different samples were prepared to identify the optimized ball milling time (10, 20, and 40 h) to improve the mechanical properties [162]. The addition of reinforcements led to the formation of ultra-fine grains in the sample, which was the reason for the 5–6 times higher strength. However, these samples also exhibited limited ductility. Interestingly, the average size of particles increased during the first 20 h of ball milling, but then decreased again as some larger particles broke down, leading to an overall decrease in average particle size after 40 h of ball milling. The cross-sectional morphology of the three composite powder samples (10, 20, and 40 h) showed a more uniform distribution of reinforcing particles in the 10-h ball-milled sample. However, the banding phenomena, i.e., formation of clusters of HEA, and areas depleted from HEA particles were evident in both the 20-h and 40-h ball-milled samples. The formation of HEA clusters during the 40 h of ball milling induced recrystallization, giving rise to a heterogeneous grain structure consisting of a portion of ultra-fine, large, and elongated grains. The presence of fine particles tended to slow down grain boundary movement, which in turn delayed recrystallization and grain growth (known as the Zener drag effect). The fraction of the sample that underwent recrystallization increased with increasing milling time up to 20 h, but then decreased up to 40 h of milling time. These findings were consistent with those reported in other studies [163].

**Fig. 18:**
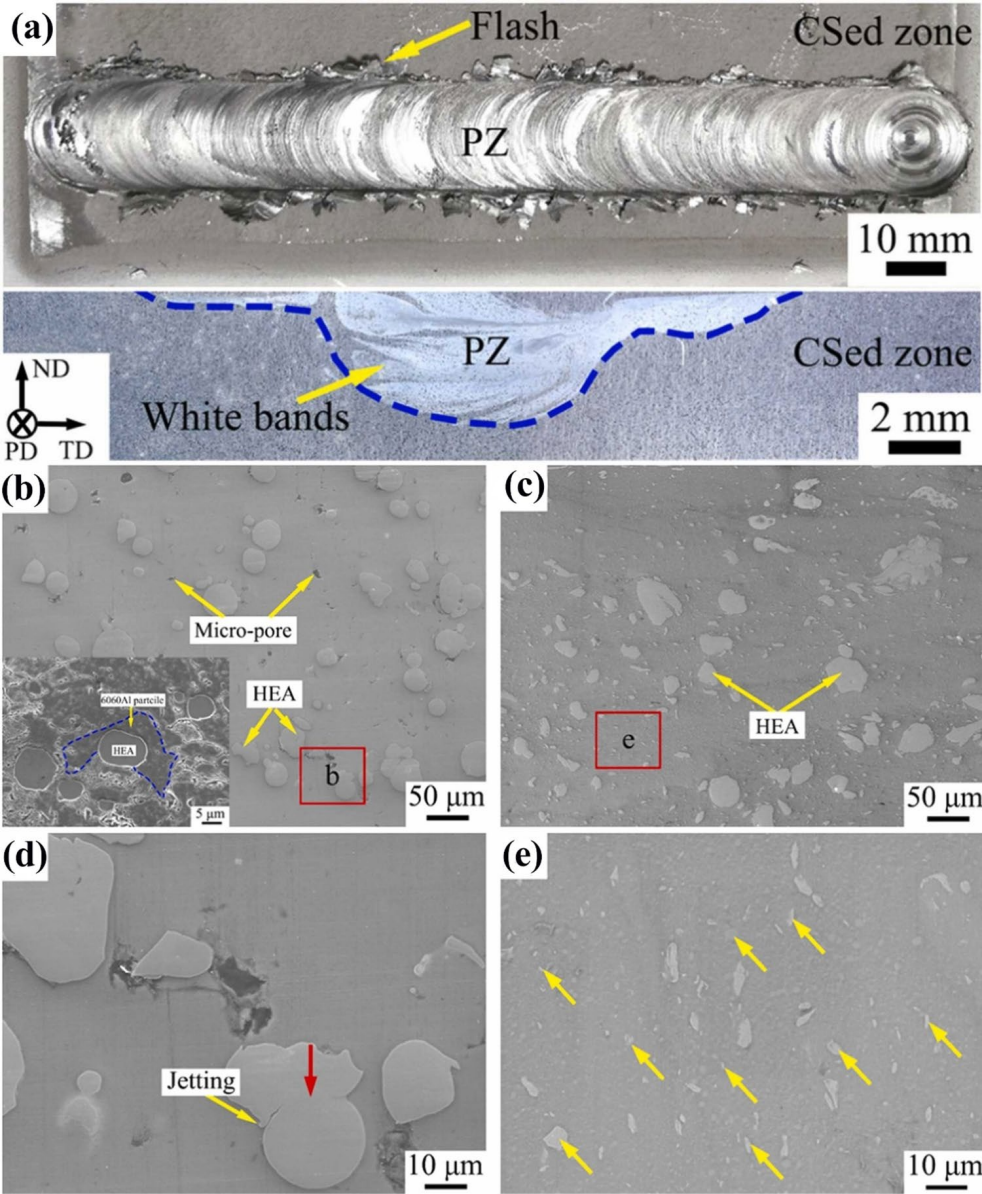
Macro-profiles and cross-section of cold sprayed deposits (6061Al/CoCrFeNi) after FSP (**a**). SEM images representing the cross-sectional morphologies of cold-sprayed sample (**b**, **d**) before and (**c**, **e**) after FSP [164] (reprinted with permission from Elsevier B.V., Copyright © 2022)

**Fig. 19:**
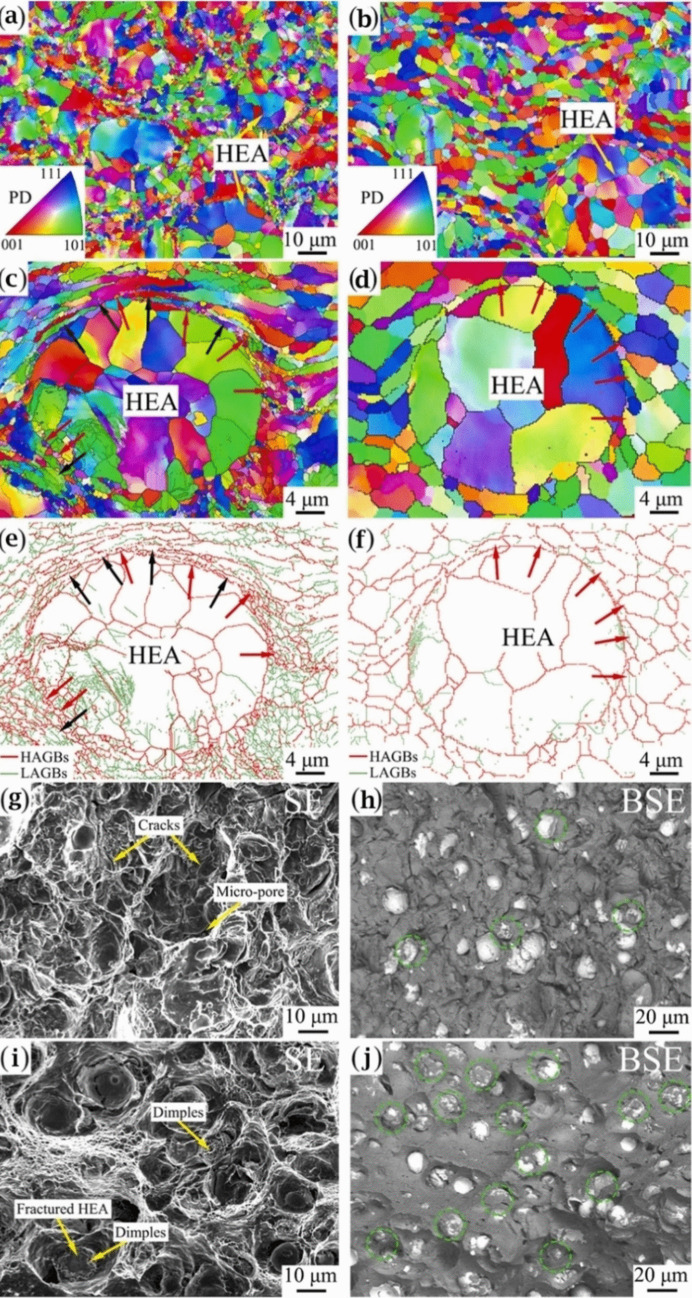
The EBSD images of the (**a**, **c**, **e**) CSed sample (6061Al/CoCrFeNi) and (**b**, **d**, **f**) FSPed sample; (a–d) IPF; (e, f) grain boundaries. SEM micrographs of fracture surfaces of tensile samples observed in the (g, h) CSed sample and (i, j) FSPed sample [164] (reprinted with permission from Elsevier B.V., Copyright © 2022)

A new study reported the development of a novel AMC reinforced by CoCrFeNi HEA using cold spray (CS) deposition technology for the first time [164]. The samples were further improved by using subsequent FSP, which contributed to a significant enhancement of their mechanical and anti-wear properties as well as the elimination of cold spray defects such as micropores and microcracks in the stir zone. Figure 18b–e demonstrate micropores in cold sprayed samples, along with the well-distributed reinforcement particles, which mostly retained their spherical morphology with an average size of 21 µm. Some HEA particles collided with each other and caused severe jetting at the boundaries of the particles (see Fig. 16d). SEM images of FSPed samples clearly illustrate the micropores and cold spray defects’ removal. Additionally, a few HEA particles were fragmented and further distributed throughout the matrix, resulting in the formation of white-band structures in the macro profiles (Fig. 16a). According to the EBDS results (Fig. 19a–f), many fine grains were accumulated in the area close to the HEAs in the matrix after cold spraying. Subsequent sintering made this heterogeneous grain texture more homogeneous. Furthermore, the average grain size slightly increased after sintering, which was due to the coarsening of recrystallized grains provided by the high heat input of SPS. As suggested by other studies, HEAs as reinforcement induce recrystallization by particle-stimulated nucleation, which inhibits grain growth [158, 165]. However, sintering provides enough thermal energy for grain growth to occur. A thicker interfacial layer was formed in the FSPed samples compared to cold sprayed series, which can be attributed to the high FSP temperature and longer duration. Mechanical tests showed that the microhardness greatly increased in the FSPed samples (from 74 to 141 HV) due to the homogenization and densification of the microstructure under the effect of FSP. This is consistent with other studies on other cold sprayed composites modified by FSP [166–168]. The UTS and elongation of FSPed samples increased by 60% and 130%, respectively. Fractography analysis of the samples revealed that the fracture surface of the cold sprayed samples exhibited a brittle nature due to the high work hardening of the deposit and the presence of micropores and cracks. On the other hand, the fracture surface of the FSPed samples displayed small dimples all over the matrix indicating a ductile nature of fracture in these samples (Fig. 19g–j). A summary of the preparation methods and the physico-mechanical properties of MMCs reinforced with HEA particles is presented in Table 3.

**Table 3:** Summary of the preparation methods and the obtained properties of MMCs reinforced with HEA particles

Matrix/reinforcement	Fabrication method	Physico-mechanical properties	Remarks	Ref
2024 Al alloy/ CoCrFeMnNi 7 vol.%	SPS (823 K, 40 MPa)	Hardness: 135.48 HV_0.05_ Young’s modulus: 330–70 GPa	Hardness was increased by 63.7% compared to Al matrix. The average value of hardness of composite was found to be higher than the theoretical value (94 HV_0.5_) calculated from the rule of mixture owing to the presence of ID layer, which boosted the matrix hardness and gradually increased the hardness from the matrix to reinforcement	[89]
Al/CuZrNiAlTiW 10, 20, 30 vol.%	SPS (773 K, 80 MPa, 30 min)	Relative density: 98.46% Corrosion potential: − 1.04 ± 0.03 V Hardness: 280–370 HV UTS: 344 ± 2 MPa Ultimate strain: 7.23%	Samples with 30% reinforcement demonstrated the highest corrosion resistance and hardness Samples with 10% reinforcement achieved the highest plasticity among the reinforced samples	[131]
Al/AlCoCrFeNi 5 vol.%	SPS (540–600 °C, 10 min, 6 MPa)	Yield strength (sintered at 580 °C): 137 MPa Compressive strain (at yield point, for samples sintered at 540 °C): 36%	Yield strength of composite improved from 96 to 137 MPa by realization of transition layer between reinforcements and the matrix (42% improvement). Samples sintered at 580 °C did not develop any macroscopic crack up to maximum tested strain (50% compressive strain)	[135]
Al_65_Cu_16.5_Ti_18.5_ amorphous alloy/Al0.6CoCrFeNi 50 vol.%	SPS (823 K, 400 MPa)	UTS: 3120 ± 80 MPa	About 80% increase in UTS	[141]
5052 Al/Al0.6CoCrFeNi 7 vol.%	Vacuum HP (823 K, 30 MPa, 60 min)	Young’s modulus: 80 GPa Hardness: 1.81 GPa	Highest values of recorded hardness and Young modulus were recorded for samples heat treated at 500 °C for 12 h. Hardness was increased by 14.7–26.6% under different heat treatment conditions	[142]
2024 Al/CoNiFeCrAl_0.6_Ti_0.4_ 7.5, 15, 30 vol.%	HP (480 °C, 30 min, 150 MPa) Hot extrusion (450 °C, ratio 10:1) Further T6 heat treatment	Yield strength (7.5% samples): 419 ± 12 MPa Fracture strain (7.5% samples): 8%	Samples with 7.5% reinforcement showed the best results in terms of yield strength and strain: 112 MPa higher strength compared to unreinforced matrix (i.e., about 20% improvement) while fracture strain slightly dropped compared to the unreinforced sample (8% for the reinforced sample compared to 12% for unreinforced sample)	[143]
Cu/Al0.3CoCrFeNi 5 wt.%	Mechanical alloying and sintering (950 °C, 30 min, vacuum)	Wear rate: 0.09 mgr/m	Interfacial copper layer of reinforcement applied by means of ball milling significantly improved wear resistance of the samples	[144]
5083 Al/FeCoNiCrAl	FSP (FSP tool of 1200 rpm, processing speed of 45 mm/min)	Maximum microhardness: 158 HV Friction coefficient: 0.4233 Wear rate: 1.6 × 10^−6^ mm^3^/N.m	Best results indicated that by addition of reinforcements microhardness almost doubled after 5 passes of FSP By introduction of reinforcements into the sample, friction coefficient was decreased by almost 40% after 5 passes of FSP Reinforced samples showed 50% higher wear resistance compared to the plain samples	[147]
AA5083-H111 Al/Al_0.8_CoCrFeNi 3.8 vol.%	FSP (5 passes, rotational velocity of 800 rpm, processing speed of 50 mm/min)	Yield strength: 200.5 ± 2.7 MPa UTS: 371 ± 1.7 MPa Fracture strain: 18.8 ± 0.5% Maximum microhardness: 125.7 HV_0.2_ Hardness: 1.07 GPa Young’s modulus: 102.4 GPa	Yield strength and UTS were increased by 42% and 22%, respectively, and fracture strain was decreased by 30% due to the addition of reinforcement 56.1% improvement in microhardness compared to plain FSPed samples Hardness was increased by 37.2% Young’s modulus was increased by 16.2%	[148]
5083 Al/AlCoCrFeNi2.5	Underwater FSP (5 passes of FSP)	Hardness: 135 HV Young’s modulus: 100.06 GPa Plasticity index: 88.0% Friction coefficient: 0.292 Wear loss: 2.0 mgr Wear rate: 7.55 × 10^−4^ mm^3^ N^−1^ m^−1^	68.8% increase in hardness compared to as-received Al plates Plasticity index decreased compared to as-received samples, showing that addition of HEA particles had stiffened the matrix Wear loss decreased by 46.6% by the addition of reinforcements	[158]
AA5083 Al–Mg/CoCrFeNi 12 vol.%	Friction deposition (Al–Mg alloy consumable rods; process parameters: 800 rpm spindle, 8 kN friction force, and 25 s friction time)	0.2% tensile stress: 280 ± 5 MPa UTS: 395 ± 7 MPa Tensile elongation: 5 ± 2% Bulk hardness: 54 ± 3 HRB	0.2% tensile stress was improved by 50%, UTS was increased by 32% while tensile elongation was decreased by 58% as the result of addition of HEAs to the matrix Bulk hardness was increased by 80%	[161]
6061 Al/CoNiFeAl_0.4_Ti_0.6_Cr_0.5_ 7.5 vol.%	HP (490 °C, 120 MPa) Hot extrusion (450 °C, ratio 10:1)	Hardness: 94–97.5 HB Yield strength: 326–350 MPa UTS: 375–380 MPa Ultimate strain: 8–5%	Different groups of samples were obtained at different ball-milling times of 10, 20, and 40 h; the samples prepared at 10-h ball-milling time showed best results in UTS and fracture strain which was 378 MPa and 8.4% compared to 310 MPa and 11% for pure matrix	[162]
Cu/ AlCoNiCrFe 10, 20 wt.%	Sintering (1073 K, 70 MPa, 30 min)	Yield strength: 240 MPa, 330 MPa UTS: 500 MPa, 475 MPa Ultimate strain: 7.5%, 24%	Samples with 10% and 20% reinforcement showed 160% and 220% improvement in yield strength compared to the pure Cu matrix, while ultimate strain only slightly decreased from 30% for Cu matrix to 25% and 17.5% for samples with 10% and 20% reinforcement, respectively	[163]
AA5083 Al–Mg/AlCoCrFeNi 5, 10, and 15 vol.%	Cooling-assisted FSP (rotation speed of 1400 rpm, processing speed of 40 inch/min)	Microhardness (15 vol.% HEA content): 136 HV Wear rate: 8.5 × 10^−5^ mm^3^/m^−1^ Wear loss: 1.6 mg	In samples with 15 vol.% HEA, hardness was increased by 65.9%, wear rate was decreased by 43.0%, and wear loss was reduced by 57.8%	[169]

### Other metals

Four main groups of metallic reinforcements have been proposed and studied to improve the physico-mechanical characteristics of the MMCs, by far. In this section, additional reports on introducing unique secondary particles into the metal matrix to boost mechanical properties such as fracture toughness, hardness, and plastic strain are covered. To demonstrate their outstanding performance in reinforcing the matrix, we can refer to their development in BMGs, which have recently shown remarkable results in the studies performed in recent years. Since thanks to the shear bands (SBs) generated under glass transition temperature, *T*_g_, macroscopic plastic deformation is facilitated in BMGs. On the other hand, the failure of the BMGs subjected to the uniaxial tension is the consequence of nucleation and propagation of SBs, which can provide insignificant ductility [170]. Introducing a microstructure being able to resist the propagation of SBs, like a composite, can provide ductility, which can boost reliability and toughness [171]. To address the room-temperature brittleness of BMGs, bulk metallic glass matrix composites (BMGMCs) have been developed due to their specific structure that can be strengthened by both in situ produced secondary phases or ex situ directly introduced secondary particles [172].

#### In situ* and *ex situ* BMGMCs*

Two methods can be introduced for manufacturing BMGMCs according to the formation of the second phase in the crystal. Regarding the in situ BMGMCs, the crystalline second phase is produced during manufacturing, and due to the lack of surface oxides and low interface energy between the secondary phase and the matrix, it tends to strongly connect with the glassy matrix, which is the main advantage of in situ BMGMC formation [172, 173]. However, the ex situ BMGMCs are made by adding high-melting-temperature reinforcements such as Ti, W, Ta, and ceramic particles, which can cause the formation of multiple SBs during homogeneous plastic deformation in the case of homogeneous dispersion [173]. Therefore, the ex situ approach necessitates a good match between the BMG matrix and the secondary crystalline phase; otherwise, impurities, oxides, and voids at the BMG matrix/crystalline phase interface would severely degrade the mechanical performance of BMGMCs [174]. In the following, the influence of metallic secondary phases as reinforcements in BMGMCs will be discussed.

#### Ti-reinforced BMGMCs

**Fig. 20:**
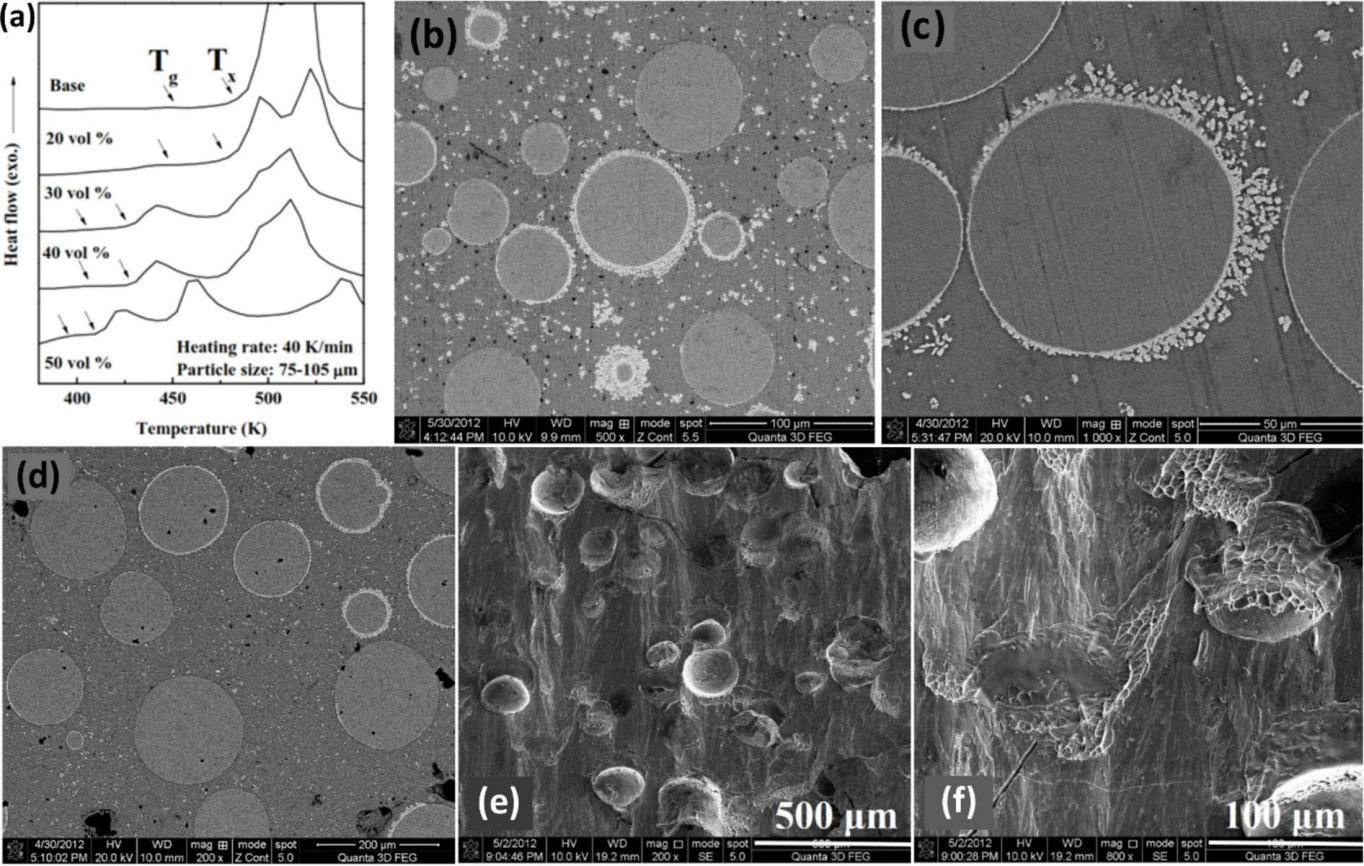
(**a**) Representative differential scanning calorimetry scans of Mg_60_Zn_35_Ca_5_-based BMGMC samples containing different volume fractions of 75–105-μm-sized Ti particles. Back‐scatter electron images of the developed brittle TiZn intermetallic compound in Mg_60_Zn_35_Ca_5_-based BMGMC samples containing different particle sizes of 50% Ti particles: (**b**) 20–75 μm; (**c**) 75–105 μm; (**d**) 105–130 μm. SEM micrographs of (**e**) fracture surface of specimen after compression test for the Mg_60_Zn_35_Ca_5_ BMGMC containing 50 vol.% Ti particles with sizes of 75–105 μm; (**f**) enlarged image of (**e**), some areas display vein pattern mixed with melting trace around Ti particles [175] (reprinted with permission from MDPI, Copyright © 2016)

Most Mg-based crystalline alloys exhibit extremely fast corrosion and degradation rates, which can be addressed by exploiting BMGs such as MgZnCa presenting a much lower corrosion rate. However, monolithic BMGs are exceptionally brittle, the characteristic that places adverse implication on the toughness of metallic composites. To tackle this challenge, BMGs require to be composited with a secondary phase to promote their strength as well as plasticity [175]. Since Ti metal is well known for its immiscible interaction with the Mg matrix, Ti particles were chosen by Wong et al. [175] to be added to the Mg_60_Zn_35_Ca_5_-based BMGs to increase their plasticity with different volume fractions of Ti (20, 30, 40, and 50 vol.%), all of which were prepared by induction melting. The illustrative differential scanning calorimetry scans of the Mg_60_Zn_35_Ca_5_-based BMGMCs in Fig. 20a demonstrated that by increasing the volume percentage of Ti particles, both *T*_g_ and crystallization temperature (*T*_x_) decreased. The multiplication of heterogeneous nucleation sites from the interfaces between the Mg-based glassy matrix and Ti particles might be the cause for such modifications [175].

The fracture strength of Mg_60_Zn_35_Ca_5_-based composites was significantly enhanced by increasing the volume percentage of ex situ added Ti particles. However, the majority of samples exhibited failure strains of less than 3%. This can be attributed to the formation of a brittle TiZn intermetallic compound that developed around the interface of the Ti particle and amorphous matrix, which led to embrittlement of the composites, as illustrated in Fig. 20b–d. Due to the limited interface adhesion of Ti dispersoids to the matrix, it is believed that they are unable to entirely prevent the propagation of SB. This phenomenon is demonstrated in Fig. 20e, where Ti particles were observed to have been torn away from the matrix. In addition, Fig. 20f clearly indicates the melting trace and vein pattern [175]. It is well known that as SB planes are locally melted by heat or free volume created during deformation, vein patterns are often observed on the fracture surfaces of amorphous alloys [176]. Large vein patterns indicate rapid SB propagation due to the significant plastic movement before local melting and solidification, whereas small vein patterns suggest slow SB propagation [177]. Thus, the adhesion ability of the interface between Ti particles and the amorphous matrix is crucial to the performance of Mg-based BMGMCs. Overall, the dispersion of Ti particles in Mg_60_Zn_35_Ca_5_ BMG alloys is considered a promising strategy for enhancing their plasticity and yield strength [175].

**Fig. 21:**
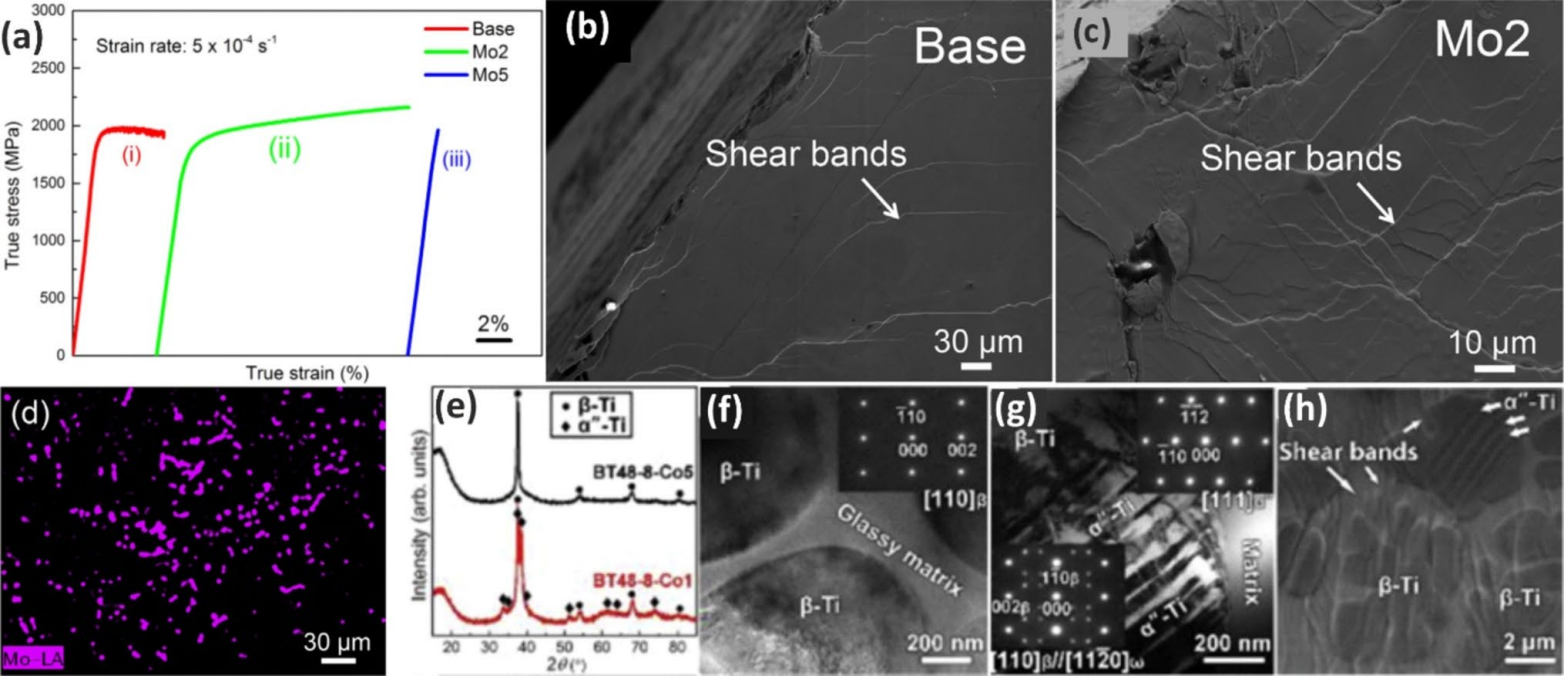
(**a**) Compressive true stress–strain curves for (i) base, (ii) Mo2, and (iii) Mo5; SEM images for as-fractured samples: (**b**) base; (**c**) Mo2; (**d**) element mapping of Mo at the fracture surface of Mo2 [178] (reprinted with permission from Elsevier B.V., Copyright © 2017). (**e**) XRD patterns obtained from the fractured Co5 and Co1 specimens. (**f**) TEM image obtained from the fractured Co5 with an SAED pattern of β-Ti. Note the absence of ω-Ti or martensitic phases in dendrites of Co5. (**g**) TEM image of fractured Co1 with SAED patterns of β-Ti and a″-Ti. (**h**) SEM image of fractured Co1, illustrating that a relatively fewer number of shear bands penetrate β-Ti crystals that contain a″-Ti plates [170] (reprinted with permission from Elsevier B.V., Copyright © 2019)

In situ development of β-Ti reinforced Ti-based BMGMCs has demonstrated excellent plasticity during deformation [178]. However, since β-Ti is metastable at room temperature, it is necessary to add specific alloying elements (such as Mo, V, Nb, and Ta) to the parent alloys in order to stabilize the β-Ti phase [179]. The microstructures and mechanical properties of Mo-microalloyed BMGMCs were investigated by Guo et al. [178] with different amounts of Mo (0, 2, 5 at.%, denoted as base, Mo2, Mo5). The results showed that in both compression and bending tests, the β-Ti reinforced BMGMCs outperformed the monolithic glassy equivalent in terms of plasticity and fracture strength. The soft and ductile β-Ti phase distribution appeared to effectively obstruct the quick propagation of the primary SB, resulting in multiple SBs. As shown in Fig. 21a, while the monolithic BMG had a plastic strain of 3% and a fracture strength of 2050 MPa, the β-Ti Mo2 BMGMC exhibited significantly higher plastic strain (up to 13.4%) and fracture strength (up to 2160 MPa). Furthermore, the Mo2 BMGMC displayed work-hardening behavior after yielding, which was not observed in the base BMGMC. This could possibly be attributed to the shear-induced local dilatation or the increased free volume [180].

The significance of variations in hardness between the metallic glass and the secondary phase has been demonstrated in the toughening of metallic glass through the introduction of a secondary phase [178]. During loading, yielding and deformation in the secondary phase increased, while they were restricted by the surrounding matrix when the secondary phase was softer than the matrix [181]. The fracture surfaces of both the base and Mo2 were studied by SEM to explore the reinforcing processes, as illustrated in Fig. 21b–d. The flexibility of the as-fractured monolithic glassy base sample was confirmed by the presence of SBs on the lateral surface (Fig. 21b). Multiplied SBs, on the other hand, appeared on the lateral surfaces of the as-fractured Mo2 (Fig. 21c), indicating that the propagation of the single SB was affected by the interfaces between the softer β-Ti and the glassy matrix, causing the SB to deflect, branch, or multiply. The element mapping of Mo is shown in Fig. 21d, indicating significant interactions between the softer β-Ti phase and the matrix. Moreover, it appears that the β-Ti phase undergoes yielding and deformation following elastic deformation of both β-Ti and the glassy matrix, leading to work-hardening behavior [178].

Yet, among the numerous reinforcing phases, in situ precipitation of crystalline dendritic phases into the amorphous matrix during solidification seems to be the most promising [182]. Zr/Ti-based BMGMCs containing β-Zr/Ti dendrites have been explored to fabricate a material with both high strength and ductility [170]. Extensive research into the plastic deformation processes in β-type BMGMCs showed that the crystalline β-Zr/Ti phase yields at a substantially lower stress than the amorphous matrix [183]. The yield strength of the amorphous matrix sets the upper limit for the strength of a BMGMC since it is the harder of the two components. In the early stages of composite deformation, plastic deformation in the dendrites and elastic deformation in the matrix are compatible due to the matrix's ability to withstand elastic stresses (up to 2%). Further straining increases strain incompatibility at the matrix–dendrite interface, promoting the formation of SBs in the matrix. In this situation, neighboring dendrites in BMGMCs might stop the propagation of SBs, which is in turn influenced by a variety of parameters, e.g., size, shape, and relative crystallographic orientation [170]. The researchers also investigated how the sensitivity of β-Ti to stress-induced phase transformation during tensile deformation affects the mechanical behavior of BMGMCs. Their findings revealed that the stress-induced martensitic transformation of the dendritic Ti phase from β to α″ (the martensitic phase in Ti) enhanced the composite's resistance to SB propagation and thus provided significant strain hardening capacity. Additionally, increasing the number of converted particles by decreasing the metastability of β-Ti further improved the mechanical properties of the BMGMCs. However, when the Co content of the alloy was increased beyond 5 at.%, the β-Ti phase not only became more resistant to phase transformation but also became softer, as demonstrated by tests where the Co content varied from 1 to 5 at.%. By lowering the Co concentration to less than 1 at.%, on the other hand, the β-Ti phase decomposed quickly to α-Ti and numerous other brittle intermetallics [184]. TEM images of specimens with different Co contents (1 and 5 at.%, denoted as Co1 and Co5, respectively) subjected to tensile testing are presented in Fig. 21e–h. Nanoindentation studies revealed that dendrites could effectively prevent SB propagation under certain conditions: they must be (i) favorably oriented for shear transformation to occur and (ii) large enough to absorb the strain energy generated by SB propagation, preventing the SB from transmitting across the dendrite. It is important to note that not all metastable β-Ti dendrites undergo martensitic transformation during deformation. The efficiency of dendrites against SB propagation would be insignificant if most dendrites remained untransformed, resulting in early commencement of necking. Additionally, reducing the inter-dendritic spacing can minimize the average distance that an SB travels before encountering another dendrite, increasing the number of barriers and enhancing the overall SB resistance of the BMGMCs [185]. When the influence of dendrite size and inter-dendritic spacing is considered, the involvement of Co in changing the metastability of the β phase is critical because it eliminates the requirement for volume fraction (V_d_) > 70%, which is the major reason for the low strength of the composite. The objective of overcoming the strength–ductility conflict in BMGMCs would have remained elusive without this adjustment [170].

#### Ta-reinforced BMGMCs

**Fig. 22:**
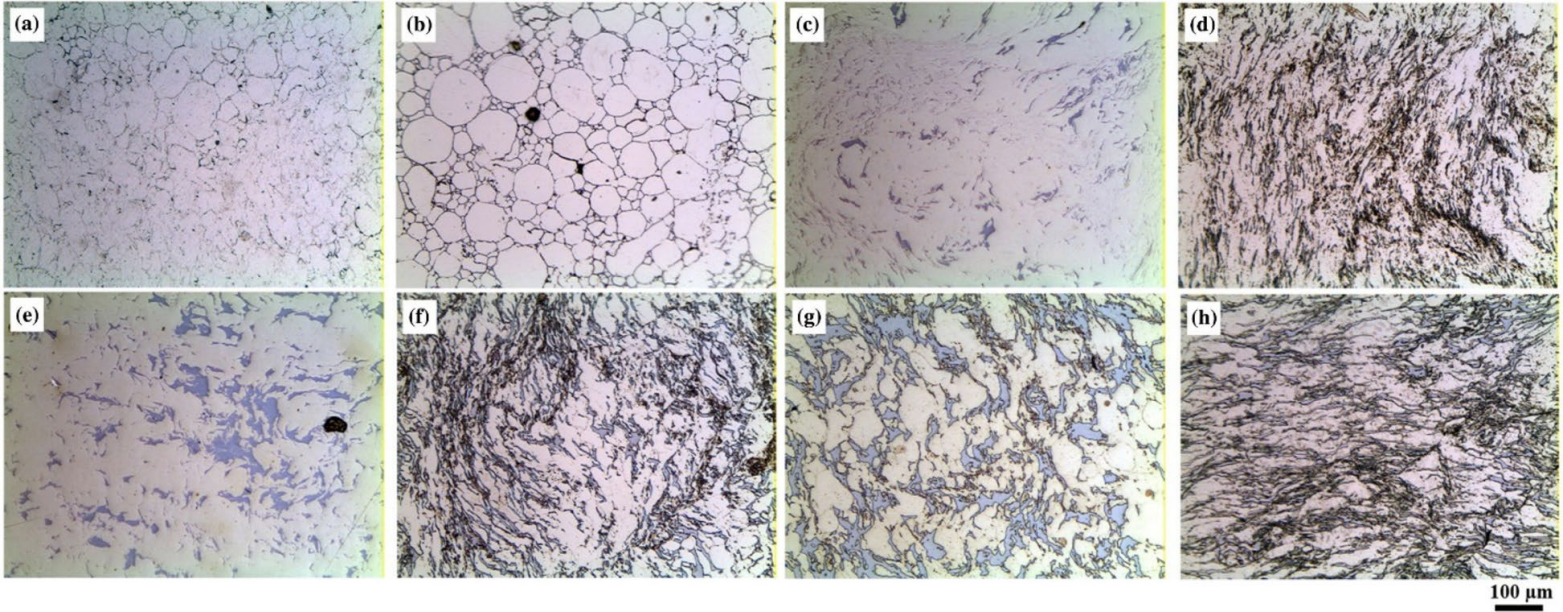
OM micrographs from BMGC disks fabricated by HPT with 2 turns at room temperature (a, c, e, g) and at 200℃ (b, d, f, h) containing various amounts of Ta: (**a**, **b**) 0 (monolithic BMG), (**c**, **d**) 10 vol.%, (**e**, **f**) 20 vol.%, and (**g**, **h**) 30 vol.%. The micrographs show the microstructure near the middle regions [187] (reprinted with permission from Springer Nature, Copyright © 2018)

It is widely recognized that melting a second phase into an amorphous matrix can induce significant changes in the composition, structure, glass-forming ability, crystallization behavior, and other properties of the resulting glassy alloy. Liu et al. [186] reported that increasing the Ta concentration in the alloy led to the formation of a homogeneous glassy phase at low Ta concentrations. Further increment of Ta concentration increased both glass transition temperature (*T*_g_) and the crystallization onset temperature (*T*_x_). Structural and thermal analysis revealed that incorporating Ta particles into Cu_54_Zr_22_Ti_18_Ni_6_ powder and subsequent consolidation using high-pressure torsion (HPT) for the production of BMGMC disks failed to alter the amorphous nature of the BMG matrix [187]. This is due to the fact that the Ta particles were only physically combined with the BMG powder before being distorted mechanically during HPT. However, the addition of Ta powder had an inevitable influence on the consolidation behavior and microstructure of the monolithic BMG sample. Their inclusion indeed enhanced the susceptibility of monolithic BMG powders to plastic deformation, as demonstrated in Fig. 22e–h. As a result, the increased flexibility of BMGMC powder compared to Ta-free powder hindered the significant localized internal stresses formed in the HPT setup's limited shape. Ta particles were shown to limit the propagation of SB and cracks, which improved the mechanical characteristics of BMGMCs, as can be seen in Table 4. Specifically, the BMGMC sample with a Ta content of 30 vol.% exhibited the most favorable combination of fracture load and fracture deflection [187].

**Table 4:** Summary of the preparation methods and the obtained properties of MMCs reinforced with intermetallic particles

Matrix	Fabrication method and condition	Physico-mechanical properties	Remarks	Ref
Al/AlCuLi	Microwave sintering (powder of size ∼5–15 μm, ball mill at 200 rpm for 2 h, compacted at 97 bar, sintered at ∼550 °C, 900 W, 2.45 GHz, homogenized at 400 °C for 1 h)	For AlCuLi 15 vol.%: Hardness: ~10.2 GPa Young's modulus: ~ 112 GPa Ultimate compression strength: ~ 453 MPa Failure strain: 6.4% Ultimate tensile strength: ~167 MPa Yield strength: ~144 MPa Elongation: 6.9%	Significant improvement of the room-temperature compressive/tensile behavior of the composite because of good reinforcement–matrix interface Improved thermal stability	[24]
TiZrCuCoBe/β-Ti phase	Arc melting (Ar atmosphere, remelted 4 times)	Yield stress: 1397 ± 15 MPa Strain: 1.93 ± 0.05%	Enhanced resistance to shear band propagation by stress-induced martensitic transformation of the dendritic Ti phase Imparting significant strain hardening capability to the composite	[170]
MgCuAgGd/Ti (5 at.%)	Dealloying method by arc melting (Mg and Ag in a carbon crucible in a He atmosphere at 1073 K for 3 min)	Fracture strength: ~ 920 MPa Fracture stress: 331 MPa Fracture strain: 0.42%	Better mechanical properties, including higher fracture strength and larger plastic strain, than both their monolithic glassy counterparts and BMGMCs by the ex situ dispersing or conventional methods	[172]
ZrCuAlNi/Ta (8 at.%)	Dealloying method by arc melting (Ta–Zr precursor with Cu, Al, Ni pieces in a BN crucible in a He atmosphere at 1273 K for 3 min)	Fracture strength: 1880 MPa Fracture stress: 1614 MPa Fracture strain: 3.4%	More interfaces to improve further the plasticity due to finer size of dispersoids; less composition fluctuation of the matrix for the BMGMCs	
NiCuZrTiSiSn/B2 phase	Arc melting (Ar atmosphere, ingots remelted at least 5 times)	Fracture strength (B2 10 vol.%): ~2.5 GPa	With further increasing Ti content up to 24 at.%, the B2 phase was introduced in the amorphous matrix, which exhibited higher hardness and modulus than those of amorphous matrix as well as effective stress accommodation up to the higher stress level than the yield strength of amorphous matrix	[173]
ZrTiBeCu/Ti6Al4V	Thermoplastic forming (ultrasonic vibration frequency of 20 kHz, forming temperature 643 K, incubation time of 300 min)	Fracture toughness: 213 MPa.m^1/2^ (170% increment compared with monolithic BMG) Bending strength: 1030 MPa	Enhanced fracture toughness for the addition of Ti6Al4V frame	[174]
MgZnCa/Ti (20, 30, 40, and 50 vol.%)	Induction melting (Ar atmosphere)	Fracture strength: 1190 MPa of (50 vol.% 25–75 nm)	Both the fracture toughness and the compression failure strain increased with a decreasing mean free path of the shear band Overall performance was strongly dependent on the adhesion ability between the interface of the Ti particle/amorphous matrix	[175]
ZrTiCuNiBe/W	Induction melting (tungsten particles size 700–900 μm, vacuum-dried at 300 °C for 20 min, heated to 780 °C, pre-alloyed material melted at 850 °C under 2 × 10^−3^ Pa)	Compressive strength (10 min infiltrating time): 2030 MPa Compression strain (10 min infiltrating time): 44%	Improving the compressive properties by increasing the infiltrating time, which results in the increase in the number of SBs, and decreasing the gap width between the shear bands Tiny crystals are prone to generate at the boundaries between the tungsten particles and amorphous matrix and their size increased with the infiltrating time	[176]
Mo-microalloyed TiZrCuPdSn/β-Ti phase	Arc melting (Ar atmosphere)	13.4% greater plastic strain Fracture strength: 2160 MPa β-Ti phase hardness: 4068 ± 60 MPa (vs. 4150 ± 62 MPa of matrix)	Better plasticity and fracture strength than the monolithic BMG under both compression and bending	[178]
CuZrTiNi/Ta (10–30 vol.%)	HPT (6 GPa, at RT or 200 °C, lower anvil rotated at a constant speed of 1 rpm of 1 to 3 turns)	Slope of the load–deflection curve at RT (10 vol.%): 453 N mm^−1^ compared to 371 N mm^−1^ for monolithic BMG	Improved consolidation behavior by which high-density crack-/debonding-free compacts were achieved Improvement in the plastic deformability of particles by increasing the volume fraction of Ta or the HPT temperature Higher hardness as well as higher fracture load and deflection in small punch test compared with the monolithic BMG specimens	[187]
ZrNiCuTaAl/Ta (8 at.%)	SLM (100–300 W, spot diameter of 80 mm, scanning direction of 90°, oxygen content below 100 ppm, layer thickness of 60 mm, *v* = 1200–2000 mm/s)	Fracture strength: 1.9 GPa Plastic strain: 2.15% Notched fracture toughness (*K*_q_): 60 MPa.m^1/2^	High fraction of amorphous phase (> 90%) was obtained Enhancement of plasticity and fracture toughness while maintaining high strength by precipitation of Ta particles	[188]
ZrCuNiAl/Ta (7, 9, 10 at.%)	Arc melting	True plastic strain (Ta 10 at.%): 43.5% (6.25 times higher than monolithic BMG) Tensile plastic strain: 2%	Higher fracture strength and larger plastic strain Strong bonding between Ta-rich particles and glassy matrix without any undesired layers	[189]
ZrCuNiAlTa/Ta (4, 5, 6, 8 at.%)	Arc melting (argon atmosphere)	Fracture strength (Ta 4 at.%): 2.70 GPa Fracture strength (Ta 8 at.%): 1.20 GPa	Significant decrease in fracture strength with the increase in volume fraction of Ta particles	[190]
CuZr/Ta (8 at.%)	Arc melting (ingot was flipped and re-melted at least 4 times)	–	Enhanced thermal stability by the addition of Ta due to having higher *T*_g_ and *T*_x_ than the monolithic BMG	[191]
FeCrMoYCB/Ni (up to 10 vol.%)	SPS (powder mixing by ball milling, sintering at 600 °C, 50 MPa, heating rate 50 °C/min, and holding time of 10 min)	Microhardness (Ni 2.5 vol.%): 1150 HV Microhardness (Ni 10 vol.%): 890 HV	BMGMCs with lower reinforcement content (2.5 to 5 vol.% Ni) are promising in applications where optimum corrosion and wear performance is desired	[192]
CoCuFeNi/W	Arc melting (argon atmosphere, solidified in a water-cooled Cu crucible)	For W 15 vol.%: Yield strength: 354 MPa Ultimate tensile strength: 570 MPa	Increase in the strength of CoCuFeNi HEA while retaining good ductility The interfaces between FCC matrix and W particles were semi-coherent	[193]

**Fig. 23:**
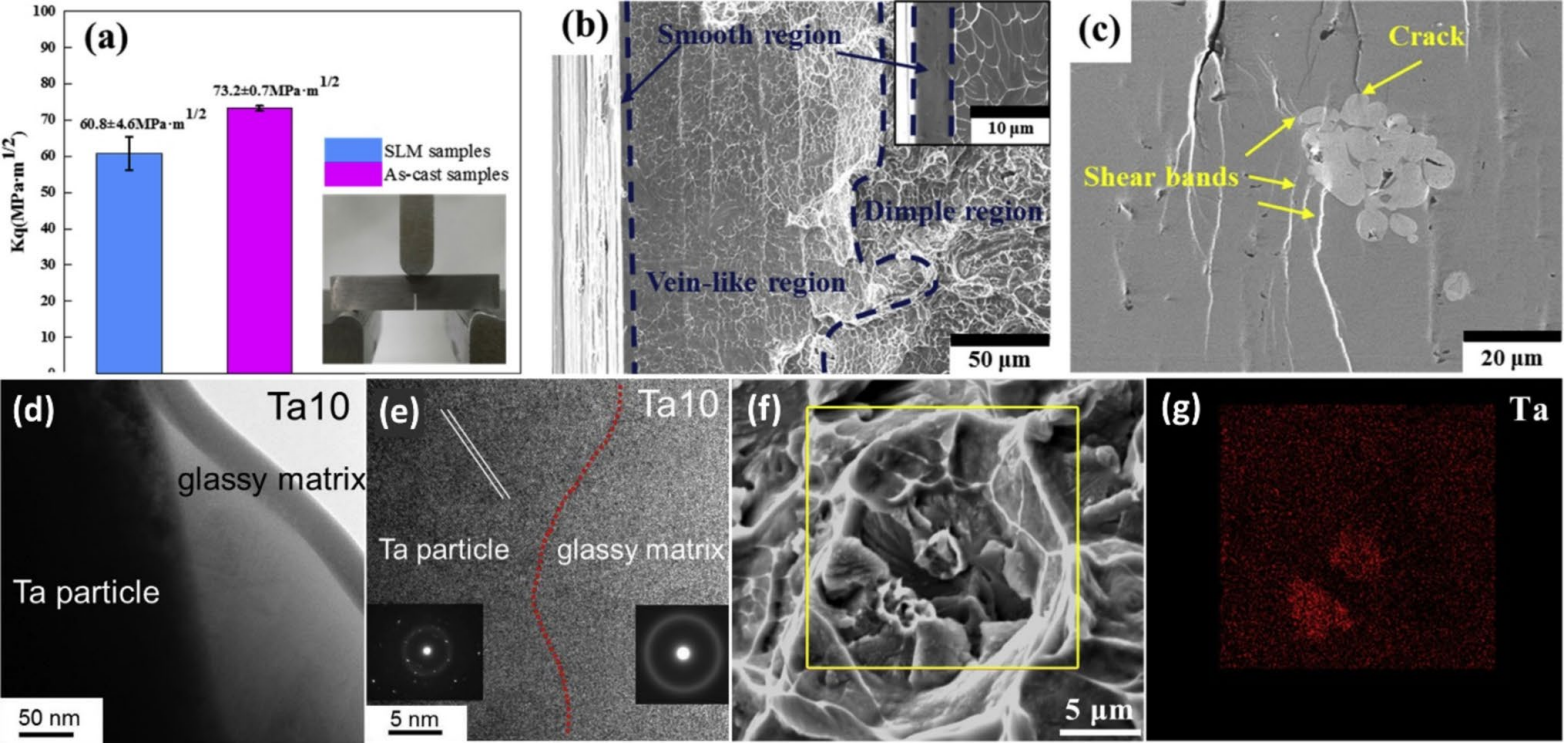
(**a**) Fracture toughness of Zr-based BMG composite samples fabricated by LPBF and casting, the inset image shows a fracture toughness sample; (**b**) fracture surface of LPBF Zr-based BMG composite sample after fracture toughness test; (**c**) shows the shear bands and crack near Ta precipitation on the side cross-section of a LPBF sample [188] (reprinted with permission from Elsevier B.V., Copyright © 2019). (**d**) Bright-field TEM and (**e**) HRTEM images of a composite reinforced by 10 at.% Ta. The selected area diffraction patterns of both Ta-rich particle and the glassy matrix are inserted in (e) [189] (reprinted with permission from Elsevier B.V., Copyright © 2019). (**f**, **g**) High magnification image of the dimple on fracture surface of notched Ta8 BMGC, as well as the corresponding Ta Lα map [190] (reprinted with permission from Elsevier B.V., Copyright © 2019)

Additive manufacturing technique based on laser powder bed fusion (LPBF) was implemented in a study for fabrication of large-scale BMGs and components with complex geometries [188]. This technique also offers a possible way to improve Zr-based BMG’s mechanical properties by ex situ addition of ductile particles such as Ta [188]. This is particularly relevant since additive manufactured BMGs exhibit limited plasticity, even under compressive loading. As shown in Fig. 23a, both toughness and plastic strain of the additive manufactured BMG composite were comparable to the as-cast BMG composite. SEM images from the fracture surface, as shown in Fig. 23b, clearly show three distinct regions: smooth (see the inset for a magnified view), vein-like pattern region, and dimple region, all of which are consistent with the typical morphology of as-cast BMGs reported in the literature [194]. The smooth region is thought to be created by SB shear-off [195], which is an indicator of the SB’s motion at early stages. On the other hand, adiabatic slippage of the major SBs is linked to the vein pattern area [195], while the dimple pattern on the fracture surface is associated with rapid crack propagation. Figure 23c illustrates the two key roles played by Ta particles: generating numerous SBs and hindering crack propagation, as evidenced by the arrows. These aspects are thought to contribute to the enhanced toughness observed in the composites, compared to the previously reported additive manufactured single phase BMGs [188]. Furthermore, the interfaces between the glassy matrix Zr_55_Cu_30_Ni_5_Al_10_ and in situ ductile Ta-rich particles were studied by Guo et al. [189]. TEM images of a sample reinforced with 10 at.% Ta, without any oxide or intermetallic compound layers (Fig. 23d, e), demonstrate a highly distinct interface between the Ta-rich phase and the amorphous phase of the BMG. Additionally, selected area diffraction patterns (SADs) of each region show a gradient change from the crystalline ordering fringe of the Ta-rich phase to the maze typical of the amorphous phase in Fig. 23e. Based on these findings, it can be inferred that the Ta-rich phase and the matrix have a strong interface, which enhances the composite's ductility. The composites exhibit higher plastic strain with increasing volume fraction of Ta-rich particles. For example, when the Ta content was increased from 7 to 10%, the true plastic strain of the composite in compression testing increased by more than 2 times to 43.5%, while the true fracture stress decreased by only 4.5% to 1993 MPa [189].

Recently, it has been proposed that the fracture resistance of BMGs stems from the interplay between shear banding and cavitation/void nucleation [196]. While the mechanism of shear banding is well established, there is still limited understanding of the deformation process in BMGs guided by cavitation/void nucleation. Previous studies have shown the presence of nanoscale cavities in shear bands, which are typically associated with brittle fracture [197]. It was found by Pan et al. [190] that an increase in the volume fraction of Ta particles in notched BMGMCs led to a significant reduction in fracture strength. For example, the fracture strength decreased from 2.70 GPa in the monolithic Zr_58_Cu_15.6_Ni_12.4_Al_10_Ta_4_ BMG to 1.20 GPa in the Zr_54_Cu_15.6_Ni_12.4_Al_10_Ta_8_ alloy with 3.3% Ta particles. According to the findings, in the triaxial stress condition, shear banding may be effectively controlled in notched BMGs and BMGMCs [198], and cavitation may transform to the dominant deformation mechanism at the first stage of failure [199]. In notched monolithic BMGs, decohesion developed at nanoscale soft areas, which made the decohesion difficult and resulted in high fracture strength and decohesion stress. In notched BMGMC, however, the presence of an interface between Ta particles and the amorphous matrix might facilitate void nucleation and coalescence, leading to reduced fracture strength. A Ta Lα map was studied on a specific dimple on the fracture surface of the alloy with 8% Ta, as illustrated in Fig. 23f and g. Regardless of the effect of height, the Ta element was concentrated mostly near the center of the dimple, which corresponded to the position of protrusions. Since the center of the dimple was where voids began to form or nucleate, voids were likely to form at the interfaces between the Ta particles and the amorphous matrix [190]. The nucleation and coalescence of voids at the interface of Ta particles and the amorphous matrix can be attributed to two factors: (i) the strength of the interface between Ta particles and matrix is significantly weaker than the atomic bonding in monolithic BMGs, resulting in a lower BMGMC decohesion stress; and (ii) during deformation, stress concentration at the interface may be caused by incompatibility between Ta particles and the amorphous matrix. The Young's modulus of Ta was 186 GPa, which is more than twice that of the amorphous matrix with a Young's modulus of 81 GPa. This difference in Young's modulus promotes void nucleation and coalescence at the interface [190]. Therefore, it is hypothesized that factors such as particle type and size, as well as particle–matrix adhesion, may influence fracture behavior and require further examination in future research.

#### Other-elements-reinforced BMGMCs

**Fig. 24:**
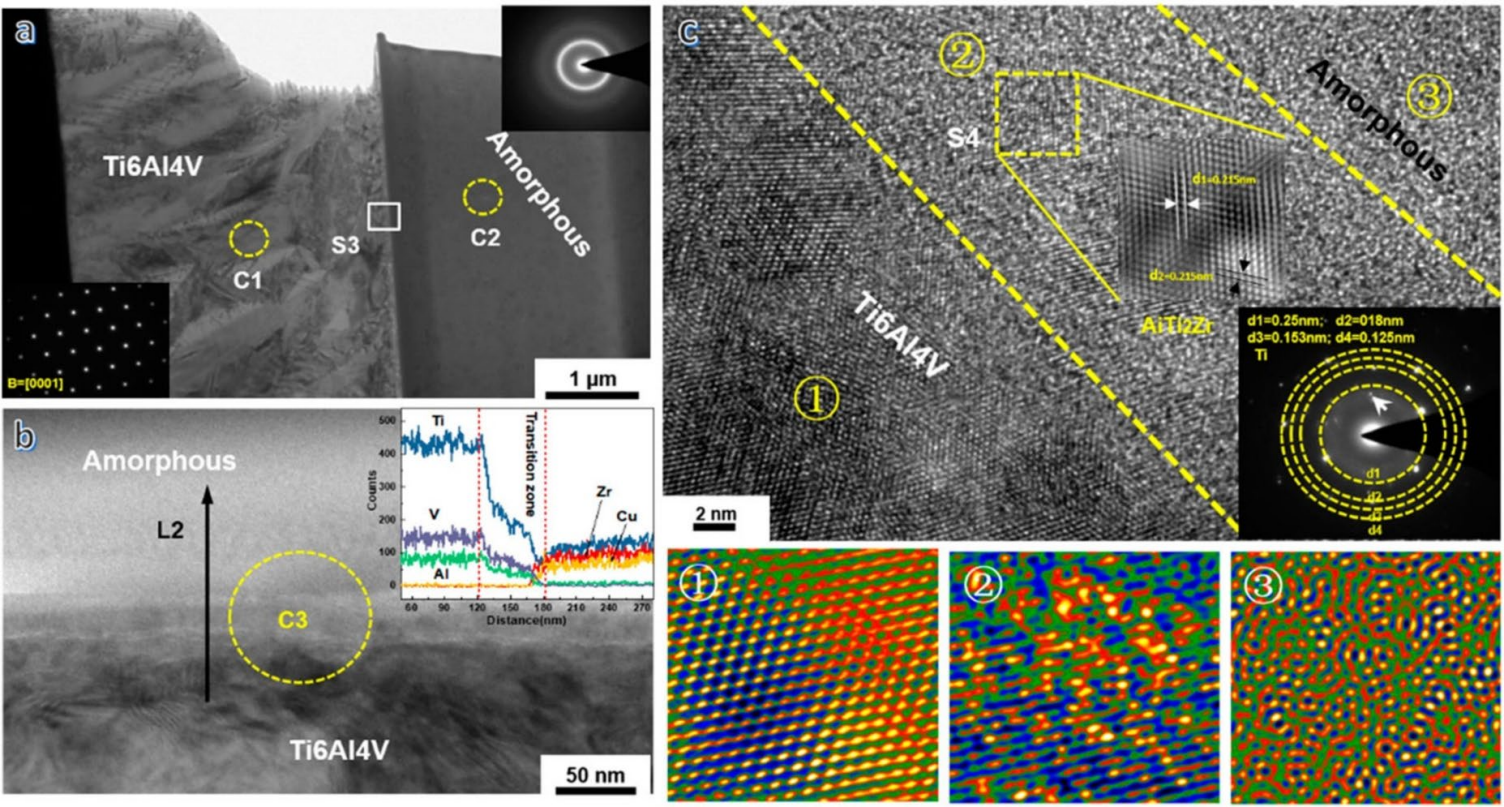
TEM examination on the interface between BMG and Ti_6_Al_4_V frame. (**a**) The interface between BMG and the Ti_6_Al_4_V frame; insets show the SAED, (**b**) bright-field image of Area S3 from (a); the inset shows element distribution along line L2 and (c) HRTEM on the interface between BMG and the frame. The inset shows the diffraction pattern from Area C3. Inverse FFT from Area S4 show the lattice structure of the nanocrystals in the interlayer. The panels at the bottom are the inverse FFT from corresponding areas [174] (reprinted with permission from Taylor & Francis, Copyright © 2020)

The use of ultrasonic vibration–assisted thermoplastic to achieve metallurgical bonding at the interface of Ti_6_Al_4_V frame reinforced Zr_35_Ti_30_Be_26.75_Cu_8.25_ BMG composites was described by Li et al. [174]. The introduction of Ti_6_Al_4_V frame increased the number of SBs and deflected crack propagation, resulting in a substantial improvement in fracture toughness for the produced Zr–BMG composites. In Fig. 24a, a bright-field picture shows a clear connection between the BMG matrix and the Ti_6_Al_4_V frame, confirming metallurgical bonding between the two. The diffraction pattern from region C1, which corresponds to the Ti_6_Al_4_V side, reveals a typical single crystal with a zone axis along [0001]. Conversely, no crystalline diffraction pattern is observed in region C2, indicating the presence of the amorphous BMG matrix. Enlarging the area in Square S3 in Fig. 24a reveals an interlayer at the interface of the BMG matrix and Ti_6_Al_4_V frame, as illustrated in Fig. 24b [174]. Figure 24c shows HRTEM images that provide a more detailed description of Region C3. The interlayer's boundaries, with a thickness of about 60 nm, are clearly visible and marked by dotted lines. Furthermore, regions 1, 2, and 3 are magnified for closer examination. Area 1 displays crystalline grains, while area 3 is entirely amorphous. The presence of both amorphous and crystalline phases in the interlayer is confirmed by the presence of lattice stripes and a labyrinth pattern in Area 2. The diffraction pattern displays a halo ring, indicating the presence of an amorphous phase in the interlayer. In addition, the diffraction pattern from C3 reveals significantly finer nanocrystals of intermetallic complex, which confirms the metallurgical bond between the BMG and Ti_6_Al_4_V frame [174].

In order to prove the effectiveness of metallurgical bonding, the fracture toughness *K*_J_ and bending strength of the BMG composites were measured. The results revealed that while monolithic BMG had a fracture toughness of about 80 MPa.m^½^ and a bending strength of about 780 MPa, the best composite sample had a fracture toughness of 213 MPa.m^½^, which exhibited a 170% increase over monolithic BMG (85 MPa.m^½^), and the bending strength was also enhanced to 1030 MPa [174].

In another study, the boundaries between reinforcement and matrix in tungsten particles reinforced Zr-based amorphous composites with varying infiltrating periods were examined [176]. The time of interfacial reaction, which is closely related to the infiltration time, is a critical factor affecting the performance of composite materials, as the Zr-based alloy melt exhibits reactive wetting behavior toward tungsten particles. Interfacial interactions between the tungsten particles and amorphous matrix facilitated heterogeneous nucleation at the interfaces, leading to crystal formation. As the infiltration time increased, the number of crystals at the boundaries also increased. The interfacial reaction strengthened the interfacial bond, and the small crystals formed at the boundaries exhibited a pinning effect, further enhancing the interfacial strength [176].

**Fig. 25:**
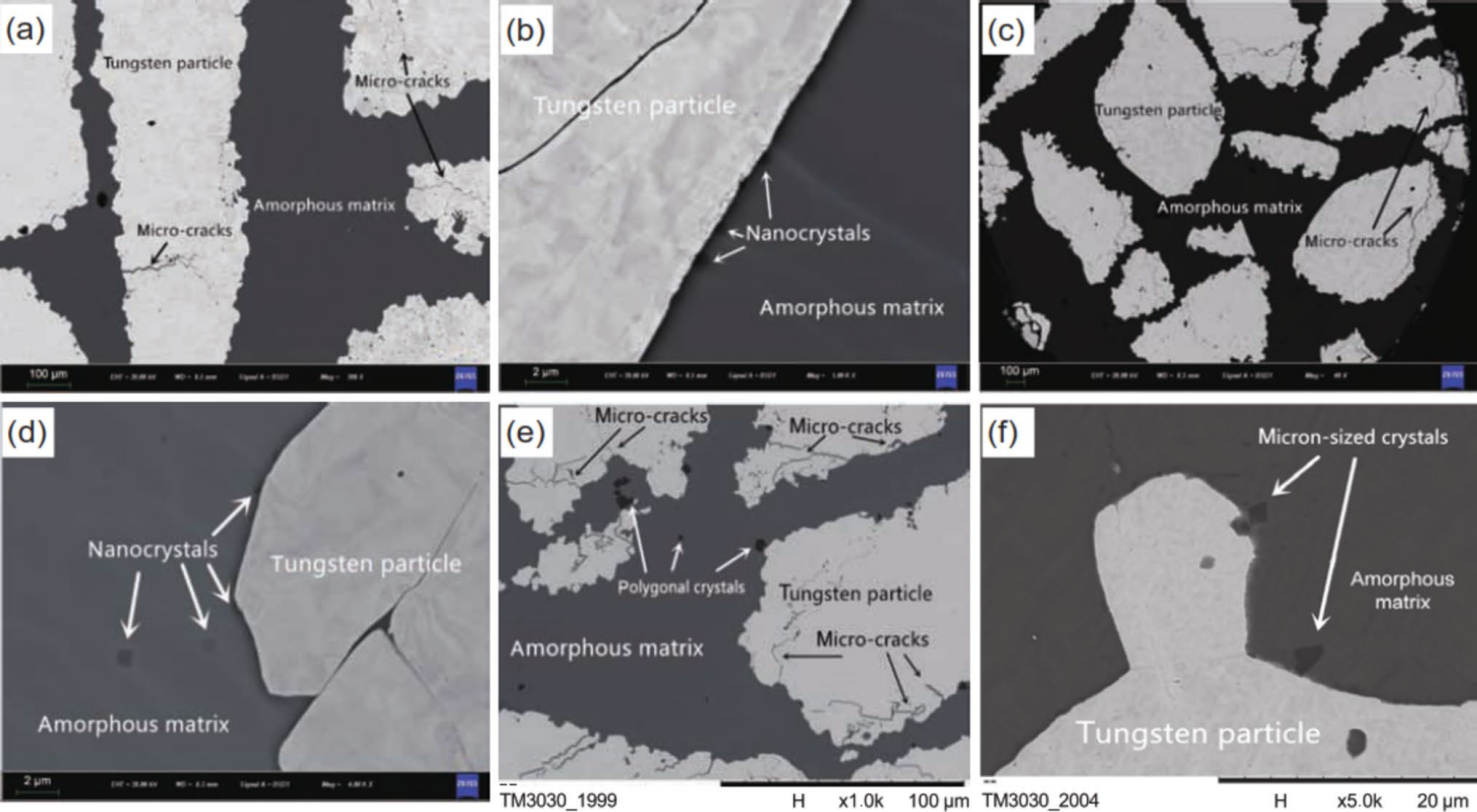
Morphologies of interfacial reaction in tungsten particles reinforced Zr-based BMG composites with different infiltrating times: (**a**, **b**) 1 min; (**c**, **d**) 5 min; (**e**, **f**) 10 min [176] (reprinted with permission from Springer Nature, Copyright © 2020)

Increasing infiltration time resulted in continued heat stress on the tungsten particles, leading to the formation of additional microcracks (Fig. 25a–f). As the number of microcracks increased, the amorphous melt penetrated into them, leading to the creation of more interfaces between the tungsten particles and the amorphous matrix. This, in turn, resulted in a stronger mechanical self-locking effect between the tungsten particles and the amorphous matrix [176].

## Fabrication methods

### Solid state methods

#### Powder metallurgy and sintering

**Fig. 26:**
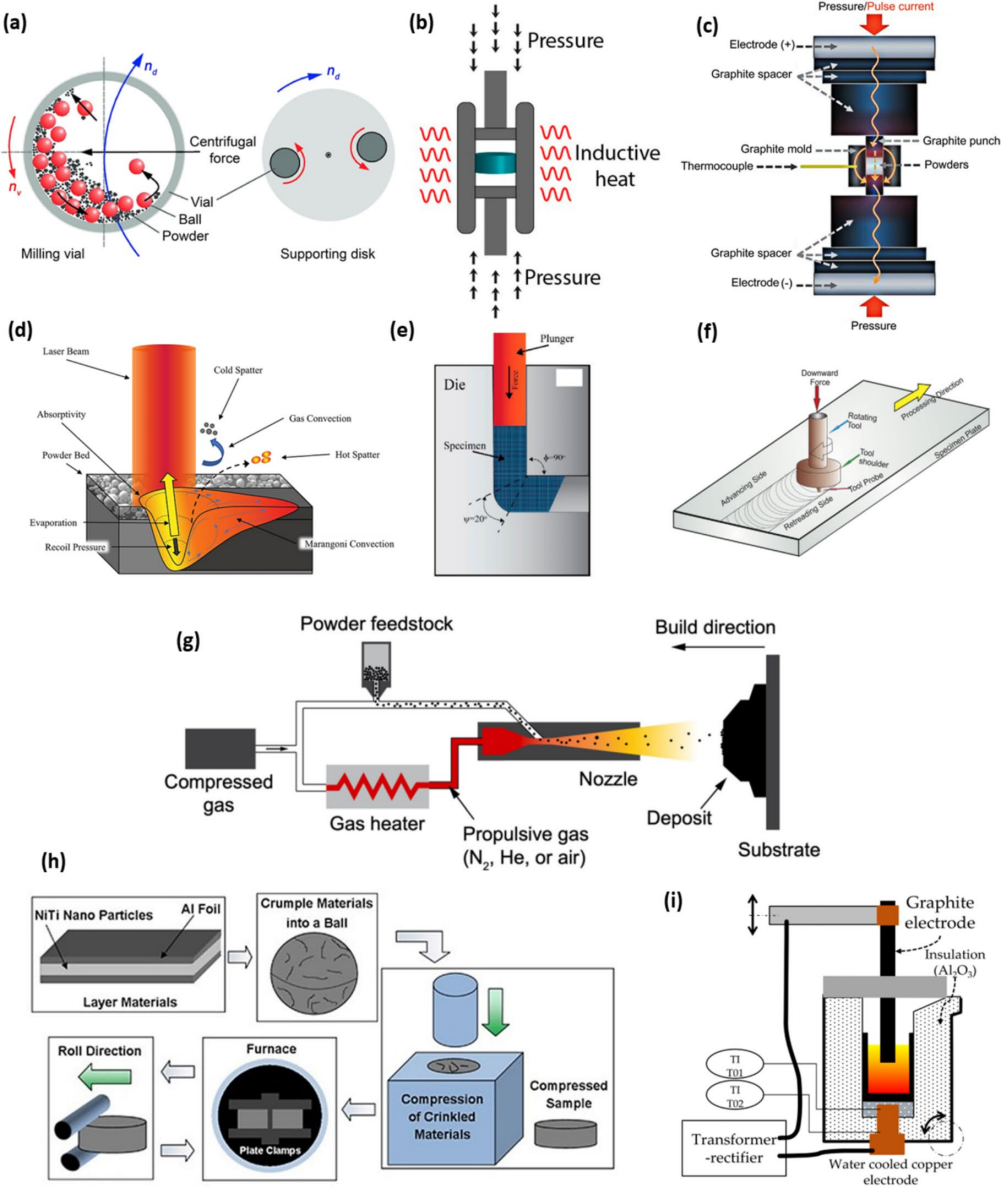
Schematic of (**a**) planetary ball mill [205] (reprinted with permission from Elsevier B.V., Copyright © 2022), (**b**) HP [206] (reprinted with permission from MDPI, Copyright © 2021), (**c**) SPS [207] (reprinted with permission from Elsevier B.V., Copyright © 2017), (**d**) LBPF [208] (reprinted with permission from Elsevier B.V., Copyright © 2023), (**e**) ECAP [209] (reprinted with permission from Elsevier B.V., Copyright © 2022), (**f**) FSP [210] (reprinted with permission from Elsevier B.V., Copyright © 2015), (**g**) cold spray [211] (reprinted with permission from Elsevier B.V., Copyright © 2021), (**h**) RROLM [55] (reprinted with permission from WILEY–VCH, Copyright © 2018) and (**i**) electric arc melting systems [212] (reprinted with permission from MDPI, Copyright © 2020)

Powder metallurgy is a typical solid-state production method for MMCs in which metal powders are usually first blended in a ball milling machine (Fig. 26a), then compacted as a green body. A subsequent heat treatment at elevated temperatures (0.6–0.9 matrix melting temperature) known as sintering is necessary to achieve a dense specimen. Simple CP, cold isostatic pressing, and/or double-pressing double-sintering are all viable methods of powder compaction [200–202]. Additionally, a post-processing step, often hot extrusion, is often required to reduce porosity, refine microstructure, and improve mechanical performance. It would be relatively convenient to regulate the chemical composition, microstructure, porosity level, and distribution of reinforcements in the matrix by optimizing the initial powder size and morphology, applied pressure in compaction, as well as the sintering time and temperature [203, 204]. The process is mostly carried out under a vacuum or an inert atmosphere to prevent oxidation. Additionally, the fabrication of very large parts is not feasible using this method, and impurities can be introduced during both the ball-milling and pressing steps. Moreover, this procedure is time and energy intensive, and the prolonged sintering time (a few hours) may result in abnormal grain growth and deterioration of mechanical properties.

#### Microwave sintering

Microwave sintering is a relatively new method of fabricating MMCs in which the blended raw materials are shaped into a green compact, then the compound is subjected to uniform fast heating that warms the compacted powders volumetrically [213, 214]. Intriguingly, the high power of the microwave field can significantly increase the surface ionization of the particles, resulting in rapid ionic diffusion, particularly at grain boundaries [16]. Consequently, highly compact samples will be prepared with a uniform distribution of grain size just in a few minutes. Yet, ultrahigh local heat generation due to the high microwave absorption capacity of certain materials may give rise to the creation of undesirable phases and/or partial melting of the composite, hence limiting the applicability of microwave sintering [215]. It is also essential to note that microwaves can be exploited for MMCs only when the size of the particles is comparable to the penetration depth of the incident wave, which is on a scale of a few microns. Otherwise, metallic powders will reflect rather than absorb the wave [214].

#### Hot pressing

HP process is capable of producing MMCs with a uniform distribution of the reinforcement phase, high density, and improved mechanical properties. Powders firstly are blended to ensure that a homogeneous distribution of the reinforcement phase is obtained. The prepared powders are shaped into a green body that may be pre-sintered to a certain extent to increase its strength and stability. Then, the green body is simultaneously compacted and sintered to the final product (Fig. 26b). Despite the process occurring at a lower temperature and in less time than conventional sintering, the microstructure and characteristics of the samples are boosted. However, expensive equipment is required, and undesirable reactions between the typically used graphite mold and the sample can also be limiting [27, 216].

#### Spark plasma sintering

SPS is capturing considerable attention for processing high-performance MMCs. In this technique, as a result of pulsed DC voltage in the die assembly and uniaxial pressure in an inert atmosphere or vacuum, the sample undergoes electrical resistance–induced heating (Fig. 26c) [217, 218]. The electrical discharge between particles under applied pressure caused by sparking promotes powder densification. High heating rates (up to 600 K/min) and rapid cooling rate (up to 300 K/min) are the most prominent features of this technology, owing to the non-insulating characteristics of graphite mold, significant radiative loss, and high incoming power. These exceptional characteristics enable the sintering of nanostructured materials with superior mechanical properties. On the other hand, SPS is a binderless technique and does not require a pre-compaction phase. However, the required apparatus is complex and costly, and undesirable reactions may occur between the die and the sample [207].

#### Severe plastic deformation

SPD is one of the most established techniques, which are capable of generating ultra-fine-grained structures within the bulk of metallic composites [219]. There are several commonly used methods of SPD, including equal channel angular pressing (ECAP) [220], accumulative roll bonding [221], HPT [222], and FSP.

In ECAP, a plunger provides a lubricated case with a sufficient driving force to pass through two equal channels intersecting at an oblique angle in the die (Fig. 26e). During the process, the intense shear strain is applied to the sample at the intersection point without changing the dimension of the final sample. Accumulative roll bonding is composed of different steps, including stacking two rolled sheets, heating under the recrystallization temperature, and rolling toward forming a new strengthened single sheet. Then, the samples are halved, and the process is repeated multiple times.

In HPT, there are two anvils with a cavity where a disc-like sample is embedded. In this case, a huge torsional stress is imposed on the sample under high hydrostatic pressure via rotation of the top anvil. FSP is an effective surface modification process for the reinforced MMCs, which will be discussed in the next section in more detail. During the mentioned processes, a significant amount of strain is imposed on the bulk structure through extensive hydrostatic pressure, leading to significant refinement of the microstructure [223]. The reduction in grain size enhances the hardness and strength of the composite through the Hall–Petch law. With this increased hardness, the ultra-fine-grained metallic composites exhibits enhanced wear resistance [224].

Unlike hot extrusion and hot press that require elevated temperatures for the integration of powder-based samples, which mostly damages the secondary phase, some of these techniques such as ECAP and HPT have the great capability to consolidate metallic powders at low temperatures by introducing ultra-high plastic strain into the bulk metallic composite [220]. Another impressive output of ultra-fine-grained composites is promoting the passive film formation on the surface of the sample, which can increase the corrosion resistance in various acidic solutions [225]. Despite many advantages of this process in fabricating highly tough composites, many SPD processes fail to impose uniform strain distribution throughout the specimen [226]. To overcome this problem, multiple passes are required to increase the uniformity through the bulk, which makes the process complicated and inefficient. Besides considerably limiting the geometry of the part to be treated, this process becomes ineffective for refining some brittle metallic alloys with low ductility.

#### Friction stir processing

FSP is a fast, green, and energy-efficient solid-state material processing technique [227–229]. FSP's basic principle is very simple: a rotating tool with a pin and shoulder is placed in a single piece of material, using a sufficiently high pressure to insure contact between the tool and the work piece; the FSP tool is traversed along a pre-defined path to cover the region of interest (Fig. 26f) [230]. Localized heating results from the friction between the tool and the workpiece, causing the latter to soften. The material undergoes significant plastic deformation during FSP, which results in considerable grain refinement [230]. Thus, this method has been successfully employed for microstructural modification of metallic materials [231–233] and fabrication of in situ [154, 234, 235] and ex situ composites [236–238] under severe thermo-mechanical effects. In case of composites manufacturing, the reinforcement powder and the matrix should be put together, for instance, by drilling small holes and filling them with powders of reinforcement material [147]. Besides, by tuning the tool design, FSP parameters, and active cooling/heating, the microstructure and mechanical properties of the processed zone may be precisely controlled. Another advantage offered by this technique is the possibility of controlling the depth of the processed zone by adjusting the length of the tool pin, with the depth ranging from a few hundred micrometers to tens of millimeters [239]. Furthermore, flash flaws and welding thinning can be minimized in this method to retain the integrity of the material [240].

There is an alternative FSP-based method called cooling-assisted FSP that can shorten the peak- and high-temperature residence periods, slowing the movement and migration of grain boundaries in the stir zone and resulting in fine grain structures [241, 242]. Moreover, this updated technique can reduce the risk of the formation of brittle intermetallic compounds at the interfacial area between the reinforcement and the matrix. The process can be performed in a pool of water where the work piece is continuously cooled. However, FSP method has several drawbacks, including limited processing speed, restrictions on the size and shape of the processed material, and high equipment costs, which make it challenging to employ this technique for large-scale industrial applications.

#### Friction deposition

This is a relatively simple solid-state additive manufacturing technique facilitated using a simple friction welding machine [243]. The basis of this technique is similar to friction stir welding, with a difference that a solid tool is used and friction deposition is realized by means of a hollow shoulder in which the feedstock material is inserted. Heat is generated by friction at the interface of the shoulder, the feedstock material, and the work piece, as the shoulder continues to rotate at a high speed. Consumable material is fed through the shoulder and gets deposited on the work pieces, leading to the generation of layers of the deposited material. As the feedstock material is pushed out of the shoulder, heat is transferred through it by conduction causing the feed material to be softened. One distinguishing aspect of this new technique is the thickness of individual deposited layer, which is significantly higher than any other additive manufacturing method [244]. Karthik et al. [161] used this technique for additive manufacturing of Al–matrix reinforced composites with HEAs for the first time.

#### Cold spray deposition

Cold spray deposition is an effective solid-state process commonly used also for the fabrication of MMC coatings. In this technique, powders of the metal matrix and the reinforcement material (metal or ceramic) are accelerated to supersonic velocities using a pre-heated gas (commonly nitrogen or helium) passing through a de-Laval nozzle to impact a substrate [211]. If the impact velocity is high enough, the particles bond to the substrate, avoiding the need to melt the materials (Fig. 26g). Therefore, high-temperature consequences like phase transformation, tensile residual stresses, and oxidation can be prevented [245, 246]. The deposition in cold spray is achieved through a combination of mechanical interlocking, deformation, and metallurgical bonding mechanisms. The cold spray process is advantageous for MMC fabrication since it is not based on melting and solidification and thus is not fully bound to the thermal compatibility of the mixed materials. Once the spray parameters are well optimized, low porosity deposits with high cohesive strength can be obtained. The cold spray process can be used to fabricate MMCs with a variety of reinforcements; however, it has not been vastly used and much research is still required to discover the full potential of this technique for next-generation MMCs. How much each phase contributes to the material's overall load-bearing capacity is arguably the most important question regarding the mechanical properties of cold sprayed MMCs. Despite the fact that the collective response to external load has been extensively investigated in the field, the phase-specific response of cold spray MMCs is much less understood.

#### Rolling of randomly oriented layer-wise materials

RROLM is a unique manufacturing technology, which enables the formation of a layer-wise aligned distribution of micro-scale intermetallic particles in the matrix (Fig. 26h). This method can be used to obtain an AMC with both high strength and toughness. The process consists of combining foil from materials with nanoparticles to form in situ layered, discontinuous reinforcements within a bulk, ductile material. Under heating through solid state diffusion, nanoparticles permeate into the foil layers causing the formation of high strength intermetallics and production of a hierarchical, layer-wise structured metal matrix composite [55].

### Liquid state methods

#### Laser powder bed fusion

MMCs can be created using the advanced additive manufacturing technique known as SLM or laser powder bed fusion (LPBF). By layer-by-layer melting and fusing metallic powder particles, a high-powered laser beam is used in the SLM process to create three-dimensional objects (Fig. 26d). Complex forms and geometries can be produced by accurately controlling the laser to melt only the appropriate areas of the powder bed. A mixture of metal powder and reinforcing phase must first be made in order to produce MMCs using this technique. The mixture is then loaded in the system, spread on the substrate bed, and then the laser is utilized to selectively melt and fuse the metal powder, fusing the reinforcing phase into the metal matrix [247]. The ability to exert strict control over the distribution and orientation of the reinforcing phase inside the metal matrix is the fundamental benefit of employing SLM for MMC manufacturing. This can result in improved mechanical properties, such as increased strength, stiffness, and wear resistance. Additionally, the SLM process makes it possible to create MMCs with complicated internal structures and geometries, which would be challenging or impossible to do with conventional production techniques [248, 249].

#### Electric arc melting

In arc melting, the alloys are melted under vacuum or inert atmosphere employing an electric arc between an electrode and a water-cooled ground plate (hearth, also called a crucible, usually made of copper) (Fig. 26i). The electrode may be either non-consumable (water cooled and conventionally made of tungsten) or consumable, made of Ti or Ti alloys. The arc melting process is able to produce the highest purity ingots; however, it faces major challenges for upscaling, thus it is commonly used in laboratories to prepare reference samples of the alloys. Another disadvantage of this technology is the non-homogeneity of the obtained ingot, which requires several remelting procedures and, in many cases, long annealing times [250].

#### Induction melting

Induction melting is an effective method for melting high-purity metals such as Ti, Nb, and Mo [1–7]. The energy for melting is transferred via the electromagnetic field generated by an induction working coil. This electromagnetic field induces an eddy current, which results in Joule heating in charged metals. Generally, a water-cooled metallic crucible, i.e., a cold crucible, is used to prevent contamination. When molten metal contacts the inner wall of the cold crucible, the melt is solidified immediately and a solid crust, the so-called skull, is formed between the melt and the wall. This skull prevents contamination from the crucible and protects the crucible against the hot melt [251]. However, this process shows poor energy efficiency since a large portion of the input energy is lost to the water-cooled crucible [252]. Thus, in an attempt to minimize the loss, a metallic crucible has been slitted and several segments are isolated electrically from each other to improve the energy efficiency by providing sufficient electromagnetic transparency of the crucible [252]. This is a promising method for medium- to large-scale manufacturing of castings with the advantages of being simple, allowing for homogeneous stirring of the melt and the ability to maintain a high superheat to enable quality castings to be obtained at large scale and reduced costs [250]. A summary of all these fabrication methods is provided in Table 5.

**Table 5:** Summary of various preparation methods of MMCs, their advantages and disadvantages

Fabrication method	Description	Advantages	Disadvantages
Powder metallurgy and sintering	Metal powders are blended, compacted, and sintered under elevated temperatures to achieve dense composites. Often requires subsequent hot extrusion	– Control over chemical composition and microstructure – Suitable for small to medium-sized parts – Potential for high mechanical performance – Suitable for complex shapes	– Time and energy intensive – Limited to smaller parts – Possible introduction of impurities – Prolonged sintering times can degrade properties
Microwave sintering	Raw materials are compacted and rapidly heated volumetrically using microwaves	– Rapid processing time – Uniform grain size distribution – Enhanced ionic diffusion at grain boundaries	– Limited to materials with high microwave absorption – Potential for undesirable phase formation – Particle size must match microwave penetration depth
Hot pressing (HP)	Powders are homogeneously blended and simultaneously compacted and sintered under pressure and temperature	– Uniform reinforcement distribution – Improved mechanical properties – Lower processing time compared to conventional sintering	– Expensive equipment required – Potential for undesirable reactions with mold materials – Limited to smaller parts
Spark plasma sintering (SPS)	Powders are densified under pulsed DC voltage and pressure in an inert atmosphere	– High heating and cooling rates – Binderless process – Suitable for nanostructured materials – High mechanical properties	– Complex and costly equipment – Possible undesirable reactions with die material – Limited to smaller parts
Severe plastic deformation (SPD)	Processes like ECAP, HPT, etc. impose high plastic strain to refine microstructure	– Ultra-fine-grained structures – Enhanced hardness and strength – Low-temperature consolidation for certain alloys – Improved wear resistance and corrosion resistance	– Requires multiple passes for uniform strain – Complex and inefficient for some materials – Limited to specific geometries and materials
Friction stir processing (FSP)	A rotating tool mechanically stirs and refines the microstructure of materials	– Green and energy efficient – Precise control over microstructure and mechanical properties – Minimal defects like flash flaws	– Limited processing speed – Constraints on material size and shape – High equipment costs
Friction deposition	Solid-state additive manufacturing using friction welding to deposit layers of material	– Higher deposition rate compared to other additive methods – Suitable for complex geometries – Can handle high thickness layers	– Limited to certain materials and applications – Requires initial powder mixing and tooling adjustments
Cold spray deposition	Metal powders accelerated to supersonic velocities impact a substrate to form coatings or bulk materials	– No melting required, avoiding thermal issues – High bond strength and low porosity – Suitable for various reinforcements	– Limited research and development – Optimization needed for different material combinations – High initial investment
Rolling of randomly oriented layer-wise materials (RROLM)	Foil and nanoparticles are layered and heated to create intermetallics within a ductile matrix	– High strength and toughness – Hierarchical layer-wise structure – Uniform distribution of nanoparticles	– Requires specific foil and nanoparticle combinations – Complex heating and layering process – Limited to certain applications
Laser powder bed fusion (LPBF)	Metal powders melted layer-by-layer using a high-powered laser beam to create complex structures	– Precision in reinforcement distribution – Complex internal geometries – High mechanical properties	– Limited by material compatibility with laser melting – High equipment and operating costs – Post-processing challenges
Electric arc melting	Alloys melted under vacuum or inert atmosphere using an electric arc	– High-purity ingots – Effective in laboratories for alloy preparation	– Challenges in scaling up – Non-homogeneous ingots may require multiple remelting – Long annealing times
Induction melting	Electromagnetic field induction melts metals in a cold crucible for high-purity casting	– Homogeneous stirring of the melt – High superheat capabilities – Suitable for medium- to large-scale manufacturing	– Poor energy efficiency due to crucible losses – Initial investment in energy-efficient crucibles

## Potential applications

The applications are normally defined based on the matrix of a composite. Therefore, potential applications include all known fields of applications for metallic alloys, remarking that the performance of MMCs reinforced with metal particles may be better than conventional ones. Generally, this class of MMCs have been introduced to a range of prospective structural applications due to their unique mechanical, physical, and chemical properties. However, these new generations of MMCs also have great potential for functional applications that require further research. By exploring the potential applications of MMCs, we can develop new materials with fascinating properties, leading to a wide range of interesting research opportunities.

### Structural applications

#### Aerospace

When it comes to building aircraft, selecting the right material is crucial. Metals like Al, Ti, and steel are commonly used due to their lightweight nature, high strength, and resistance to corrosion, fatigue, heat, and cracks [253]. However, these metals need to be strengthened to ensure safety during flight. Alloying and employing special fabrication techniques or post-treatments are normally utilized to meet the requirements. The new generation of MMCs, reinforced by metal particles, have the potential to be used in the production of structural components providing greater durability, high resistance to wear and corrosion, and lower weight, which may contribute to improved fuel efficiency and reduced maintenance costs in aerospace applications. In addition to structural components for aircraft, these MMCs can also be used in satellite components such as antenna and reflectors that require high stiffness and thermal stability [254–257]. Furthermore, there are a variety of specific applications for SMA composites including actuators to control various components of an aircraft or spacecraft, morphing wings that can change their shape in-flight, allowing for higher aerodynamic efficiency and improved fuel economy, and active vibration control systems to reduce vibrations in aircraft and spacecraft, improving ride comfort and reducing component wear [258–260]. While the use of MMCs in aerospace applications exhibits a high potential to improve the performance and sustainability of aircraft, producing the next generation of MMCs for the aerospace industry can be complex and expensive, requiring specialized manufacturing techniques.

#### Automotive

Metal materials have been a staple in the automotive industry for their strength, durability, and affordability. However, as the industry shifts toward more sustainable practices, there is a growing demand for lighter and more fuel-efficient vehicles. This has led to the development of high-strength lightweight metals such as aluminum and magnesium, which are increasingly being used in the production of car parts. To further reduce weight while maintaining strength, composite materials have become a popular choice in the automotive industry. These materials offer unique advantages over traditional metals, such as improved corrosion resistance and greater design flexibility. Composite materials can be used to produce engine components, brake systems, suspension, body and chassis components, and other critical parts that require both lightweight and high-strength properties. In addition to improving fuel economy and reducing emissions, the use of composite materials in cars also offers better crash resistance, longer service lifetime, and higher reliability. Furthermore, the use of metal particles as reinforcement in composite materials can enhance the corrosion resistance of car parts that are exposed to harsh environments. These benefits make composite materials an attractive choice for car manufacturers aiming to produce more sustainable and efficient vehicles [261–263].

#### Sports and recreation

Metallic materials and composites, including magnesium, titanium, and aluminum, are commonly used in the production of high-performance sporting goods due to their high strength-to-weight ratio, impact resistance, and stiffness. However, magnesium is susceptible to corrosion, which can be addressed by coatings or incorporating novel metallic particles. Titanium is an excellent choice for golf clubs due to its vibration characteristics and stiffness, but the addition of BMG particles can improve energy absorption and transfer of impact to the ball. Aluminum's soft nature can also be improved by reinforcing it with metal particles for enhanced performance in various sports equipment. The use of MMCs in manufacturing sports equipment such as bicycles, golf clubs, and tennis rackets offers improved strength, reduced weight, better shock resistance, and improved safety for athletes [264, 265].

### Functional applications

#### Electronics

In recent years, the electronics industry has made remarkable progress, and this can be attributed to the advancement in materials used in their construction. These materials have contributed to the miniaturization, enhanced speed, and improved reliability of electronic components. High-quality electronic metals have become a popular choice in the manufacturing of various electronic appliances, as they offer exceptional conductivity and corrosion resistance. Additionally, the materials used in electronics must be flexible, versatile, and precisely manipulated to meet the demands of modern technology. One of the biggest challenges facing electronic equipment is heat generation. The materials used must be able to withstand high temperatures and resist the effects of the harsh environment. New-generation MMCs can be a part of advanced materials for next-generation electronics with higher performance. They could be particularly useful in the manufacturing of heat sinks due to their high thermal conductivity. Moreover, substrates with high dimensional stability can be machined to tight tolerances, which makes them perfect for use in electronics. These materials can be designed to provide low CTE, which means that they can maintain their shape even when exposed to high temperatures. In addition to managing heat, the electronics industry also faces the challenge of electromagnetic interference (EMI), which can disrupt the normal functioning of electronic devices and cause significant damage [266]. To mitigate EMI, electromagnetic shielding materials can be used to absorb or reflect electromagnetic radiation, reducing the risk of interference [267, 268]. While copper and aluminum are effective shielding materials, their susceptibility to corrosion limits their usage. Embedding metal particles into these materials can partially address this concern. Although components such as copper and aluminum provide adequate shielding against EMI, their susceptibility to corrosion limits their usage [266]. Incorporating metal particles into these metals may mitigate this issue to some extent [269]. Although there is ample space to utilize MMCs in electronic industry, extended research is needed to better explore the behavior of metal particle reinforced MMCs in electronic devices.

#### Energy conversion

**Fig. 27:**
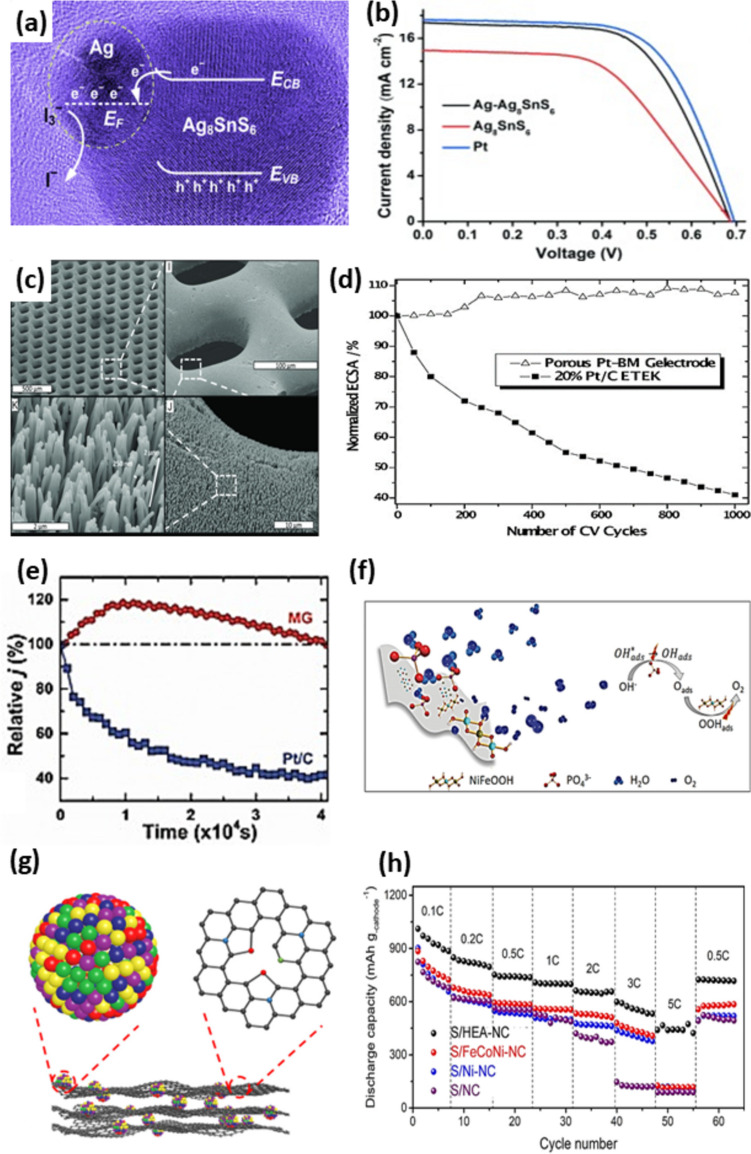
(**a**) A Mott–Schottky-type Ag–Ag_8_SnS_6_ heterodimer as counter electrode in a DSSC [274] (reprinted with permission from WILEY–VCH, Copyright © 2015). (**b**) Current density–voltage characteristics of DSSCs with Ag-Ag_8_SnS_6_, Ag_8_SnS_6_, and Pt counter electrodes [274] (reprinted with permission from WILEY–VCH, Copyright © 2015). (**c**) SEM images of Pt-BMG nanowire used as the electrode of a micro fuel cell [275] (reprinted with permission from WILEY–VCH, Copyright © 2013). (**d**) Loss of the electrochemical surface area after accelerated durability test of porous Pt-BMG electrode of a microfuel cell and Pt/C (E-TEK) catalysts with the number of cyclic voltammogram cycles [275] (reprinted with permission from WILEY–VCH, Copyright © 2013). (**e**) Durability of the metallic glass catalyst, in comparison to the Pt/C catalyst for OER application [278] (reprinted with permission from WILEY–VCH, Copyright © 2016). (**f**) Schematic illustrating the working mechanism of amorphous bulk NiFeP for OER application [279] (reprinted with permission from WILEY–VCH, Copyright © 2017). (**g**) Schematic illustration of HEA–nitrogen doped carbon as the cathode of Li–S battery [280] (reprinted with permission from WILEY–VCH, Copyright © 2022). (**h**) Rate capability of the S/HEA–nitrogen doped carbon cathode Li–S battery [280] (reprinted with permission from WILEY–VCH, Copyright © 2022)

With the increasing industrialization of the world, the importance of energy conversion is becoming more evident as it enables the transformation of energy from forms provided by nature into usable forms of energy [270, 271]. The potential of novel materials in advancing energy conversion applications promises a cleaner and more efficient energy future [272, 273]. Metallic materials play vital roles as catalysts, electrode materials, conductors, and structural components in this process. Notably, by leveraging MMCs, such as embedding Ag nanoparticles in Ag_8_SnS_6_, we can fabricate metal–semiconductor heterodimers, catalyzing redox reactions in dye-sensitized solar cells [274]. This approach is capable of improving the efficiency and stability of the solar cells, giving rise to more powerful cells with longer lifespan (Fig. 27a, b). In another attempt, a Pt-based MG was used as the catalytic electrode layer in a proton exchange membrane, and its performance was compared to that of a 20% Pt/C electrode [275]. The results showed that the BMG electrode was highly active and durable, indicating its potential as a viable alternative to conventional Pt/C electrodes (Fig. 27c, d) [275]. In addition, promising results were obtained from testing Pt- and Pd-based BMGs as electrocatalysts for various types of fuel cells, including alkaline fuel cells, direct methanol fuel cells, and direct ethanol fuel cells [276, 277]. These studies have shown that BMGs can act as effective catalysts for fuel cell applications due to their high surface area, good catalytic activity, and stability under harsh operating conditions. Therefore, in fuel cells, costly noble-metal catalysts could be substituted with BMGs to not only reduce costs but also improve performance [276].

Due to the high electrochemical activity of BMGs in the hydrogen evolution reaction (HER), there is a great opportunity to benefit from BMGs in hydrogen gas production technologies. Moreover, their high electronic conductivity and corrosion resistance, stemming from the absence of crystalline defects, make them an excellent candidate for this application [281]. Several studies have explored the use of BMGs in this context, including Pd–Ni–Cu–P, which displayed outstanding self-stabilizing behavior and outperformed commercial Pt/C catalysts (Fig. 27e) [278]. This improved performance is attributed to the inherent structural heterogeneity on the amorphous surface, improved electron transport, and selective dealloying during the chemical reaction [278]. To replace noble metals, such as Pt, Ir, and Ru, a conductive medium not prone to degradation is required in the oxygen evolution reaction (OER), which is the anodic reaction in the hydrogen gas production process. NiFeP metallic glass samples with various Ni/Fe ratios have exhibited excellent OER activity due to their high electronic conductivity and abundant active sites provided by the coordinated Ni and Fe (Fig. 27f) [279].

Moreover, developing self-healing MMCs could positively impact the wind turbine industry by reducing maintenance expenses, extending service lifetime, and preventing catastrophic failures. With self-healing properties, the MMCs could repair themselves in case of damage, thus increasing their durability and reliability. This could result in lower operating costs and improved safety for wind turbines, which are critical components of renewable energy systems [282]. Engaging these metallic materials in energy applications has relatively emerged in the recent years, and ongoing research is crucial to fully explore their vast potential.

#### Energy storage

Energy storage, along the energy conversion, stands as a pivotal field driving innovation in renewable energy utilization [283–285]. By leveraging principles of electrochemistry and advanced materials science, researchers aim to develop efficient, scalable, and environmentally sustainable energy storage solutions [286, 287]. Recent advancements in metallic components hold significant promise for enhancing energy storage technologies, particularly batteries and supercapacitors [288–291]. For instance, the utilization of HEAs in battery electrodes has demonstrated notable electrochemical activity attributed to lattice distortions, rendering them well suited for next-generation batteries (Fig. 27g, h) [280]. A strategic approach to further enhancing electrochemical activity could involve incorporating metal particles into the porous structure of HEAs, potentially yielding composite materials with improved conductivity, stability, and enhanced ion/electron transport [292]. This approach also facilitates increased accessibility of electrolyte ions to internal active sites, offering a promising avenue for future research in battery and supercapacitor technologies.

#### Electromagnetic interface shielding

Electromagnetic interference (EMI) shielding refers to the process of reducing the EMI emitted by electronic devices or systems, thereby preventing them from interfering with nearby electronic equipment or communication systems [293, 294]. Metallic components can serve as effective EMI shielding materials due to their ability to provide reflection, arising from mobile charge carriers, and absorption, arising from electric and/or magnetic dipoles, thus safeguarding electronic systems from external electromagnetic disturbances [295–298]. Hence, ferromagnetic alloys, such as Fe-, Co-, and Ni-based BMG or HEA, could exhibit superior performance for EMI applications [299, 300]. In one study, Co-based BMG showed promising potential as thin and flexible materials capable of efficient EMI shielding, offering excellent soft magnetic properties, satisfactory shielding capabilities, and mechanical robustness [300]. Integration of metal particles into MMCs may further enhance their electromagnetic properties, thereby bolstering their utility in EMI shielding applications across various industries, including electronics, telecommunications, and aerospace.

#### Medical

High performance MMCs have a wide range of potential applications in the biomedical industry. The most promising applications are orthopedic and dental implants as high strength and stiffness of MMCs can help in reducing the risk of implant failure, while their resistance to wear and corrosion can improve the longevity of the implant [301–303]. Moreover, integrating silver nanoparticles into metal matrices can confer antibacterial, antimicrobial, and antifungal properties [304–306]. They interact with pathogens by disrupting cell membranes, inhibiting cellular processes, and inducing oxidative stress, effectively preventing microbial growth and proliferation. Another important potential application is related to the use of SMA composites in the production of stents that can expand and contract to adapt to the shape of the artery. This can improve the long-term effectiveness of the stent and reduce the risk of complications. SMA composites can also be used in the production of surgical instruments and tools. These materials can provide improved performance due to their super-elasticity and SME, allowing the instruments to adapt to the shape of the tissue or organ being operated on. This option can open new doors to improve the precision and effectiveness of surgical procedures [307–309].

## Challenges and prospects

MMCs reinforced by metal particles have been popular since the early 2000s, with Ti, Cu, and some intermetallic compounds being the preferred reinforcement choices. Later, with the significant progress in the field of amorphous metals, they became the favored reinforcement phase for soft metal matrices, yielding excellent mechanical performance. By the mid-2010s, scientists began exploring the potential of using HEA particles as reinforcement for metal matrices due to their excellent mechanical properties and stability in monolithic form.

Reviewing the state of the art in this area, it is noted that while using metal particles as the reinforcement phase in MMCs can offer several advantages, there are still significant challenges that must be tackled before we can take full benefit of their potentials. For instance, the proper selection of elements and optimization of their composition are critical factors for complex alloys such as BMGs and HEAs. In this regard, the utilization of thermodynamic modeling and computational simulations such as molecular dynamics, Monte Carlo simulations, and neural network modeling can significantly enhance the quality of material design. Another significant challenge is to achieve strong bonding between the reinforcing metal particles and the metal matrix, as it can promote effective load transfer and thus improve the composite's strength. Typically employed strategies include (i) surface modification of the reinforcing particles through chemical treatments, oxidation, and coating; (ii) alloying the metal matrix with elements that have better affinity to the reinforcing particles; (iii) in situ formation of the reinforcement; and (iv) mechanical interlocking through ball milling or compaction through shot peening.

On the other hand, producing high-performance MMCs requires cutting-edge technologies. Fabrication methods pose several challenges, including difficulties in achieving consistent mixing, maintaining uniform temperature distribution during processing, and controlling the cooling rate of the composite. These aspects are of utmost importance, for instance, in case of advanced techniques such as SPS, LPBF, and FSP in which any slight change in the influential factors can significantly impact the interface thickness, formed phases, and the final performance. The currently used techniques are of high cost and encounter multiple technological limitations regarding the possible material combinations, and the sample size that limit the advancement of this field.

Another important challenge that should be tackled to facilitate the diffusion of MMCs in demanding fields like automotive, aerospace, and electronics is the ambiguity around their corrosion behavior, considering the complexity of deconvoluting the individual roles of various influencing parameters including the type of matrix and reinforcement materials, processing methods, bonding state, grain size range and homogeneity, and the environmental conditions.

Despite these scientific and technological gaps, the high technological and environmental impact of this area of research has attracted considerable attention. Researchers from various disciplines, such as chemistry, physics, electronics, and engineering, have taken an interest in MMCs trying to explore their performance in novel applications such as solar cells, batteries, and water splitting, expanding the applications of this field through a multidisciplinary approach. Further investigations are necessary to examine various properties of these materials, such as their electronic and heat conductivity, which are essential for new applications in the energy field. Theoretical research and density functional theory (DFT) studies are also critical to understand the electronic structure and the movement of charge carriers in these materials, especially at the interfaces; the acting mechanisms of interaction with other substances in catalytic applications is also another open area of research.

Except for DFT and computational methods, there are two main remedies that have assisted other fields of science and engineering but have not received much attention in the field of composites. One missing approach is the category of in situ and operando techniques, which have become increasingly popular for evaluating materials intended for advanced applications, such as energy devices. These techniques can provide valuable insights into the behavior and performance of MMCs. For example, in situ XRD can be used to study the crystal structure and phase evolution of the reinforcement phase in an MMC during manufacturing. This technique can provide real-time information on the formation of intermetallic compounds and the growth of the reinforcement phase, which can inform the design and processing of the composite. They can be also used to analyze the load-bearing share and interaction of various phases under loading. In situ TEM is another technique that can be used to assess the microstructure and mechanical behavior of MMCs under various conditions, such as high temperature or mechanical loading. This technique can provide insights into the deformation mechanisms of the composite, the evolution of defects and dislocations, and the role of the reinforcement phase in strengthening the composite. In situ thermo-mechanical analysis can be used to study the thermal and mechanical behavior of MMCs under heating, cooling, or mechanical loading. This technique can provide information on the CTE, elastic modulus, and thermal stability of the composite, which can inform and level up the design and processing of the composite. Lastly, in situ electrochemical impedance spectroscopy can be used to study the corrosion behavior of MMCs under various conditions, such as exposure to corrosive environments or mechanical loading. This technique can provide valuable information on corrosion rate, corrosion products, and corrosion mechanisms of the composite, and thus can shed light on the currently ambiguous aspects of the electrochemical performance of these composites.

In addition, machine learning is a powerful tool that can contribute significantly to the development of MMCs reinforced by metal particles through various material design approaches. By analyzing large datasets of material properties, machine learning algorithms can be used to identify the optimal combination of different materials for both the matrix and reinforcement phases in a composite. This has a high potential to facilitate predicting the desired mechanical and physical properties of the final composite as a function of various process and material parameters (e.g., chemical composition, morphological aspects, distribution and volume fractions) and thus not only accelerate the design process, but also enhance its fidelity. In addition to material design, machine learning algorithms can also offer a significant boost in optimizing the MMCs’ manufacturing processes. By analyzing data from various manufacturing steps, such as mixing, shaping, and sintering, these algorithms can identify the optimal parameters that will result in high-quality composites. These algorithms can be shaped to guarantee the desired performance indexes for specific applications.

With the advancement of modelling and analysis tools and by developing a multidisciplinary approach, these challenges are expected to be tackled, empowering MMCs reinforced by metal particles as high-performance materials with significant properties and unprecedented performance, creating great opportunities for demanding industries. A summary of these observations and challenges is provided in Table 6.

**Table 6:** Main observations and challenges in MMCs reinforced with metal particles

Category	Main observations	Challenges
Reinforcement evolution	– Early 2000s: Ti, Cu, and intermetallic compounds were popular – Then, amorphous metals utilized for soft matrices – 2010s: HEA particles explored for superior properties	– Lower performance of metal particles in comparison to ceramic reinforcements
Material design	– BMGs and HEAs synthesized in hundreds of compositions – In situ formation of metal reinforcements improved interface properties	– Difficulty in selecting the optimal elements and compositions for complex alloys – Tailoring the interface bonding
Fabrication methods	– Advanced methods such as SPS, LPBF, and FSP allowed for high-performance MMCs	– Inconsistent mixing, temperature control, and cooling rate challenges – High cost of current technologies – Limitations on material combinations and sample size
Characterization	– Employing advanced techniques enabled better understanding of the interface phenomena between the matrix and reinforcement	– In-depth knowledge of the behavior of novel synthesized components – Lack of numerical models predicting properties of complex MMCs
Multidisciplinary approach	– Growing interest from fields such as chemistry, physics, electronics, energy conversion and storage, and biomedical engineering	– Limited knowledge on electronic and thermal properties, as well as biocompatibility of novel MMCs

## Conclusions

In this paper, the next generation of MMCs that are reinforced by metal particles instead of conventional ceramics are reviewed and analyzed. The four major categories of metallic reinforcement materials, including intermetallics, BMGs, HEAs, SMA, and other metals, have been introduced as the main reinforcement options demonstrating excellent physico-mechanical properties that surpass conventional MMCs. The specific features, advantages, and complications have been extensively discussed for each category and their current and future range of applications are described.

The main advantage of metal reinforced MMCs is that the higher affinity of the matrix and the reinforcing particles thanks to their metallic nature leads to more uniform and coherent matrix/reinforcement interfaces, reducing the risk of debonding upon loading.

There are still numerous gaps regarding MMCs' material design and characterization as well as their fabrication that should be bridged to facilitate the application of these advanced materials. Nevertheless, the recent advancements in MMCs' technology open up unique opportunities for developing new metallic components that meet the requirements of various high-tech industries, including automotive, aerospace, sporting goods, electronics, energy devices, and biomedical sectors. The promising outcomes obtained from these MMCs are paving a new path toward next-generation high-performance MMCs indicating that a revolution in materials science and engineering in the near future.

## Data Availability

No data has been used for this review paper.

## Abbreviations

AMC: Aluminum matrix composite
BM: Base material
BMG: Bulk metallic glass
BMGMC: Bulk metallic glass matrix composite
CP: Cold pressing
CS: Cold spray
CSS: Core-shell structured
CTE: Coefficient of thermal expansion
EDS: Energy-dispersive X-ray spectroscopy
ECAP: Equal channel angular pressing
EMI: Electromagnetic interface
EPMA: Electron probe microanalysis
FFT: Fast Fourier transformation
FIB: Focused ion beam
FSP: Friction stir processing
HAZ: Heat affected zone
HEA: High entropy alloy
HER: Hydrogen evolution reaction
HP: Hot pressing
HPT: High-pressure torsion
HRTEM: High-resolution transmission electron microscope
IPF: Inverse pole figure
LPBF: Laser power bed fusion
MG: Metallic glass
MMC: Metal matrix composite
OER: Oxygen evolution reaction
PSN: Particle stimulated nucleation
RROLM: Rolling of randomly oriented layer-wise materials
SAED: Selected-area electron diffraction
SB: Shear band
SEM: Scanning electron microscope
SMA: Shape memory alloy
SME: Shape memory effect
SPD: Severe plastic deformation
SPS: Spark plasma sintering
SZ: Stir zone
TMAZ: Thermo-mechanically affected zone
UTS: Ultimate Tensile Strength
YS: Yield Strength
